# CuH-catalysed hydroamination of arylalkynes with hydroxylamine esters – a computational scrutiny of rival mechanistic pathways†
†Electronic supplementary information (ESI) available: Complete account of all examined pathways, computational details, full description of reported key species (energies and Cartesian coordinates in angstroms). See DOI: 10.1039/c7sc01107e



**DOI:** 10.1039/c7sc01107e

**Published:** 2017-04-28

**Authors:** Sven Tobisch

**Affiliations:** a University of St Andrews , School of Chemistry , Purdie Building, North Haugh, St Andrews , UK KY16 9ST . Email: st40@st-andrews.ac.uk

## Abstract

An in-depth computational mechanistic probe of the CuH-mediated hydroamination of internal arylalkynes with an archetype hydroxylamine ester and hydrosilane by a (Xantphos)CuH catalyst (Xantphos ≡ {P^P} ≡ 4,5-bis(diphenylphosphino)-9,9-dimethylxanthene) is presented. This first comprehensive computational study of the CuH-mediated electrophilic alkyne hydroamination has identified the most accessible pathway for the rival avenues for direct and reductive hydroamination. The mechanistic picture derived from smooth energy profiles obtained by employing a reliable computational protocol applied to a realistic catalyst model conforms to all available experimental data. The crucial vinyl- and alkylcopper intermediates were found to display a distinct chemodivergence in their reactivity towards amine electrophile and alcohol, which ensures the successful formation of α-branched alkylamines together with (*E*)-enamines. On the one hand, the vinylcopper is somewhat preferably approached by the alcohol, thereby rendering the reductive hydroamination avenue favourable in the presence of both amine electrophile and alcohol. In contrast, the greater kinetic demands for protonation *versus* electrophilic amination predicted for the alkylcopper prevents the reductive hydroamination avenue to become non-productive. Electronically modified hydroxylamine esters are found to influence the chemoselectivity in reactivity towards amine electrophile and alcohol achievable for the vinyl- and alkylcopper, thereby offering an opportunity for process improvement.

## Abstract 2

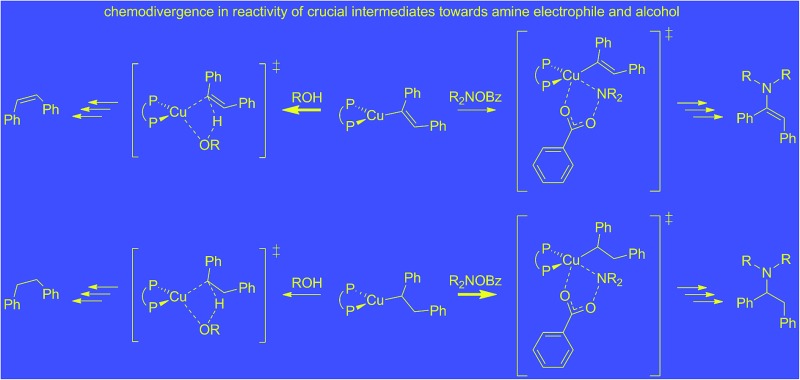
An in-depth computational mechanistic probe of the CuH-mediated hydroamination of internal arylalkynes with amine electrophile and hydrosilane defines the most accessible pathways for rival avenues of direct and reductive hydroamination, from which a general understanding of the factors controlling formal hydroamination catalysis emerges.

## Introduction

**Scheme 1:**
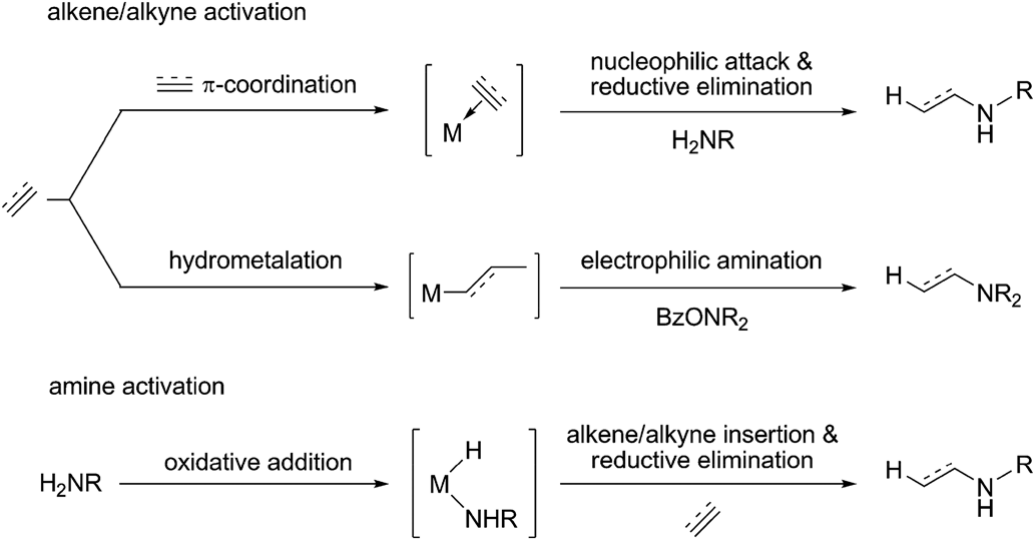
General avenues for substrate activation pursued in late transition metal-mediated hydroamination.

Hydroamination (HA) is defined as the addition of an N–H bond of amines across an unsaturated carbon–carbon linkage. It represents one of the conceptually simplest and atom-economical approaches for the generation of valuable nitrogen building blocks and is thus a topic of considerable interest in both academia and industry.^1^ Although thermodynamically feasible the straightforward reaction is hampered by substantial kinetic demands, which makes the involvement of a catalyst almost indispensable. A variety of approaches toward catalytic hydroamination has been advanced over the years, including Brønsted acid-catalysed,^2^ base-catalysed,^3^ radical-mediated,^4^ metal-catalysed^1^ and pericyclic^5^ transformations. Among the various catalyst systems covering almost the entire periodic table developed over past decades, catalysts based on late d-block metals offer several advantages,^6^ such as greater stability toward air and moisture together with high compatibility for functional groups. Various viable mechanistic avenues have emerged for late d-block metal catalysis over recent years, which can broadly be described as follows: nucleophilic attack of an amine at a metal bound unsaturated C–C linkage,^7^ N–H bond activation to be followed by the insertion of a C–C unsaturation into the metal–NR_2_ linkage,^8^ nucleophilic attack of a metal amido species at an activated unsaturated C–C linkage,^9^ and amine coordination with subsequent proton transfer onto an activated C–C unsaturation.^10^ Although of the considerable advances achieved thus far the utilisation of these protocols still remains limited and a general approach for regio- and stereoselective catalytic hydroamination of a broad range of substrate classes is still elusive (Scheme 1).

**Scheme 2:**
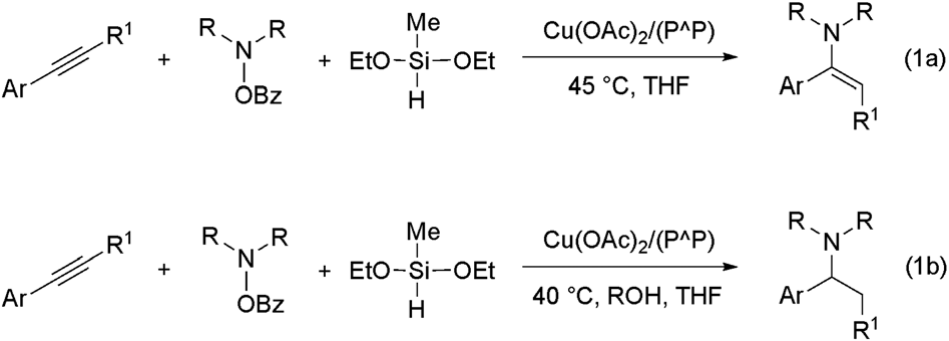
Copper(i) hydride mediated HA of internal arylalkynes with an hydroxylamine ester and a hydrosilane with (**1b**) or without (**1a**) a proton donor present ({P^P} = 4,5-bis(diphenylphosphino)-9,9-dimethylxanthene).

In 2013, the groups of Miura/Hirano^11*a*^ and Buchwald^11*b*^ reported independently on a mechanistically novel approach for formal hydroamination employing upon a regiocontrolled hydrometalation of an olefin functionality and subsequent C–N bond formation through Umpolung electrophilic amination. The intermolecular reaction of vinylarenes with a hydride delivered from a silane and an electrophilic amino group from a hydroxylamine derivative, rather than an amine N–H bond, in the presence of a diphosphine copper compound gives rise to amines in excellent yields and enantio-/regioselectivities under mild conditions.^12^ This approach has been recently successfully extended to alkyne substrates.^13^ Buchwald and co-workers disclosed that internal arylalkynes react with electrophilic amination reagents in the presence of the catalytically competent (Xantphos)Cu^I^ hydride (Xantphos ≡ {P^P} ≡ 4,5-bis(diphenylphosphino)-9,9-dimethylxanthene) in THF at slightly elevated temperature to afford from the same reactants, under different sets of conditions, either (*E*)-enamines or α-branched alkylamines in high chemo-/regio-/stereoselectivities.^13*b*^ In the absence of a proton donor, internal arylalkynes are selectively transformed into the respective (*E*)-enamines (Scheme 2a). However, when a protic alcohol additive is included, internal arylalkynes (and also aryl-/alkylacetylenes) are converted into α-branched (and linear) alkylamines (Scheme 2b).^14^

**Scheme 3:**
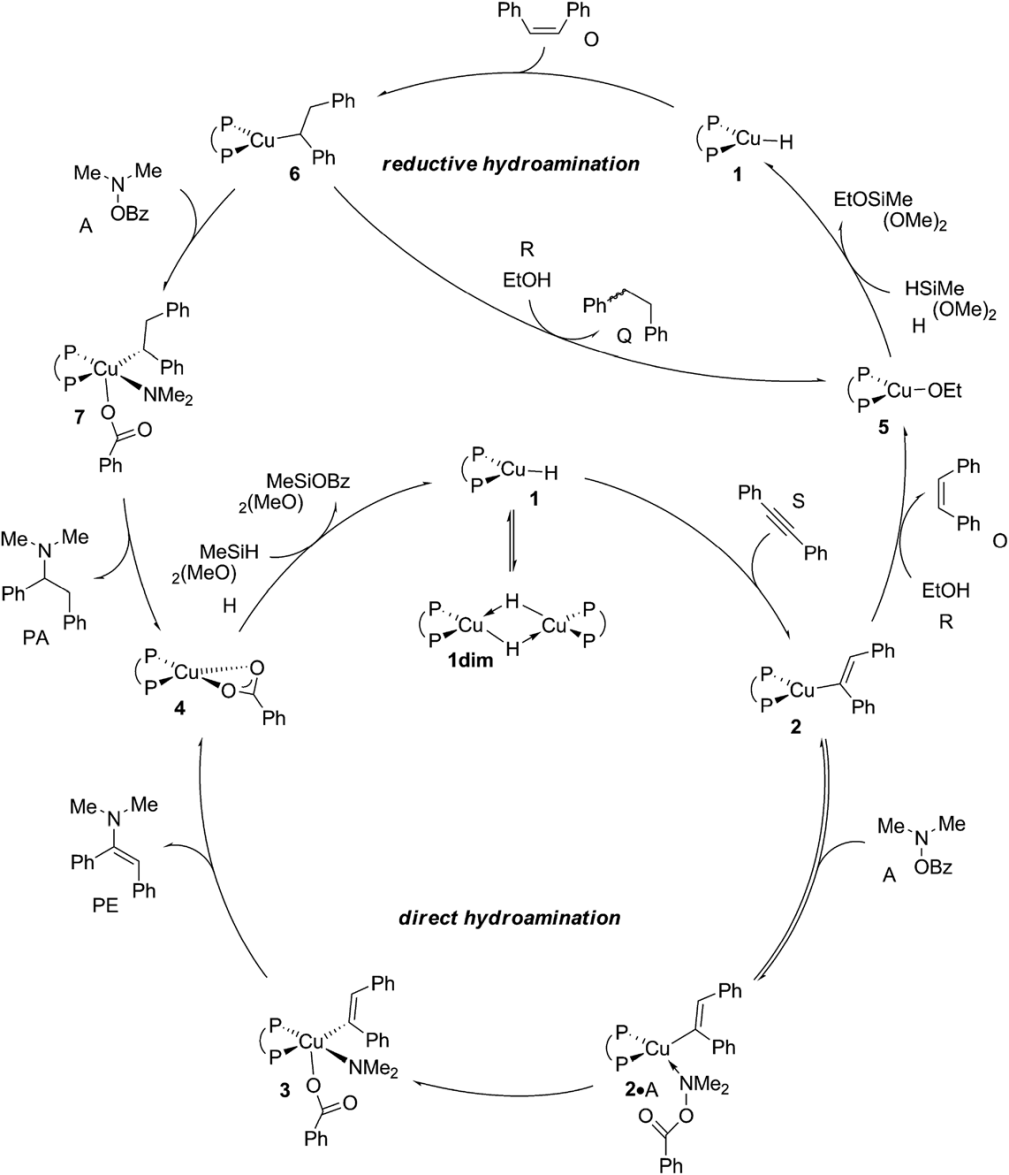
Plausible mechanistic pathways for CuH-catalysed hydroamination of internal arylalkynes with hydroxylamine esters and with or without a protic alcohol additive present, affording α-branched alkylamine (PA) or (*E*)-enamine (PE) products, respectively, exemplified for diphosphine [{P^P}Cu(H)] copper(i) hydride **1** as the catalytically competent compound and 1,2-diphenylacetylene (S), *O*-benzoyl-*N*,*N*-dimethyl-hydroxylamine (A), dimethoxymethylsilane (H) and ethanol (R) reactants, ({P^P} = 4,5-bis(diphenylphosphino)-9,9-dimethylxanthene).

According to plausible mechanistic pathways outlined in Scheme 3, which are based upon seminal work of Lipshutz, Tsuji, Lalic and others,^15^ alkyne (S) insertion into the Cu–H linkage at **1** affords {P^P}Cu^I^ vinyl **2**, which could open two separate avenues. In the absence of a proton donor, the direct hydroamination avenue is traversed, where **2** couples with the hydroxylamine electrophile (A) to generate (*E*)-enamine (PE) and {P^P}Cu^I^ benzoate **4**. This transformation can proceed through various mechanistic pathways,^16^ its precise details remain largely elusive thus far. If, however, an alcohol additive (R) is present, the avenue for reductive hydroamination would become available, whereby protonation occurs at **2** to deliver {P^P}Cu^I^ alkoxide **5** and *cis*-alkene (O). Regeneration of the {P^P}Cu^I^ hydride **1** through transmetalation with hydrosilane (H) and subsequent *cis*-alkene (O) insertion generates {P^P}Cu alkyl **6**. Coupling with the amine electrophile (A) leads to alkylamine (PA) and {P^P}Cu^I^ benzoate **4**. Transmetalation of **4** with hydrosilane regenerates the catalytically competent {P^P}Cu^I^ hydride for another catalyst turnover. As it becomes evident from Scheme 3, the successful generation of alkylamine PA necessitates {P^P}Cu^I^ vinyl **2** and alkyl **6** to react in a highly chemoselective manner. On the one hand, {P^P}Cu^I^ vinyl **2** needs to be selectively intercepted by the alcohol in the presence of the hydroxylamine ester to furnish transient *cis*-alkene and **6**. Conversely, {P^P}Cu^I^ alkyl **6** needs to be selectively approached by the amine electrophile and not by the protic additive to ultimately furnish alkylamine PA.

Further advances in the conceptually novel CuH-mediated hydroamination of alkynes undoubtedly rely on the delineation of the operative mechanism together with a detailed understanding of factors governing the propensity of {P^P}Cu^I^ vinyl **2** and alkyl **6** to engage either with amine electrophile or alcohol. Computational methods are well suited to exploring reaction mechanisms and unveiling crucial catalytic structure–performance relationships, providing a detailed atomistic picture of the structures of key intermediates and transition states that can form the basis for designing improved catalysts. In light of the fact that, despite the thorough account reported,^13*b*^ intimate details of mechanistic intricacies remain largely elusive thus far, an advanced, reliable and well benchmarked computational protocol has been employed to derive a microscopically correct view for the formal HA of arylalkynes. The invaluable insights such approach offers has been demonstrated by a recent computational examination of the CuH-catalysed HA of vinylarenes.^17^ Based upon our previous experience, as well as using a carefully chosen structural model, which mimics crucial aspects of the catalyst system as closely as possible, together with an advanced computational method, paying attention to aspects such as solvation and binding of excess substrate/product molecules at the metal centre, it is necessary to use careful screening of conformational space to ensure that the energy profiles obtained portray the catalyst's behaviour appropriately. Furthermore, unwanted side steps, which are likely compromising catalyst performance, should not be left unconsidered. Given that one of the present study's aims is at delineating the precise details of the operating mechanism, but leaving all aspects related to stereocontrol out of consideration, for now, a copper catalyst featuring an achiral diphosphine ligand appears most suitable. Hence, the catalytically competent Xantphos-ligated Cu^I^ hydride and 1,2-diphenylacetylene (S) alkyne together with prototype hydroxylamine ester and silane, which has been studied experimentally,^13*b*^ were selected for the present study. Various mechanistic pathways for rival avenues for direct and reductive HA of alkyne S with *O*-benzoyl-*N*,*N*-dimethyl-hydroxylamine (A), ethanol (R) and dimethoxymethylsilane (H) by a (Xantphos)Cu^I^ hydride, with or without ethanol (R) as protic alcohol additive present. For the sole purpose of computational efficiency, *O*-benzoyl,*N*,*N*-dibenzyl-hydroxylamine and diethoxymethylsilane used in experiment^13*b*^ were replaced by A and H, respectively. Otherwise the full experimental structures were employed herein.

The computational methodology employed (reliable state-of-the-art hybrid *meta*-GGA PW6B95-D3 functional in conjunction with flexible basis sets of def2-TZVP quality and a sound treatment of bulk solvent effects; see the ESI for more details†) simulated authentic reaction conditions adequately and the mechanistic analysis is based on Gibbs free-energy profiles. The validity of the computational protocol adopted to reliably map the energy landscape of CuH-mediated formal hydroamination processes has been substantiated before,^17^ and this has allowed mechanistic conclusions with substantial predictive value to be drawn. Whilst the presentation herein is restricted exclusively on the most accessible pathways, a complete account of all scrutinised species is given in the ESI.†

The comprehensive mechanistic examination presented herein enables us to put the mechanistic hypotheses advanced previously on a much firmer footing by defining the sequence of steps (and their respective minimum-energy pathways) traversed along rival avenues for direct and reductive hydroamination. Moreover, the present study provides a detailed understanding of factors governing the generation of (*E*)-enamine and/or alkylamine products and unveils a more fundamental insight into catalytic structure–performance relationships together with guidelines for rational process improvement.

## Results and discussion

The present computational mechanistic study is divided into several parts. It starts with a careful exploration of the chemodivergent avenues for direct and reductive HA catalysis. A further part examines alternative mechanistic avenues for (1) reduction of the (*E*)-enamine, (2) generation of diamination products and (3) non-productive consumption of either the protic additive or the amine electrophile by the {P^P}Cu^I^ hydride. It leads to define the most accessible pathway towards the formation of (*E*)-enamine and alkylamine products. A final part explores to what extent an electronically modified amination reagent effects the propensity of {P^P}Cu^I^ vinyl **2** and alkyl **6** to engage with either amine electrophile or alcohol. In what follows, all free energies are quoted relative to the prevalent [{P^P}Cu^I^(H)]_2_ dimer **1dim** of the catalytically competent (Xantphos)Cu^I^ hydride plus appropriate reactants (S, A, H, R, O), enamine/amine products (PE, PA) and THF (T) unless noted otherwise.

### A Mechanistic avenue for direct HA to convert arylalkynes into (*E*)-enamines

#### A.1 {P^P}Cu^I^ hydride compound

**Fig. 1:**
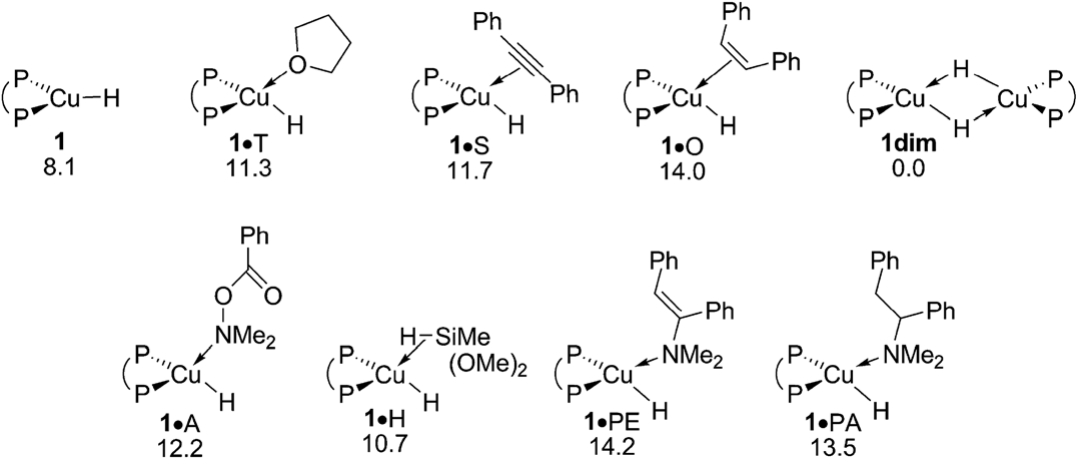
Various forms of the catalytically relevant {P^P}Cu^I^ hydride compound **1**.^19,20^ Free energies are given in kcal mol^–1^ relative to {½**1dim** + reactants}.

The {P^P}Cu^I^ hydride plays a pivotal role in the catalytic transformation (Scheme 3) – its precise nature, however, remains largely elusive. On the one hand, copper hydrides are known for their aptitude to form higher ordered aggregates,^18^ whilst reactant, product and solvent molecules can otherwise bind at copper to stabilise the copper hydride monomer **1**. A dimer **1dim** could be located, which is found 8.1 kcal mol^–1^ more stable relative to **1** in free energy (Fig. 1 and S1†). The association of substrate, product or solvent at **1** is seen to be restricted to a single molecule only. Not only were all the attempts to locate species with two or more associated molecules unsuccessful, suitable candidate structures relax almost instantaneously into mono-adducted species upon dissociation of excess molecules. In contrast to the noticeable thermodynamic force predicted for dimer formation, a single molecule binds at **1** with a moderate enthalpy only, which places all the various adducted species higher in free energy relative to the respective separated fragments, owing to the associated entropy cost. Hydroxylamine ester A, alkyne S, hydrosilane H reactants, THF T, and also enamine PE and amine PA products show a broadly comparable aptitude to bind at copper (Fig. 1 and S1†). To summarise, the catalytically competent {P^P}Cu^I^ hydride favours its dimer **1dim** over all the substrate adducted species, all of which are expected less appreciably populated, due to a thermodynamic gap of at least 11 kcal mol^–1^. However, **1** and not **1dim**, both of which are in mobile equilibrium,^19^ is the direct precursor for hydrocupration and also non-productive consumption of amine electrophile and alcohol (see below). Hence, the Cu^I^ hydride monomer **1** is likely representing the catalytically competent species, which is supported by a spectroscopic mechanistic study of alkene HA by a similar DBTM-SEGPHOS-ligated Cu^I^ catalyst^21^ together with a recent computational study.^17^

#### A.2 Alkyne hydrocupration

Starting from the {P^P}Cu^I^ hydride catalysis is initiated by the hydrocupration of the symmetrical diaryl-substituted alkyne S to afford {P^P}Cu^I^ vinyl **2**, which could then branch into the separate avenues for direct and reductive HA, respectively (Scheme 3). Commencing from alkyne adduct **1·**S the *syn*-selective migratory C≡C bond insertion into the Cu–H σ-bond delivers a single isomer of **2**. In a more general context, electronic factors appear to dominate the regioselective outcome of the hydrocupration step for arylalkynes. Previous computational studies^17,22^ on hydrocupration of vinylarenes by diphosphine-ligated copper hydrides have shown that the stability of both the polarised transition-state (TS) structure and the copper alkyl formed is markedly influenced by the opportunity for delocalisation of electron density offered by the π-electron-withdrawing arene functionality adjacent to copper. It renders the Markovnikov hydrocupration energetically prevalent over its anti-Markovnikov alternative, owing to the absence of such stabilising interaction for the latter.

**Fig. 2:**
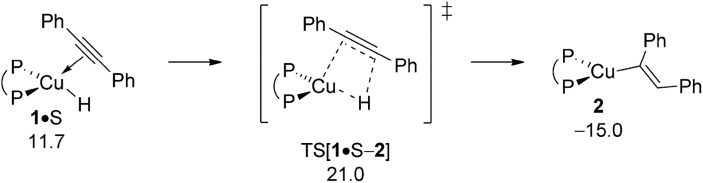
Alkyne insertion into the Cu–H linkage at alkyne adduct **1·**S of the {P^P}Cu^I^ hydride compound.^19,20^ Free energies are given in kcal mol^–1^ relative to {½**1dim** + reactants}.

Hydrocupration proceeds with the association of S onto the copper centre to give weakly π-bound intermediate **1·**S, sees the initial rotation of the alkyne to achieve a near-planar four-centre planar TS structure describing migratory *syn*-insertion of the alkyne C≡C linkage into the electron-rich Cu–H bond. The located TS[**1·**S–**2**] represents a relatively early point on the insertion coordinate as evidenced by the short (1.28 Å) carbon–carbon and long (1.72 Å) C–H distances (Fig. S2†) and is directly connected to {P^P}Cu^I^ vinyl **2** featuring a *cis*-alkenyl moiety. As it was noted in previous studies^17,22^ available reactant (A, S), enamine product (PE) or THF solvent (T) molecules are not likely facilitating the process. All our efforts locating encounter complex, product or TS structures featuring a stable association of a spectator molecule remained unsuccessful.^23^ Overall, migratory alkyne insertion is predicted to be kinetically viable (Δ*G* ^‡^ = 21.0 kcal mol^–1^ relative to {½**1dim** + S}) driven by a rather substantial thermodynamic force (Fig. 2), hence irreversible.

#### A.3 Electrophilic amination at {P^P}Cu^I^ vinyl

Pursuing along the avenue for direct HA the hydroxylamine electrophile approaches the vinylcopper nucleophile to furnish (*E*)-enamine PE and {P^P}Cu^I^ benzoate **4** (Scheme 3). Among the multiple mechanistic scenarios imaginable,^16^ three plausible pathways for Umpolung electrophilic amination have been characterised commencing from adducts **2·**A. A first pathway sees the S_N_2-type displacement of the amine electrophile's benzoate leaving group *via* a classical TS structure to afford transient {P^P}Cu^III^ intermediate **3**, which likely undergoes C–N bond-forming reductive elimination thereafter to deliver PE together with {P^P}Cu^I^ benzoate **4**. Alternatively, the S_N_2-type displacement can involve a multi-centre TS structure. The **2·**A → **3** oxidative addition across the N–O linkage has been examined as the third scenario.

**Fig. 3:**
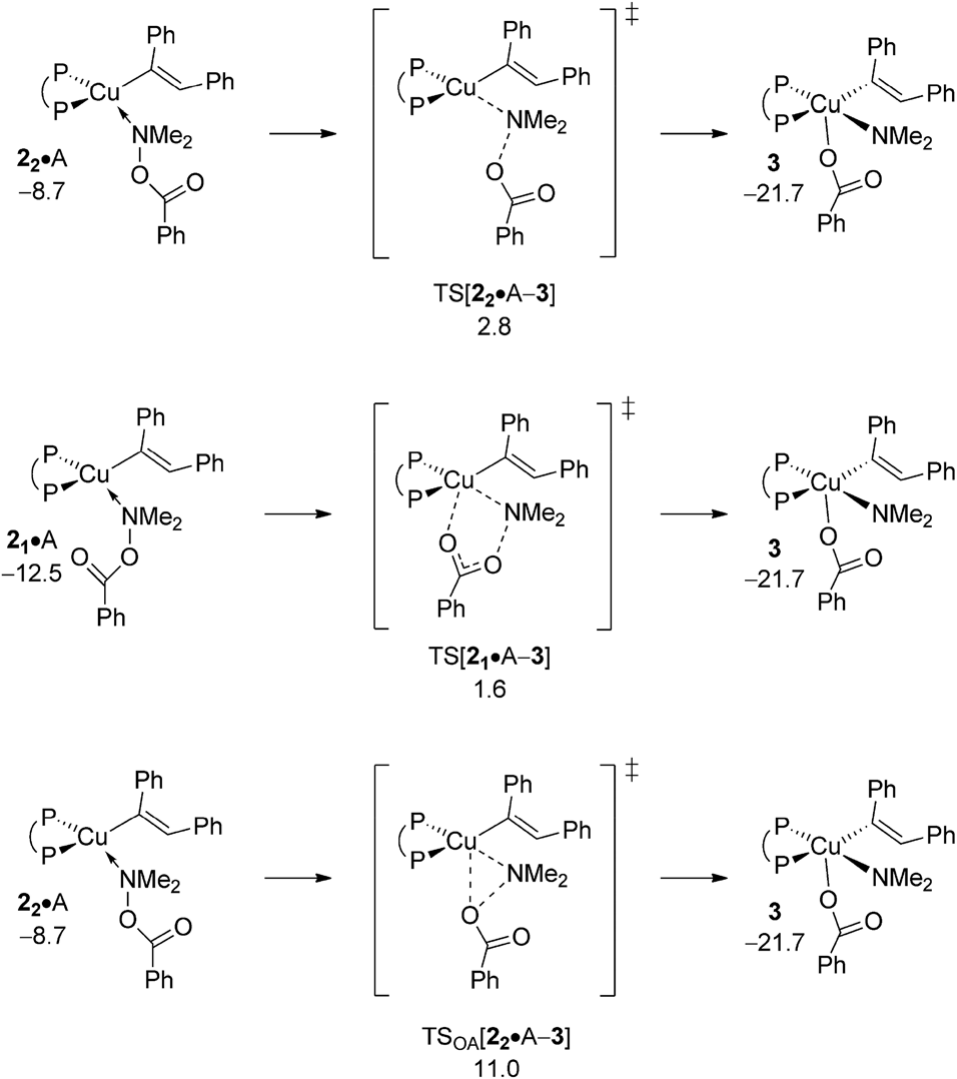
Most accessible pathways for cleavage of the hydroxylamine ester's N–O linkage at amine adduct **2·**A of the {P^P}Cu^I^ vinyl intermediate through alternative mechanistic pathways.^19,20^ Free energies are given in kcal mol^–1^ relative to {½**1dim** + reactants}.

The energetics of the most accessible pathways for the cleavage of the hydroxylamine ester's N–O linkage are collated in Fig. 3, with structural aspects of key species involved can be found in the ESI (Fig. S3–S8†). The profiles start with a pair of N-bound amine electrophile adducts, **2_1_·**A/**2_2_·**A, as the most stable adduct forms of **2·**A, with η^1^-O-bound forms are found somewhat higher in free energy. A first pathway starts from **2_2_·**A, which has the unbound carboxylate pointing at the copper centre, traverses multicentre TS[**2_1_·**A–**3**] featuring crucial distances of 2.07 Å (emerging Cu···N_amido_) and 1.72 Å (vanishing N···O bonds) together with a contact between copper and oxygen (2.72 Å) that begins to form (see Fig. S3†), to decay directly into transient {P^P}Cu^III^ benzoate amido vinyl intermediate **3** thereafter. Another variant of the S_N_2-type N–O bond cleavage has been characterised. It can be seen as the more classical pathway for S_N_2 displacement of the benzoate leaving group taking place *via* TS[**2_2_·**A–**3**], which shows similar metrics regarding emerging Cu···N_amido_ and vanishing N···O bonds (see Fig. S5†), but with **2_2_·**A and TS[**2_2_·**A–**3**] featuring an carboxylate moiety that crucially points away from the copper centre. The two S_N_2-type pathways furnish eventually the same isomer of **3**; it is the emerging Cu···O contact in the multicentre TS that renders **2_1_·**A → **3** more accessible kinetically than **2_2_·**A → **3**, which is devoid of such stabilising interaction (see Fig. 3). The third examined scenario, *viz.* oxidative addition across the N–O linkage, evolves commencing from **2_2_·**A through three-centre TS_OA_[**2_2_·**A–**3**] to afford **3** directly. Notably, in some contrast to the located S_N_2-type TS structures TS_OA_[**2_2_·**A–**3**] occurs relatively late on the **2·**A → **3** coordinate as evidenced by the rather elongated (2.43 Å) N–O and short (2.58/1.89 Å) Cu–O/Cu–N_amido_ distances (Fig. S7†). It becomes clear from the profiles collated in Fig. 3 that in the presence of the kinetically dominant S_N_2-type cleavage of the electrophile's N–O bond by the alkenylcopper nucleophile through a multicentre TS structure the substantially more demanding oxidative addition (ΔΔ*G* ^‡^ = 9.4 kcal mol^–1^, relative to TS[**2_1_·**A–**3**]) is energetically prohibitive and hence unlikely be traversable.

**Fig. 4:**
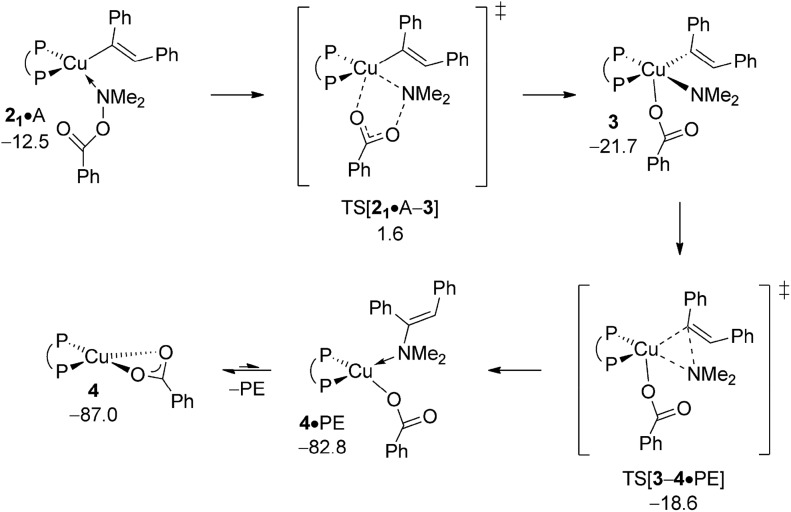
Most accessible route for generation of the enamine product (avenue of direct HA) through S_N_2 displacement of the benzoate leaving group at **2·**A to involve a multicentre TS structure, to be followed by N–C bond-forming reductive elimination at **3**.^19,20^ Free energies are given in kcal mol^–1^ relative to {½**1dim** + reactants}.

The {P^P}Cu^III^ **3** appears to be a metastable, short-lived intermediate, which readily undergoes C–N bond-forming reductive elimination to deliver PE together with {P^P}Cu^I^ benzoate **4** (Fig. 4). The geometry of **3** is set up favourably such that the process trajectory does not see a major metric restructuring until TS[**3–4·**PE] is crossed. The TS appears relatively early with the process progressing thereafter to yield enamine adducted {P^P}Cu^I^ benzoate **4·**PE, from which PE is readily released thereafter.^19^ Consistent with this picture is the rather marginal lengthening of both the Cu–N_amido_ and Cu–C_vinyl_ distances (see Fig. S9†) to 1.93 and 2.00 Å, respectively, in TS[**3–4·**PE] (*cf.* 1.93 and 1.96 Å in **3**), whilst the emerging Cu···N bond (2.40 Å) is barely pre-formed in the TS structure (*cf.* 1.40 Å in free PE). As a consequence, reductive elimination is predicted to be remarkably facile (Δ*G* ^‡^ = 3.1 kcal mol^–1^ relative to **3**) and strongly downhill thermodynamically. All in all, electrophilic amination of the vinylcopper nucleophile by A preferably starts with the S_N_2-type displacement of the benzoate leaving group evolving through a multicentre TS structure, which features a defining barrier of 16.6 kcal mol^–1^ (relative to {**2** + A}) with the thus generated short-lived {P^P}Cu^III^ intermediate **3** undergoing strongly downhill and highly facile reductive elimination thereafter to deliver enamine PE together with **4**. It is worth pointing out that such scenario for Umpolung electrophilic amination was noted in our previous study on formal HA of styrene by a comparable copper-based catalyst system.^17^

#### A.4 Transmetalation at {P^P}Cu^I^ benzoate

**Fig. 5:**
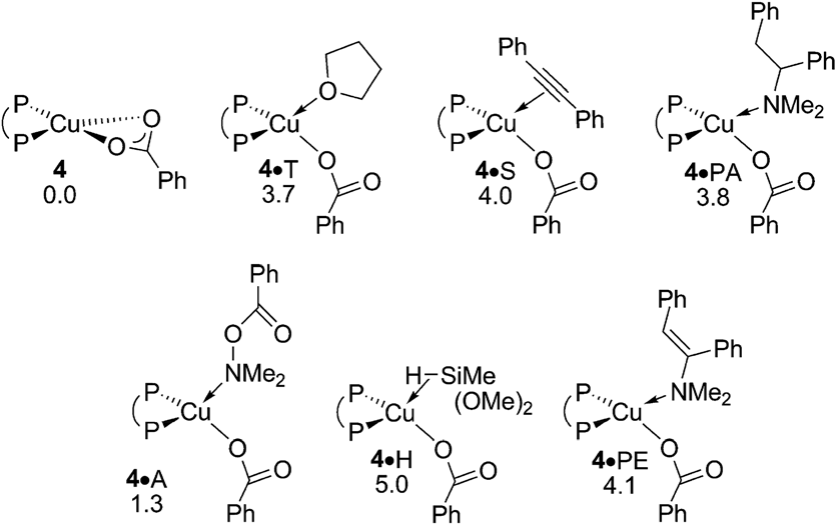
Various forms of the {P^P}Cu^I^ benzoate compound **4**.^19^ Free energies are given in kcal mol^–1^ relative to {**4** + reactants}.

After the electrophilic amination of **2** is successfully accomplished the catalytic cycle is completed by the transformation of the thus generated stable {P^P}Cu^I^ benzoate **4** back into the catalytically competent {P^P}Cu^I^ hydride through transmetalation with hydrosilane H – a step shared among the chemodivergent avenues for direct and reductive HA catalysis (Scheme 3). It is worth mentioning that transmetalation of **4** is likely turnover limiting in formal HA of vinylarenes with hydroxylamine esters and hydrosilanes by diphosphine copper compounds, as revealed by recent complementary experimental^21^ and computational^17^ approaches. It thus triggered our interest in the precise nature of the {P^P}Cu^I^ benzoate. Association of available substrate, product or THF molecules onto **4** featuring a κ^2^-O copper benzoate ligation is seen to be accompanied by a swift change into a κ^1^-O coordination mode (see Fig. S11†). A single molecule only can bind at copper^23^ with a rather modest enthalpy of similar amount for all the probed molecules. It places all the located single molecule adducts above the lowest energy non-adducted form **4** of the {P^P}Cu^I^ benzoate in free energy (Fig. 5).

**Fig. 6:**
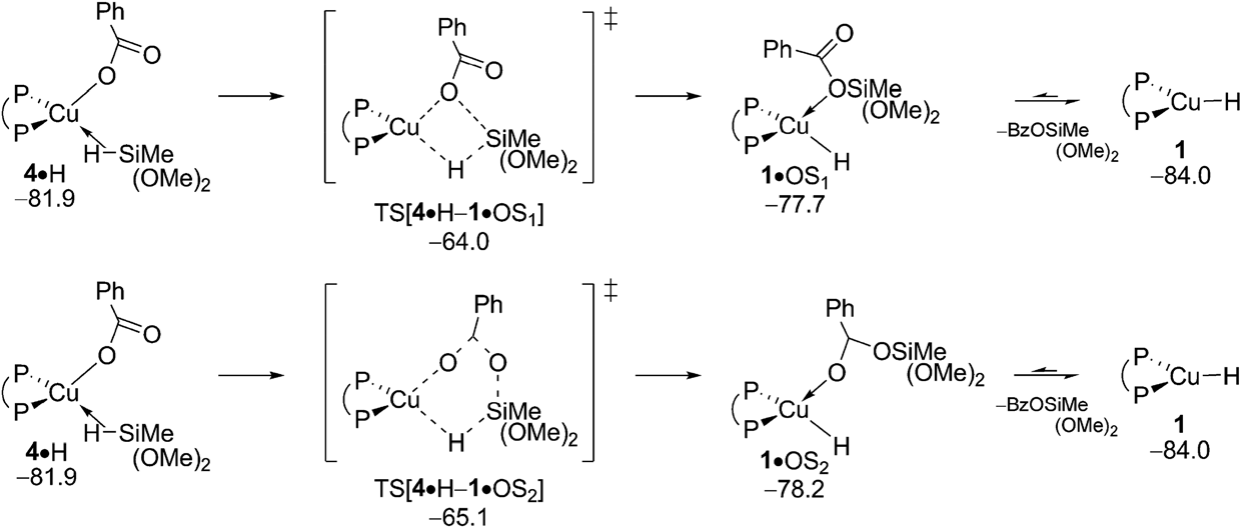
Transmetalation of the {P^P}Cu^I^ benzoate **4** with hydrosilane H.^19,20^ Free energies are given in kcal mol^–1^ relative to {½**1dim** + reactants}.

The benzoate group can be approached by an initially loosely associated hydrosilane (**4·**H) in different ways. Not only can the silicon engage with the carboxylate oxygen directly bound to copper, but Si–O bond formation can also involve the unbound carboxylate oxygen centre. These two pathways are characterised in Fig. 6 and S12.† The located four-centre TS[**4·**H–**1·**OS_1_], which describes the first scenario, decays into silicate-adduct **1·**OS_1_ to become readily converted thereafter into {P^P}Cu^I^ hydride **1** upon facile release of (OMe)_2_MeSiOBz.^19^ The thermodynamically identical silyl transfer onto the unbound carboxylate oxygen centre evolves through six-centre TS[**4·**H–**1·**OS_2_]. DFT predicts silyl transfer through six-centre TS[**4·**H–**1·**OS_2_] to be somewhat faster than proceeding through four-centre TS[**4·**H–**1·**OS_1_]. Its barrier of 21.9 kcal mol^–1^ (Δ*G* ^‡^ relative to {**4** + H}) characterises transmetalation at {P^P}Cu^I^ benzoate to be kinetically viable under the adapted reaction conditions (Fig. 6).

### B Mechanistic avenue for reductive HA to convert arylalkynes into α-branched alkylamines

#### B.1 Protonation of the {P^P}Cu^I^ vinyl

**Fig. 7:**
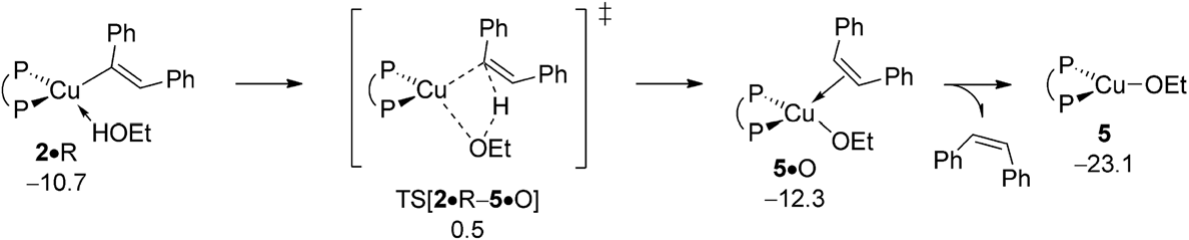
Protonation of the {P^P}Cu^I^ vinyl intermediate **2** by EtOH.^19,20^ Free energies are given in kcal mol^–1^ relative to {½**1dim** + reactants}.

The first step along the rival avenue for reductive HA is protonation of vinylcopper **2** by alcohol R. It sees the initial facile,^19^ but somewhat uphill association of a single alcohol molecule to give adduct **2·**R featuring an only loosely H-bonded ethanol molecule. Intramolecular hydrogen transfer evolves through a metathesis-type TS structure TS[**2·**R–**5·**O] that describes the cleavage of an already suitably polarised O–H bond with concurrent C–H bond formation (see Fig. S13†). It leads first to *cis*-olefin adducted {P^P}Cu^I^ ethoxide **5·**O, but rearranges thereafter swiftly into **5** upon a thermodynamically downhill easy release of *cis*-diphenylethylene O. The rather moderate barrier (Δ*G* ^‡^ = 15.5 kcal mol^–1^ relative to {**2** + R}) associated with the protonation of **2** by ethanol, together with its net exergonicity (Δ*G* = –8.1 kcal mol^–1^ relative to {**2** + R}) upon the release of O (Fig. 7) is indicative that vinylcopper **2** not only is likely undergoing electrophilic amination (see above) but also can engage with an alcohol additive, if available, at affordable energy costs. Hence, the avenue for reductive HA would be accessible, which is elaborated upon further below in more detail. The participation of additional spectator reactant, product or solvent molecules to facilitate the process has been probed explicitly, but found to be ineffective.^23^

#### B.2 Transmetalation at {P^P}Cu^I^ alkoxide

**Fig. 8:**
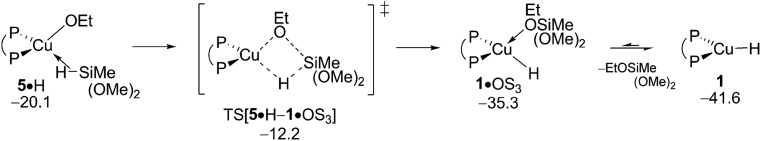
Transmetalation of the {P^P}Cu^I^ alkoxide **5** with hydrosilane H.^19,20^ Free energies are given in kcal mol^–1^ relative to {½**1dim** + reactants}.

σ-Bond metathesis with hydrosilane H transforms {P^P}Cu^I^ ethoxide **5** back into **1**. Commencing from a weakly bound silane adduct **5·**H the quasi-planar four-centre TS[**5·**H–**1·**OS_3_] is traversed (see Fig. S14†), which decays thereafter into {P^P}Cu^I^ hydride **1** through facile liberation of Me_3_SiOEt. Fig. 8 reveals an affordable activation energy (Δ*G* ^‡^ = 10.9 kcal mol^–1^ relative to {**5** + H}) for the transformation of **5** back into the catalytically competent {P^P}Cu^I^ hydride **1**, which is moreover driven by a thermodynamic force of considerable amount.

#### B.3 Alkene hydrocupration

**Fig. 9:**
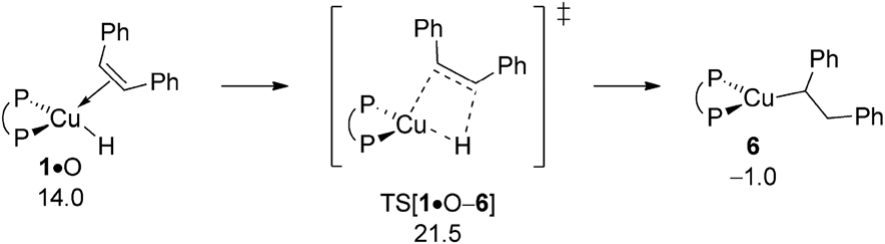
*cis*-Alkene insertion into the Cu–H linkage at olefin adduct **1·**O of the {P^P}Cu^I^ hydride compound.^19,20^ Free energies are given in kcal mol^–1^ relative to {½**1dim** + reactants}.

With the catalytically competent {P^P}Cu^I^ hydride **1** rebuilt, *cis*-alkene O insertion generates α-aryl branched alkylcopper **6**. Fig. 9 summarises the free-energy profile assessed, whilst structural aspects of key species involved can be found in the ESI (see Fig. S15†). Hydrometalation of alkene O and alkyne S taking place through *syn*-insertion of the respective unsaturated carbon–carbon linkage into the electron-rich Cu–H bond through simultaneous C–H/Cu–C bond formation, share similar structural aspects. The moderate elongation of the unsaturated C–C bond (1.44 Å in TS[**1·**O–**6**] relative 1.40 Å in **1·**O and 1.28 Å in TS[**1·**S–**2**] relative 1.26 Å in **1·**S, respectively) together with a barely established emerging C···H bond (1.68 Å in TS[**1·**O–**6**] and 1.72 Å in TS[**1·**S–**2**], respectively) are indicative that the four-membered TS structures appear at an early stage of the migratory insertion process. These structural features are paralleled in general aspects of the derived energy profiles. Hydrocupration is seen to be kinetically viable and downhill, hence likely be irreversible, in either case. Notably, migratory insertion of *cis*-alkene O (Δ*G* ^‡^/Δ*G* = 21.5/–1.0 kcal mol^–1^ relative to {½**1dim** + O}) not only is predicted somewhat less rapid than hydrocupration of alkyne S (Δ*G* ^‡^/Δ*G* = 21.0/–15.0 kcal mol^–1^ relative to {½**1dim** + S}; see Fig. 2) but also disfavoured on thermodynamic grounds, owing to a much weaker thermodynamic force.

#### B.4 Electrophilic amination at {P^P}Cu^I^ alkyl

**Fig. 10:**
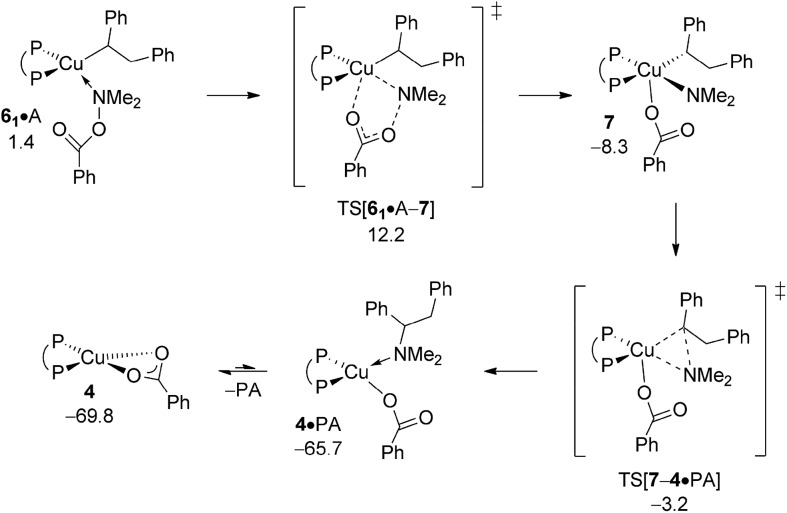
Most accessible route for generation of the α-branched alkylamine product (avenue of reductive HA) through S_N_2 displacement of the benzoate leaving group at **6·**A involving a multicentre TS structure, to be followed by N–C bond-forming reductive elimination at **7**.^19,20^ Free energies are given in kcal mol^–1^ relative to {½**1dim** + reactants}.

The aptitude of nascent **6** to engage with amine electrophile A will be studied next. As with our above examination on vinylcopper **2** three alternative routes for N–O bond rupture together with subsequent N–C bond-forming reductive elimination from the thus formed transient {P^P}Cu^III^ intermediate **7** have been studied. The assessed profiles for the S_N_2-type N–O bond cleavage evolving through multicentre or classical TS structures and also for oxidative addition across the N–O linkage can be found in the ESI† together with structural aspects of key species involved (see Fig. S16–S24†). It should be noted that the key species involved along the various pathways for electrophilic amination at **6** and **2** share similar structural aspects. Here we wish to focus exclusively on the most accessible pathway derived from DFT shown in Fig. 10. It starts from adduct **6_2_·**A with the displacement of the leaving group through S_N_2-type multicentre TS[**6_1_·**A–**7**] featuring an already established copper···carboxylate contact. As such stabilising interaction is missing here, the alternative S_N_2-type TS[**6_2_·**A–**7**] is found at a higher free energy. Moreover, oxidative addition across the N–O linkage has a substantially higher barrier to overcome and can thus safely be discarded as viable mechanistic route. The thus generated {P^P}Cu^III^ benzoate amido alkyl **7** likely transforms almost immediately into α-branched alkylamine PA and **4** through low-barrier reductive elimination. The smooth profile in Fig. 10 reveals that electrophilic amination of alkylcopper **6** by A is kinetically viable and driven by a strong thermodynamic force as amination of vinylcopper **2** is (see Section A.3), with the S_N_2-type displacement of the benzoate leaving group evolving through a multicentre TS structure defines the overall kinetic demands. The catalytic cycle would be completed by transmetalation of **4** with hydrosilane H (see Section A.4).

#### B.5 Protonation of {P^P}Cu^I^ alkyl

**Fig. 11:**
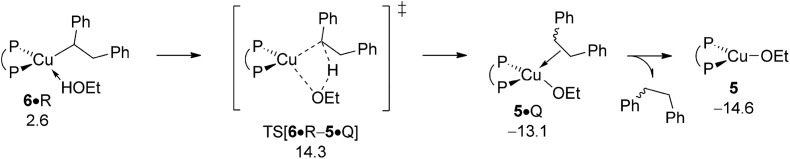
Protonation of the {P^P}Cu^I^ alkyl intermediate **6** by EtOH.^19,20^ Free energies are given in kcal mol^–1^ relative to {½**1dim** + reactants}.

Apart from engaging with amine electrophile alkylcopper **6** could be protonated instead by available ethanol R (Scheme 3). The proton transfer starts from a weak H-bound alcohol adduct **6·**R, traverses four-centre TS[**6·**R–**5·**Q] to provide {P^P}Cu ethoxide **5** together with diphenylethane (Q). Of particular note is that TS[**6·**R–**5·**Q] describing the simultaneous cleavage/generation of O–H/C–H bonds features an already severely weakened Cu–C linkage (see Fig. S25†). Having traversed the TS structure along the reaction trajectory the newly built alkane Q initially remains rather loosely associated with the copper alkoxide in **5·**Q, but could easily be either replaced by available reactant (A, S, O) or solvent (T) molecules, or displaced after all.^19^ The protonolytic alkyl displacement at **6** by ethanol is downhill, mainly driven by the enhanced strength of the Cu–O *vs.* Cu–C bond and has modest kinetic demands (Δ*G* ^‡^ = 15.3 kcal mol^–1^ relative to {**6** + R}, Fig. 11). σ-Bond metathesis with hydrosilane H regenerates the catalytically competent {P^P}Cu^I^ hydride **1** from **5** (see Section B.2). DFT predicts a somewhat smaller barrier for alkylcopper **6** to engage with A than with R, indicating the feasibility of electrophilic amination in the presence of the alcohol additive.

### C Alternative mechanistic avenues

Apart from the plausible mechanistic pathways in Scheme 3, several others can be envisaged. In an attempt to assist a broader understanding of the conceptually novel CuH-mediated formal alkyne hydroamination alternative paths for (1) α-branched alkylamine generation through reduction of the (*E*)-enamine, (2) development of diamination side products and (3) non-productive consumption of amine electrophile or alcohol by the {P^P}Cu^I^ hydride have been examined.

#### C.1 Reduction of (*E*)-enamine

**Fig. 12:**
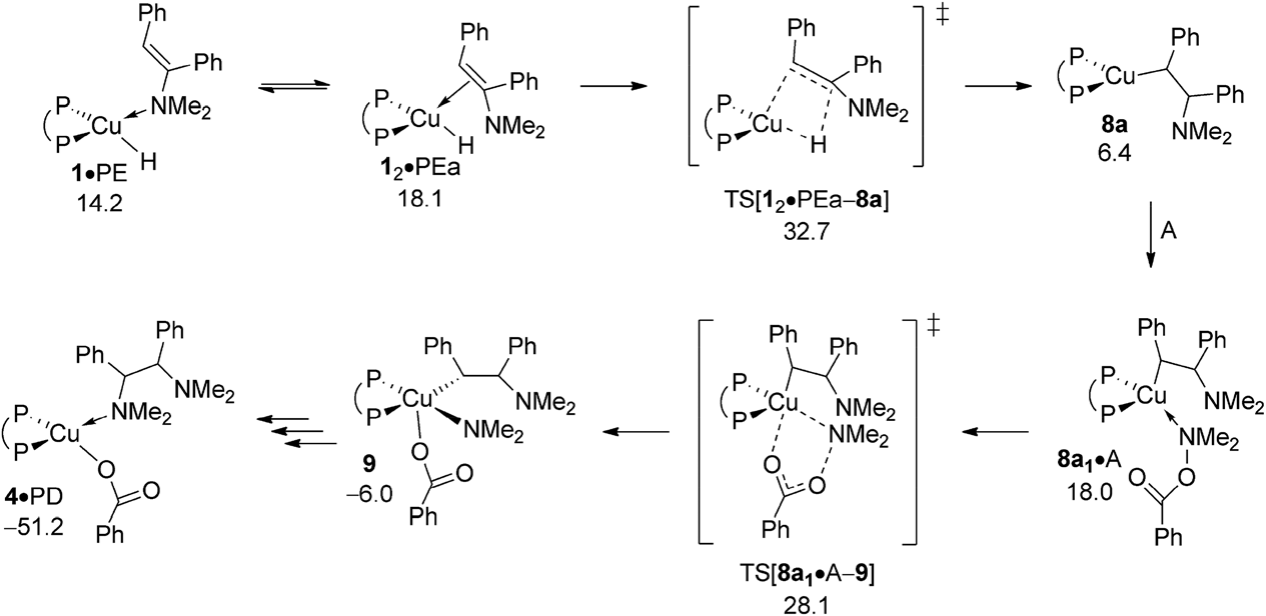
Most accessible route for reduction of the (*E*)-enamine through hydrocupration of PE, S_N_2 displacement of the benzoate leaving group at **8a·**A involving a multicentre TS structure, to be followed by N–C bond-forming reductive elimination as an alternative avenue for generating the α-branched alkylamine product.^19,20^ Free energies are given in kcal mol^–1^ relative to {½**1dim** + reactants}.

Based upon seminal work of Tsuji's^15*b*^ and Lalic's^15*c*^ groups, reduction of enamine PE, comprising of hydrometalation and subsequent protonation of the resulting {P^P}Cu alkylamido by alcohol, could also lead to generate alkylamine PA. The energy profile for regioisomeric pathways for migratory insertion of PE's C=C bond into the Cu–H linkage at **1** (Fig. S27†) together with structural aspects of key species involved (Fig. S26) can be found in the ESI.† The enamine binds preferably *via* its N donor centre at copper to furnish initially **1·**PE, but can readily transform into **1_2_·**PEa or **1_2_·**PEb with the olefinic bond associated at copper, from which {P^P}Cu alkylamido **8a** or **8b** are generated through regioisomeric TS structures for hydrogen transfer onto disubstituted (TS[**1_2_·**PEa–**8a**] affording α-phenyl branched **8a**) and monosubstituted (TS[**1_2_·**PEb–**8b**] affording β-phenyl branched **8b**) olefinic carbon centres, respectively. The enhanced thermodynamic gap assessed for enamine adducts (Δ*G* = 10.0 kcal mol^–1^ for **1_2_·**PEa relative to {**1** + PE}, Fig. 12) when compared to the *cis*-alkene adduct **1·**O (Δ*G* = 5.9 kcal mol^–1^ relative to {**1** + O}, see Fig. 9) is of particular note. Moreover, **1_2_·**PEb is found energetically less favourable by another 6.8 kcal mol^–1^ relative to **1_2_·**PEa (see Fig. S27†), which can mainly be attributed to unfavourable interactions of the disubstituted olefin tail with the {P^P}Cu catalyst backbone. Progressing further on from **1_2_·**PEa and **1_2_·**PEb migratory insertion exhibits an intrinsic barrier (*i.e.* relative to the direct precursor) of remarkably similar amount for traversing the regioisomeric pathways. Nevertheless, the **1_2_·**PEa → **8a** generation of α-phenyl branched {P^P}Cu^I^ alkylamido **8a** prevails on both kinetic and thermodynamic grounds (see Fig. S27†), but it is the greater relative stability of **1_2_·**PEa, which renders **1_2_·**PEa → **8a** overall less demanding kinetically than **1_2_·**PEb → **8b**. All in all, hydrocupration of PE is predicted considerably slower (Δ*G* ^‡^ = 32.7 kcal mol^–1^ relative to {½**1dim** + PE}, Fig. 12) when compared to *cis*-stilbene O (Δ*G* ^‡^ = 21.5 kcal mol^–1^ relative to {½**1dim** + O}; Fig. 9) and also less favourable thermodynamically. In light of its kinetic demands, hydrocupration of (*E*)-enamine PE is rather unlikely to be accessible under the experimentally adopted process regime (Scheme 2).

The subsequent protonolytic cleavage of the Cu–C alkyl linkage at **8a** by ethanol to deliver alkylamine PA with the release of {P^P}Cu^I^ ethoxide **5** is seen to be kinetically affordable (see Fig. S28 and S29†). Notwithstanding of the rapid protonation, it is the slow, if accessible at all, hydrocupration of PE that renders the reduction of enamine non-competitive in the presence of the kinetically less demanding reductive hydroamination path (see Scheme 3 and Section B) for the generation of the α-branched alkylamine product. Competition experiments strongly support this conclusion.^13*b*^ Firstly, when the dibenzyl analogue of PE was subjected to standard HA conditions, only the starting material was recovered, but no alkylamine was formed. Secondly, *cis*-stilbene O was neatly converted into an (*E*)-enamine, when applying standard conditions for reductive HA.

#### C.2 Generation of the diamination product

The propensity of (*E*)-enamine to undergo direct HA is of great interest as far as efficiency of the catalytic protocol is concerned. If PE could compete with alkyne S whilst engaging in direct HA, thereby furnishing diamination product PD, it would compromise catalyst performance severely. Although of the already slow, if not kinetically prohibited after all, hydrocupration of PE (see Section C.1) it is nevertheless informative examining the amination of alkylamidocopper **8a** by electrophile A. Here we only wish to consider the most accessible pathway identified before (Fig. 4 and 10), by focusing on the kinetic demands for the S_N_2-type N–O bond rupture and also the thermodynamics of the overall process (see Fig. S30 and S31†). Given the anticipated low barrier associated with the reductive elimination, its associated TS structure has not been considered in detail. When compared with vinylcopper **2** (Fig. 4) and alkylcopper **6** (Fig. 10) DFT predicts that amination of alkylamidocopper **8a** through the crucial multicentre S_N_2-type TS[**1_2_·**PE–**8a**] is significantly less facile (Δ*G* ^‡^ = 21.7 kcal mol^–1^ relative to {**8a** + A}, see Fig. 12). Taking this together with the already unfavourable kinetic profile for enamine hydrocupration, one can safely conclude that the diamination route is inaccessible and thus catalyst performance can be expected to not be compromised by diamination products generated, consistent with the observed inertness of enamines to engage with the hydroxylamine electrophile under standard HA conditions.^13*b*^

#### C.3 Non-productive consumption of the protic additive and the amination agent

The consumption of amine electrophile and/or alcohol substrates in non-productive side steps with the {P^P}Cu^I^ hydride describe obstacles for the formal HA catalysis that needs to be addressed in the interest of maintaining uncompromised catalyst performance. Not only would the substrate concentration be lowered to non-appreciable amounts, the entire avenue for direct and/or reductive HA could become shut down completely, if these steps would be massively privileged kinetically. With this in mind, the protonolysis of ethanol R and also the reduction of the hydroxylamine ester A have been subjected to computational analysis.

**Fig. 13:**
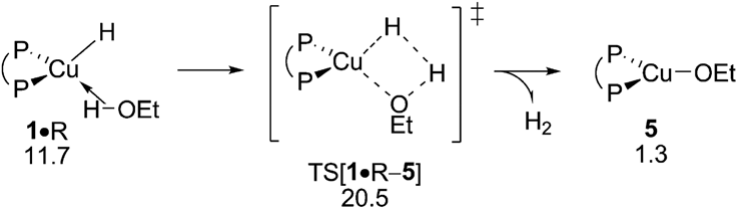
Protonolysis of ethanol by the {P^P}Cu^I^ hydride.^19,20^ Free energies are given in kcal mol^–1^ relative to {½**1dim** + reactants}.

The first step along the protonolysis pathway is ethanol addition to **1** to give weakly bound adduct **1·**R and to evolve thereafter through metathesis-type TS[**1·**R–**5**] describing H_2_ formation taking place in close proximity to the copper centre and has the H_2_ already preformed (see Fig. S32†). The further progression of the process sees the facile liberation of H_2_ (1 equiv.) to deliver {P^P}Cu^I^ alkoxide **5**. σ-Bond metathesis with hydrosilane would then convert **5** back into **1** (see Section B.2). The assessed barrier (Δ*G* ^‡^ = 20.5 kcal mol^–1^ relative to {½**1dim** + R}, Fig. 13) indicates that ethanol R can compete with alkyne S for the {P^P}Cu^I^ hydride with alcohol protonolysis perhaps somewhat more accessible than alkyne hydrocupration (Δ*G* ^‡^ = 21.0 kcal mol^–1^ relative to {½**1dim** + S}, Fig. 2). Hence, the successful generation of alkylamines *via* the route for reductive HA demands a rather highly rapid alkyne hydrometalation with less reactive alkyne substrate/catalyst combinations have no prospect of achieving this goal.

**Fig. 14:**
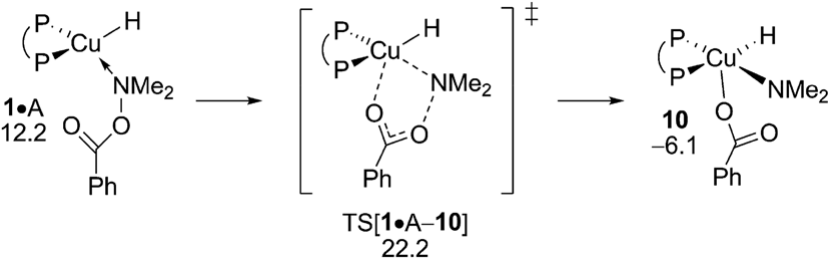
S_N_2-like displacement of the benzoate leaving group at amine adduct **1·**A of the {P^P}Cu^I^ hydride through a multicentre TS structure.^19,20^ Free energies are given in kcal mol^–1^ relative to {½**1dim** + reactants}.

The inspection of the {P^P}CuH-triggered reduction of the hydroxylamine ester is guided by the insight gained above from analysing electrophilic amination at **2** and **6**, respectively. Accordingly, the S_N_2-type benzoate group displacement at amine adduct **1·**A *via* a multicentre TS has been solely examined (see Fig. S33†). As it was noted in our previous computational study on formal HA of styrene by a similar {P^P}Cu based catalyst,^17^ this TS structure is the point of highest energy traversed and hence determines the overall process barrier with ensuing reductive amine elimination was found likely occurring instantaneously in a nearly barrierless fashion. The well-reported aptitude of A to be reduced by the copper hydride is pleasantly mirrored by the derived affordable barrier (Δ*G* ^‡^ = 22.2 kcal mol^–1^ relative to {½**1dim** + A}, Fig. 14), although implying that alcohol protonolysis nonetheless is somewhat more accessible. On the other hand, the predicted narrow gap with regard to the turnover-limiting **4** + H → **1** + (OMe)_2_MeSiOBz transmetalation (ΔΔ*G* ^‡^ = 0.3 kcal mol^–1^ in favour of the latter) is indicative of the close energy rivalry between productive and non-productive branches of the HA catalysis. All in all, the derived rather close range of barriers predicted for crucial steps involved, in productive and non-productive branches, reflects the subtle balance that needs to be struck among rival pathways together with a careful selection of the catalyst/substrate combination and appropriate conditions to successfully achieve the CuH-mediated formal HA of alkynes.

### D Proposed catalytic cycle

**Scheme 4:**
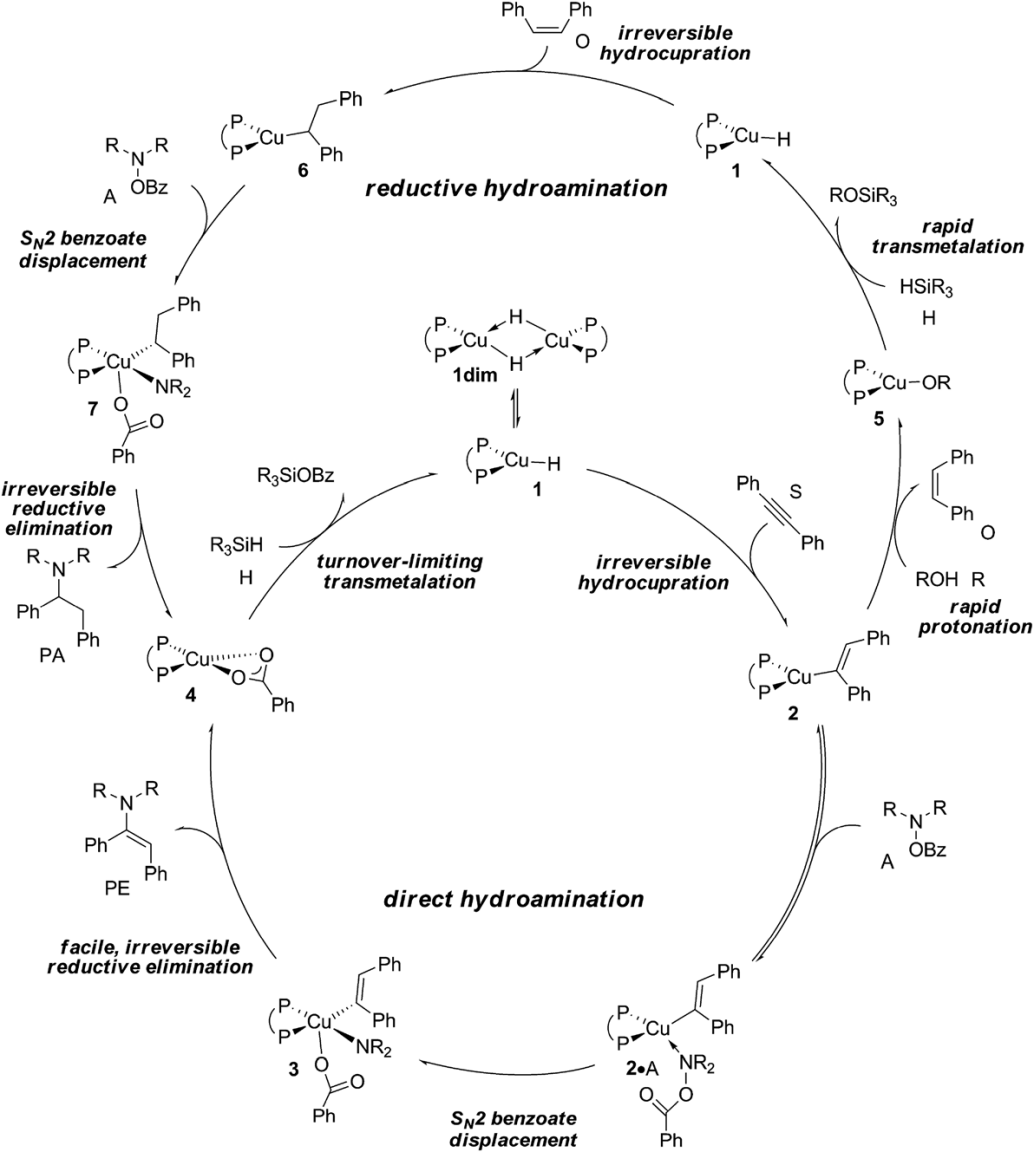
Proposed catalytic cycles for CuH-catalysed direct and reductive hydroamination of internal arylalkynes with hydroxylamine esters.

The elucidation of plausible mechanistic routes presented thus far lead us to the following conclusions (Scheme 4). The {P^P}Cu^I^ hydride **1** favours its dimer **1dim**, but the monomer is the direct precursor for hydrometalation and also non-productive consumption of substrates A and R, hence representing the catalytically competent species. Hydrocupration of alkyne S has an affordable barrier (Δ*G* ^‡^ = 21.0 kcal mol^–1^ relative to {½**1dim** + S}) to yield vinylcopper **2** in a strongly downhill, irreversible transformation. Considering the avenue for direct HA first (see Fig. S34† for its reaction profile) the hydroxylamine electrophile approaches the vinylcopper nucleophile **2** to afford (*E*)-enamine PE and {P^P}Cu^I^ benzoate **4**. Among the several probed mechanistic pathways, N–O bond cleavage involving a multicentre S_N_2-like TS structure (Δ*G* ^‡^ = 16.6 kcal mol^–1^ relative to {**2** + A}) that is linked to an almost instantaneously happening, low-barrier, irreversible reductive elimination of PE from the thus formed short-lived {P^P}Cu^III^ intermediate **3** prevails. Owing to the substantial thermodynamic force associated with electrophilic amination, **4**, which is shared among the rival avenues (Scheme 3), becomes the most stable intermediate traversed along the avenues for direct HA and reductive HA and therefore likely represents the catalyst resting state. The catalytic cycle is completed by transmetalation of **4** with hydrosilane evolving through a six-centre TS structure. This TS defines the point of highest energy to be crossed along the route for direct HA and reductive HA catalysis. Hence, **4** + H → **1** + (OMe)_2_MeSiOBz transmetalation featuring a barrier of 21.9 kcal mol^–1^ (Δ*G* ^‡^ relative to {**4** + H}) is likely turnover limiting for both avenues (see Fig S34†).^24^ We note that recent complementary experimental^21^ and computational^17^ studies revealed that relative rates for crucial hydrocupration, amination and transmetalation steps follow a similar order in the formal HA of styrene by related {P^P}Cu^I^ catalyst systems.

As far as the chemodivergent avenue for reductive HA is concerned, vinylcopper nucleophile **2** is found capable of engaging at affordable energy costs not only with amine electrophile A (see above) but also with ethanol additive R. Protonation at **2** (Δ*G* ^‡^ = 15.5 kcal mol^–1^ relative to {**2** + R}) releases *cis*-stilbene Q and forms {P^P}Cu^I^ ethoxide **5**, which is converted back into **1** thereafter through facile σ-bond metathesis with hydrosilane H; an overall transformation that is strongly downhill (Δ*G* = –26.6 kcal mol^–1^ relative to {**2** + A + H}). The *cis*-alkene Q can compete with alkyne S for migratory insertion into the {P^P}Cu–H linkage, although found somewhat slower (Δ*G* ^‡^ = 21.5 kcal mol^–1^ relative to {**1** + Q}) and less favourable thermodynamically than S. Among rival pathways for the thus formed alkylcopper **6** to be approached by either amine electrophile or alcohol the former prevails energetically. Similar to the findings for vinylcopper **2** electrophilic amination of **6** preferably evolves through a multicentre TS describing S_N_2-type benzoate group displacement (Δ*G* ^‡^ = 13.2 kcal mol^–1^ relative to {**6** + A}) to be followed by kinetically easy, irreversible reductive elimination of alkylamine PA commencing at transient {P^P}Cu^III^ intermediate **7**. Finally, turnover-limiting transmetalation with hydrosilane H regenerates {P^P}Cu^I^ hydride **1** from **4** for another catalyst turnover (see Fig. S35†).^24,25^

**Fig. 15:**
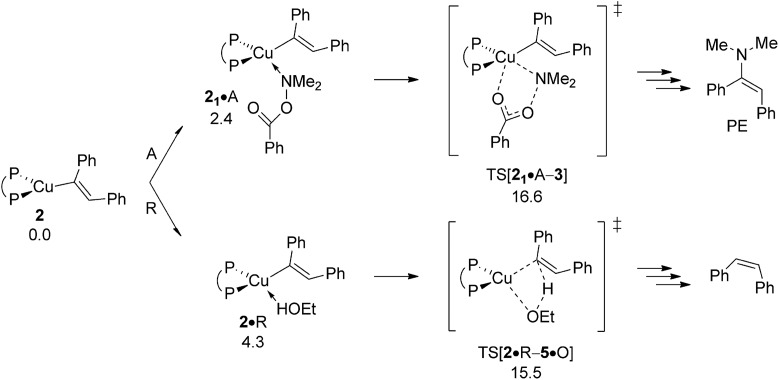
Propensity of {P^P}Cu^I^ vinyl **2** to engage with amine electrophile or alcohol.^19^ Free energies are given in kcal mol^–1^ relative to {**2** + reactants}.

**Fig. 16:**
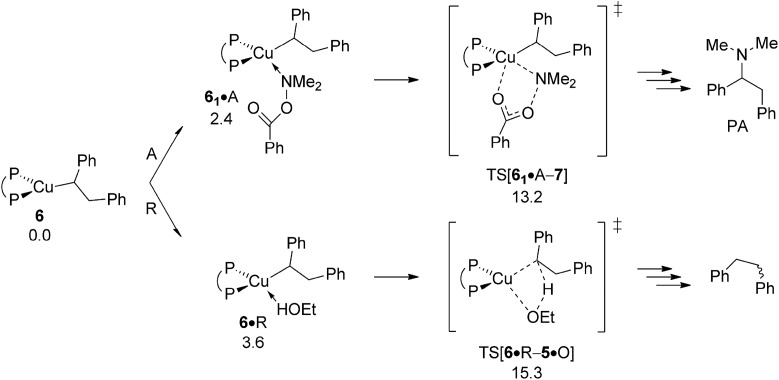
Propensity of {P^P}Cu^I^ alkyl **6** to engage with amine electrophile or alcohol.^19^ Free energies are given in kcal mol^–1^ relative to {**6** + reactants}.

As already outlined above, the successful generation of alkylamine PA would require for **2** and **6** displaying chemodivergence in their reactivity toward amine electrophile and alcohol. Fig. 15 reveals that {P^P}Cu^I^ vinyl **2** favours R somewhat over A, thereby rendering the avenue for reductive HA accessible and prevalent in the presence of both amine electrophile and alcohol.^26^ In contrast, the higher kinetic demands for protonation rather than electrophilic amination predicted for {P^P}Cu^I^ alkyl **6** (Fig. 16) prevents the avenue for reductive HA to become non-productive.

### E Effect of the amine electrophile upon the chemoselectivity

**Fig. 17:**
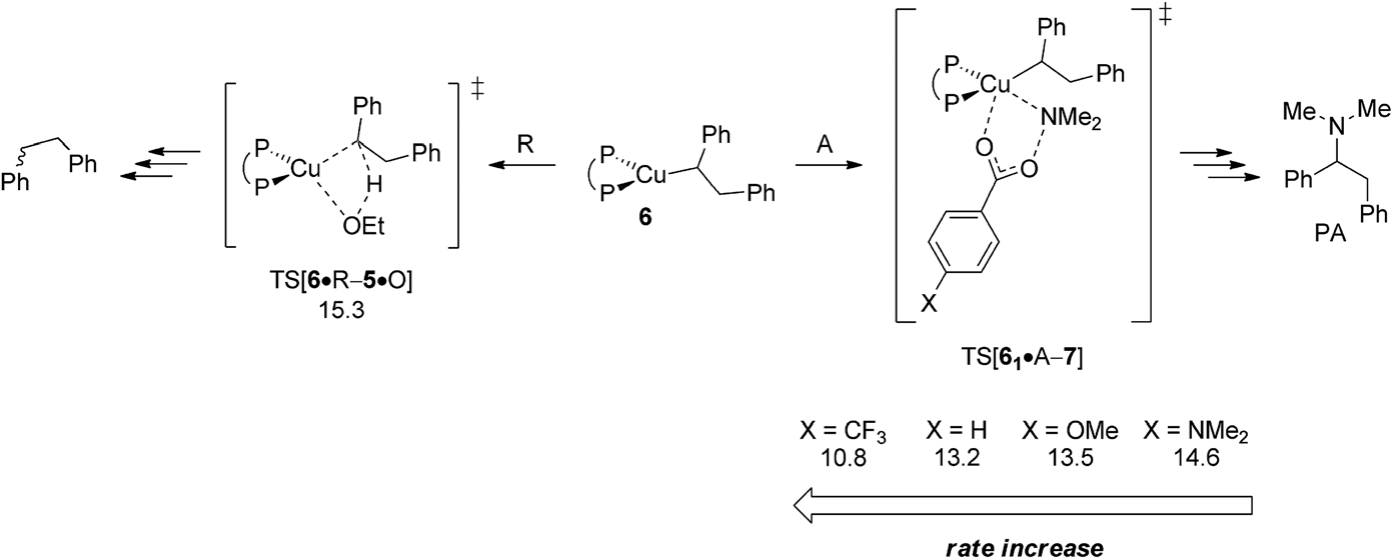
Kinetic demands of rival pathways commencing at {P^P}Cu^I^ vinyl **2** for several electronically modified hydroxylamine esters.^19^ Free energies are given in kcal mol^–1^ relative to {**2** + reactants}.

**Fig. 18:**
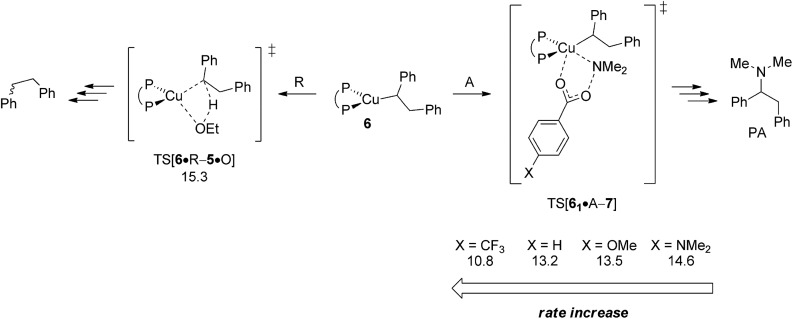
Kinetic demands of rival pathways commencing at {P^P}Cu^I^ alkyl **6** for several electronically modified hydroxylamine esters.^19^ Free energies are given in kcal mol^–1^ relative to {**6** + reactants}.

In an attempt of enhancing our understanding of catalyst structure–performance relationships further this final part explores to what extent the chemoselectivity of the process can be influenced by suitably selected reagents. A possible shift in the relative propensity of vinylcopper **2** and alkylcopper **6** to be approached by either amine electrophile or alcohol additive is centre to our interest. Ethanol has already been demonstrated as the preferable alcohol additive,^13*b*^ so that rather little potential for improvement can be anticipated there. Accordingly, the barrier for S_N_2-type electrophilic amination has been evaluated for different electronically modified amine-*O*-benzoates featuring archetype electron-releasing (X = OMe, NMe_2_) or electron-withdrawing (X = CF_3_) *para*-phenyl substituents. The overall order of assessed reactivity for vinylcopper **2** (Fig. 17) is for amination to become accelerated by an electron-withdrawing substituent, which can be rationalised by a stabilisation of the multicentre TS structure by a more electrophilic N_amine_, with a more electron-rich amination reagent slowing the process down. All in all, electrophilic amination becomes the more retarded the more effective the donor substituent is. As it could reasonably be anticipated, alkylcopper **6** displays a qualitatively similar behaviour (Fig. 18).

A closer look at the trends in reactivity predicted for **2** and **6** provides some valuable insights. Protonation of **2** and **6** has an almost identical barrier to traverse. In contrast, electrophilic amination of **2**, when compared to **6**, not only has a greater kinetic demand, but also shows a larger variance when electronically modified hydroxylamine esters are involved. Electron-poor amination agents do not appear beneficiary for the generation of alkylamines, because the reductive HA course is likely less accessible in such cases (Fig. 17). On the other hand, moderately electron-rich hydroxylamine esters should be suitable, not only for maintaining the fine balance of chemodivergent reactivity required for the successful formation of (*E*)-enamines and alkylamines, but also to accelerate the turnover-limiting conversion of **4** back into **1**, as revealed by a recent computational study on a similar {P^P}Cu^I^ catalyst system.^17^

## Conclusions

The mechanism operative for the conceptually novel strategy for CuH-mediated formal hydroamination of 1,2-diphenylacetylene with prototype hydroxylamine ester and hydrosilane has been probed computationally. A reliable, well benchmarked DFT technique has been employed to identify intimate details of all key steps for the direct HA course as well as for the rival avenue of reductive HA, which becomes available if the protic additive is present. The mechanistic scenario (Scheme 4) proposed herein, which is manifest in smooth energy profiles, conforms to all available experimental data. Alkyne hydrocupration to afford vinylcopper **2** is kinetically affordable and irreversible. In the absence of a protic alcohol additive, nucleophile **2** approaches the hydroxylamine electrophile to yield the (*E*)-enamine and {P^P}Cu^I^ benzoate **4** by S_N_2-type displacement of the benzoate leaving group through a multicentre TS structure and strongly downhill, fast reductive elimination thereafter. Transmetalation of **4**, which likely corresponds to the catalyst resting state, with hydrosilane regenerates the catalytically competent {P^P}Cu^I^ hydride. If alcohol is present, then the avenue for reductive HA becomes available through the initial facile protonation of **2** to deliver the {P^P}Cu^I^ alkoxide with the release of *cis*-alkene. Regeneration of the {P^P}Cu^I^ hydride through facile σ-bond metathesis with hydrosilane and *cis*-alkene insertion into the Cu–H linkage thereafter generates alkylcopper **6**. Coupling with the amine electrophile, following a pathway similar to the one unveiled for electrophilic amination of **2**, delivers the alkylamine product together with {P^P}Cu^I^ benzoate **4**, which is shared among the rival avenues. Reduction of the (*E*)-enamine could also lead to the alkylamine, but found kinetically non-competitive. Furthermore, catalyst performance is unlikely compromised by the generation of diamination side products due to an associated demanding kinetic profile.

The crucial vinylcopper **2** and alkylcopper **6** intermediates are found to display a distinct chemodivergence in reactivity towards amine electrophile and alcohol, thereby making the generation of alkylamine PA together with (*E*)-enamine PE achievable. On the one hand, vinylcopper **2** is found somewhat more favourably approached by alcohol than by hydroxylamine ester, thus rendering the avenue for reductive HA accessible and prevalent in the presence of both amine electrophile and alcohol. On the contrary, the higher kinetic demands for protonation rather than electrophilic amination predicted for alkylcopper **6** prevents the avenue for reductive HA to become non-productive. Electronically modified hydroxylamine esters effect the rate for electrophilic amination of vinylcopper **2** and alkylcopper **6** in a similar fashion, with more electron-rich agents decelerate the process. However, among the two crucial intermediates, **2** not only inflicts the greater kinetic demands but also shows a larger variation upon electronic variation. Moderately electron-rich amine-*O*-benzoates are expected to be most suitable for the generation of alkylamine and enamine products, whilst electron-poor agents do not appear beneficiary for the generation of alkylamines.

The detailed delineation of mechanistic intricacies and a more fundamental insight into catalytic structure–performance relationships together with guidelines for rational process improvement provided herein will likely pave the way for the rational design of copper-based systems engaging in formal HA catalysis.
