# Supplementary material for: CuH-catalysed hydroamination of arylalkynes with hydroxylamine esters – a computational scrutiny of rival mechanistic pathways
Source: Chem Sci. 2017 Apr 28;8(6):4410–23. doi: 10.1039/c7sc01107e (PMC5472847; doi:10.1039/c7sc01107e)
Supplement: Supplementary file 1 [file SC-008-C7SC01107E-s001.pdf]

Electronic Supplementary Material (ESI) for Chemical Science
This journal is © The Royal Society of Chemistry 2017

SUPPLEMENTARY INFORMATION

CuH-Catalysed Hydroamination of Arylalkynes with Hydroxylamines – A Computational Scrutiny of Rival Mechanistic Pathways

Sven Tobisch[a]

[a] *School of Chemistry, University of St Andrews, Purdie Building, North Haugh, St Andrews, KY16 9ST, United Kingdom, E-mail: st40@st-andrews.ac.uk*

Computational Details

All calculations based on Kohn-Sham density functional theory (DFT)[1] were performed by means of the program package TURBOMOLE[2] employing flexible basis sets of triple-ζ quality. The Becke-Perdew (BP86)[3] generalised gradient approximation (GGA) functional within the RI-*J* integral approximation[4] in conjunction with appropriate auxiliary basis sets was used for structure optimisation. Empirical atom-pairwise dispersion corrections by Grimme (D3 with Becke-Johnson damping)[5] were used to account for critical non-covalent interactions. Copper was treated by the (17s12p7d2f)/[6s5p2d1f] (def2-TZVPP, excluding the g polarisation function) all-electron basis set,[6a,b] whilst all remaining elements were represented by Ahlrich's valence triple-ζ def2-TZVP basis set[6a,b] with polarisation functions on all atoms. Final potential energies were obtained by single point calculations at BP86 optimised structures using the hybrid *meta*-GGA PW6B95[7] functional (together with D3(BJ) empirical dispersion correction)[5] in conjunction with the aforementioned basis sets (PW6B95-D3/(def2-TZVPP + def2-TZVP)//BP86-D3/(def2-TZVPP + def2-TZVP)). A large integration grid (*m4* in TURBOMOLE notation) and tight SCF convergence criteria have been used.

The reaction pathways were explored by a chain-of-states method,[8] as implemented in the module *woelfling* in the TURBOMOLE suite of programs, which makes use of reasonably chosen reactant and product structures to deliver an approximate to the minimum-energy path (MEP). It identified the reactant and product states to be linked to the associated transition state. The approximate saddle points connected with the MEP were subjected to an exact localisation of the TS structures. The geometry optimisation and the saddle-point search were carried out by utilising analytical gradients/Hessians according to standard algorithms. No symmetry constraints were imposed in any case. The stationary points were identified exactly by the curvature of the potential energy surface at these points corresponding to the eigenvalues of the Hessian. All reported TS structures possess exactly one negative Hessian eigenvalue, while all other stationary points exhibit exclusively positive eigenvalues.

Aimed at providing substantive support for one of the several rival mechanistic pathways, the present study explored comprehensively alternative mechanistic avenues for direct and reductive hydroamination of 1,2-diphenylacethylene (S) with *O*-benzoyl-*N*,*N*-dimethyl-hydroxylamine (A) and prototype dimethoxy-methylsilane (H) by a catalytically competent Xantphos-ligated Cu^{I} hydride compound, with or without ethanol (R) as protic alcohol additive present, which was recently reported by the group of Buchwald.[9] For the sole purpose of computational efficiency, *O*-benzoyl,*N*,*N*-dibenzyl-hydroxylamine and diethoxy-methylsilane used in experiment were replaced by A and H, respectively. No further simplifications of any kind have been imposed for any of the key species involved. The DFT calculations have simulated the authentic reaction conditions by treating the bulk effects of the THF solvent by a consistent continuum model in form of the conductor-like screening model for realistic solvents (COSMO-RS).[10] This solvation model includes continuum electrostatic and also solvent-cavitation and solute-solvent dispersion effects through surface-proportional terms and also refers properly to a 1 M standard state. The free solvation enthalpy has been assessed with the aid of COSMO-RS[10] as implemented in

COSMO*therm*[11] at the BP86/(def2-TZVPD)//BP86-D3/(def2-TZVPP + def2-TZVP)[6a,b] level of approximation. Frequency calculations were performed for stationary points that were located at the BP86-D3/(def2-TZVPP + SV(P))[6c] level to confirm the nature of all optimised key structures and to determine thermodynamic parameters (318 K, 1 atm) under the conventional ideal-gas approximation. This level of basis set quality is known to be reliable for the assessment of structural parameter and vibrational frequencies,[12] thus it allows an affordable and accurate determination of thermodynamic state functions. As far as the vibrational partition function is concerned, a modified rigid-rotor-harmonic oscillator scheme was used.[13] In this approach, vibrational modes below 60 cm^{-1} were treated within a rigid-rotor model with smooth interpolation to the conventional harmonic oscillator regime. The final Gibbs free energies (ΔG) were determined from gas-phase single point PW6B95 electronic energies, plus BP86-derived thermochemical contributions to enthalpy and entropy ΔG_{mRRHO} and COSMO-RS solvation free enthalpies $\Delta\delta G_{solv}$: $\Delta G = \Delta E + \Delta G_{mRRHO} + \Delta\delta G_{solv}$. Calculated structures were visualised by employing the StrukEd program,[14] which was also used for the preparation of 3D molecule drawings.

The computational methodology employed (reliable hybrid *meta*-GGA PW6B95 functional in conjunction with flexible basis sets of def2-TZVP quality and a sound treatment of bulk solvent effects) simulated authentic reaction conditions adequately and the mechanistic analysis was based on Gibbs free-energy profiles assessed at the PW6B95-D3(COSMO-RS)/(def2-TZVPP + def2-TZVP)//BP86-D3/(def2-TZVPP + def2-TZVP)) level of approximation for experimental condensed-phase conditions. The validity of the computational protocol employed for reliably mapping the energy landscape of CuH-mediated hydroamination has been substantiated before,[15] and this allowed mechanistic conclusions with substantial predictive value to be drawn.

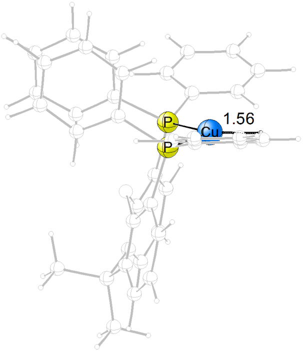

1

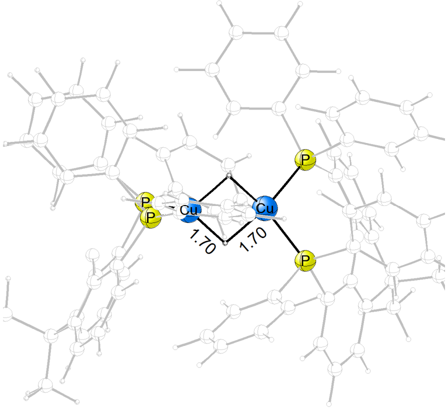

1dim

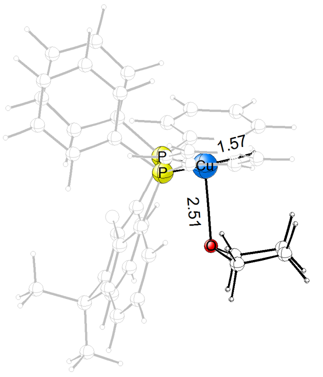

1•T

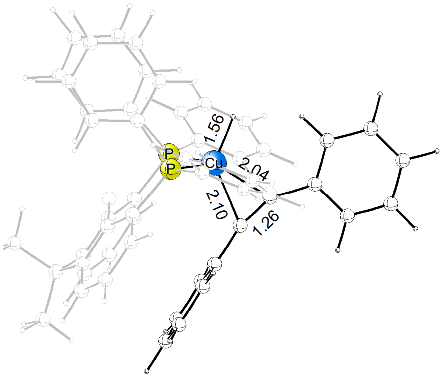

1•S

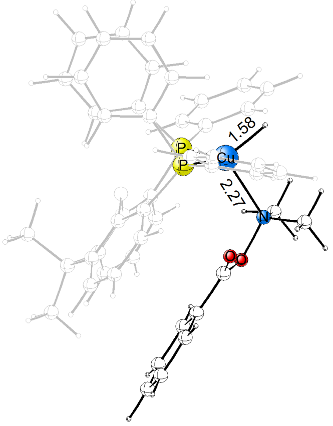

1•A

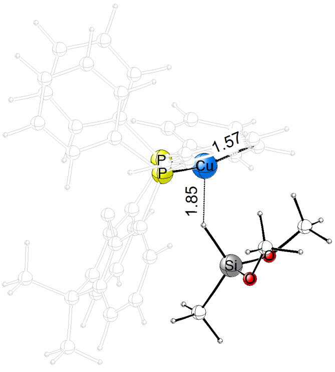

1•H

Figure S1. Selected structural parameter (angstrom) of the optimised structures of various forms of the catalytically competent {P^P}Cu^{I} hydride **1**.

The Xantphos ligand is greyed out to enhance the visualisation of crucial structural aspects**.**

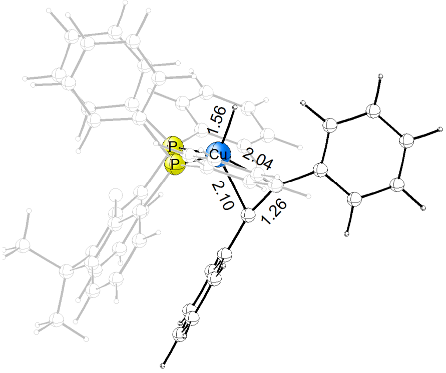

1•S

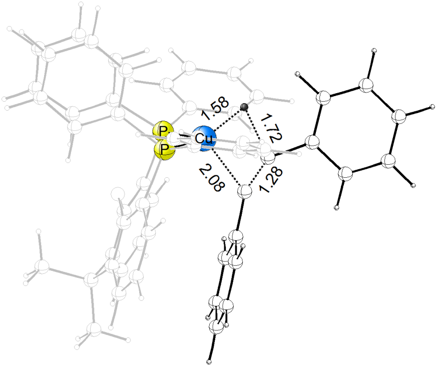

TS[**1**•S–**2**]

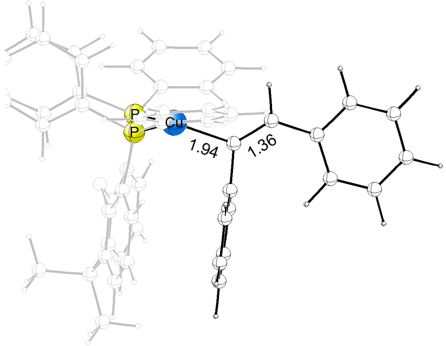

2

Figure S2. Selected structural parameter (angstrom) of the optimised structures of key stationary points for alkyne C≡C bond insertion into the Cu–H linkage at alkyne adduct **1**•S of the {P^P}Cu^{I} hydride.

The Xantphos ligand is greyed out to enhance the visualisation of crucial structural aspects**.**

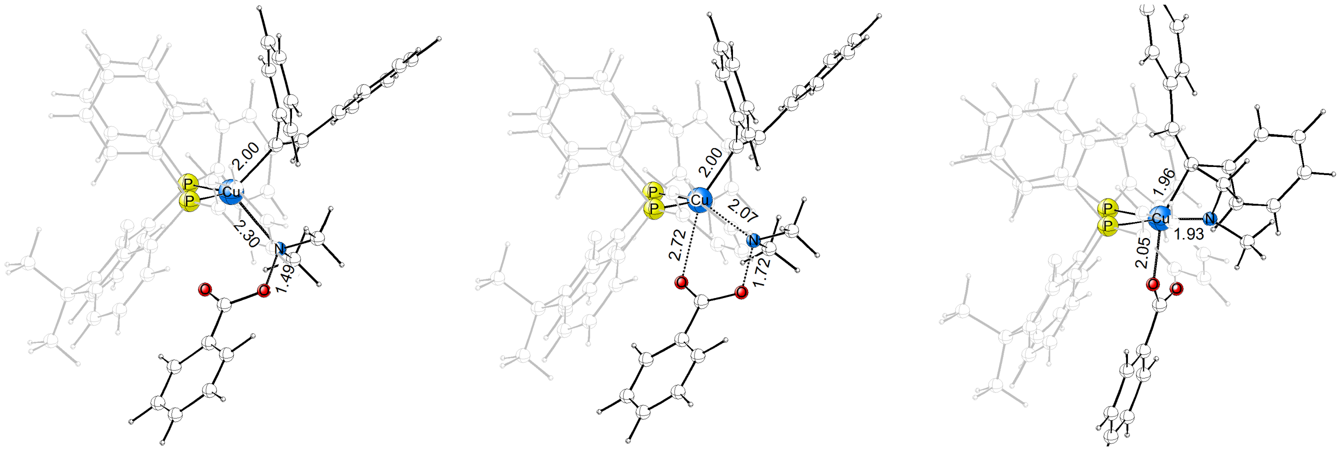

2_1•A TS[**2_1**•A–**3**] **3**

Figure S3. Selected structural parameter (angstrom) of the optimised structures of key stationary points for S_N2 displacement of the benzoate leaving group via a multicentre TS structure at amine adduct **2**•A of the (P^P)Cu^I vinyl intermediate.

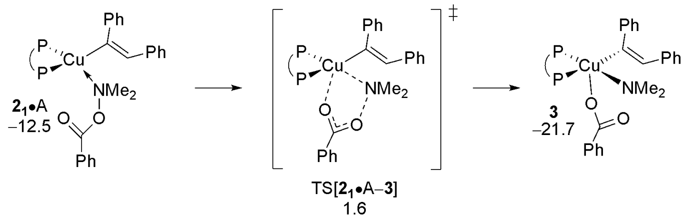

Figure S4. Cleavage of the hydroxylamine ester N–O linkage through S_N2 displacement of the benzoate leaving group involving a multicentre TS structure at amine adduct **2**•A of the (P^P)Cu^I vinyl intermediate.[16] Free energies are given in kcal mol^{-1} relative to {½**1dim** + reactants}.

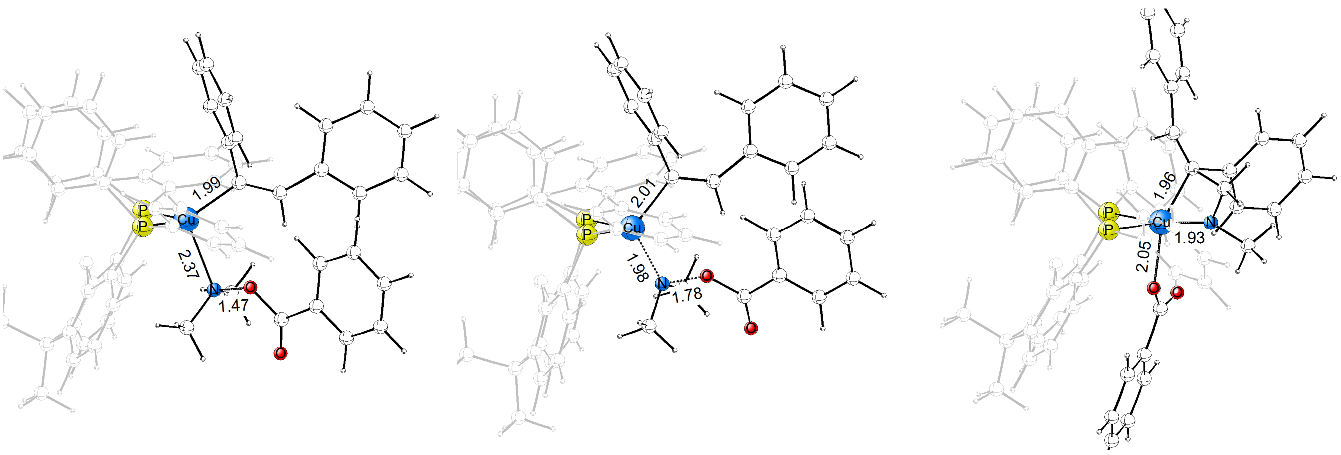

2_2•A TS[**2_2**•A–**3**] **3**

Figure S5. Selected structural parameter (angstrom) of the optimised structures of key stationary points for S_N2 displacement of the benzoate leaving group at amine adduct **2**•A of the (P^P)Cu^I vinyl intermediate.

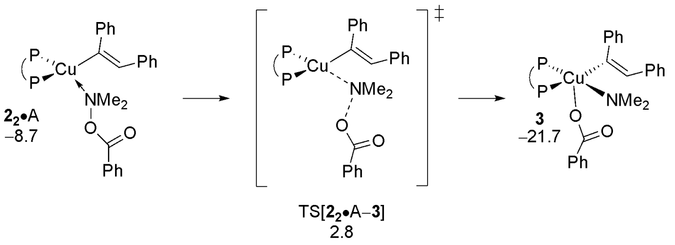

Figure S6. Cleavage of the hydroxylamine ester N–O linkage through S_N2 displacement of the benzoate leaving group at amine adduct **2**•A of the (P^P)Cu^I vinyl intermediate.[16] Free energies are given in kcal mol^{-1} relative to {½**1dim** + reactants}.

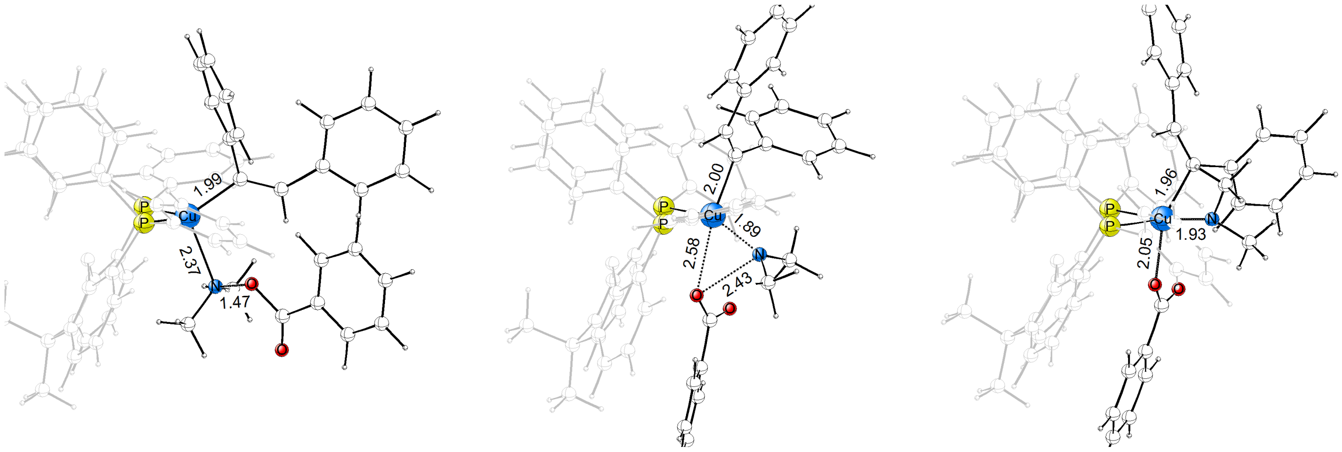

2_2•A TS$_{OA}$[2_2•A–**3**] **3**

Figure S7. Selected structural parameter (angstrom) of the optimised structures of key stationary points for oxidative addition of amine electrophile A across the N–O linkage at amine adduct **2**•A of the (P^P)Cu^I vinyl intermediate.

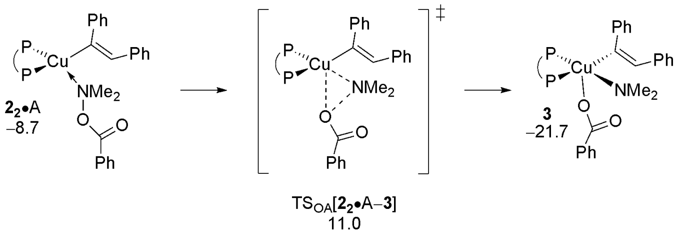

Figure S8. Oxidative addition of hydroxylamine ester A at amine adduct **2**•A of the (P^P)Cu^I vinyl intermediate.[16] Free energies are given in kcal mol^{-1} relative to {½**1dim** + reactants}.

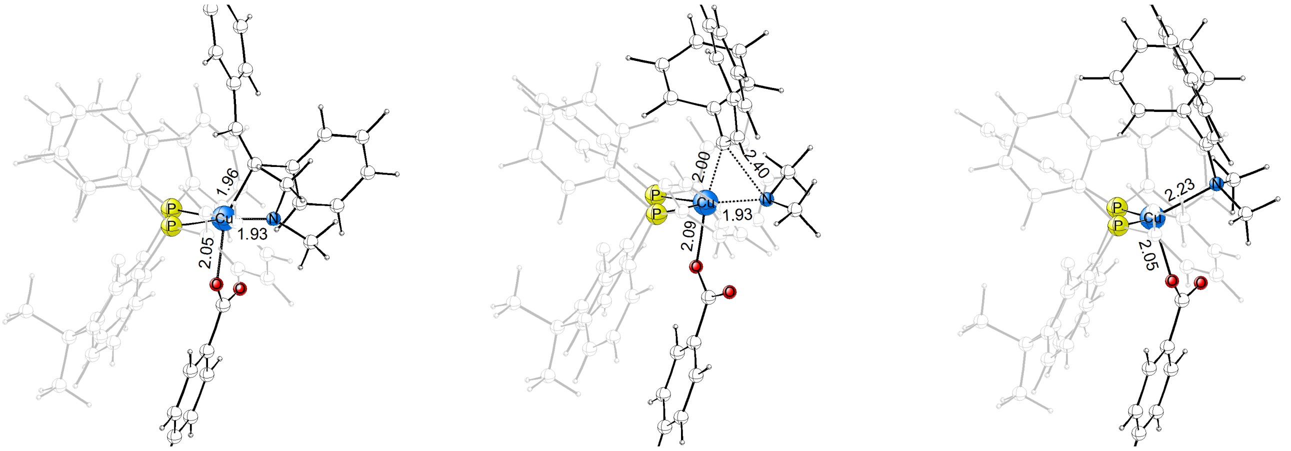

3 TS[**3**–**4**•PE] **4**•PE

Figure S9. Selected structural parameter (angstrom) of the optimised structures of key stationary points for reductive elimination of enamine product PE at {P^P}Cu^{III} vinyl benzoate amido intermediate **3**.

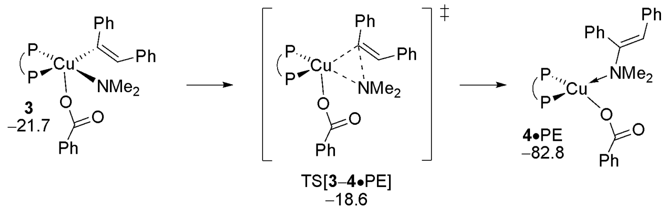

Figure S10. N–C bond-forming reductive elimination at (P^P)Cu^{III} vinyl benzoate amido intermediate **3**.[16] Free energies are given in kcal mol^{-1} relative to {½**1dim** + reactants}.

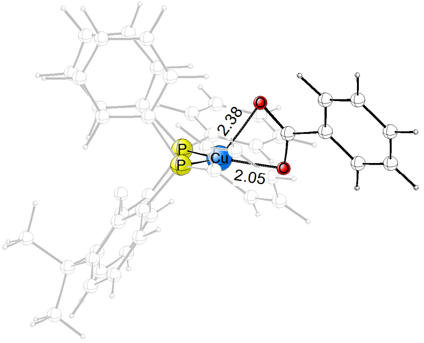

4

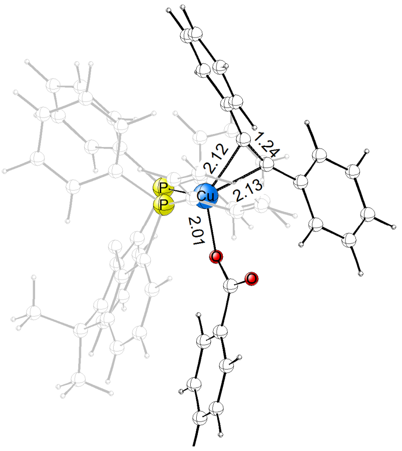

4•S

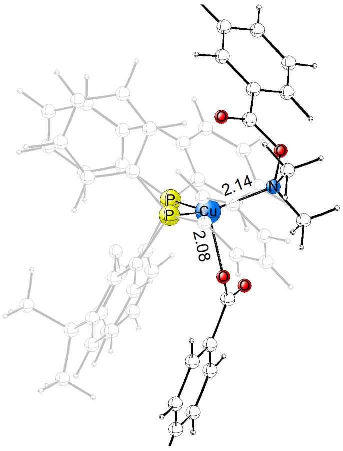

4•A

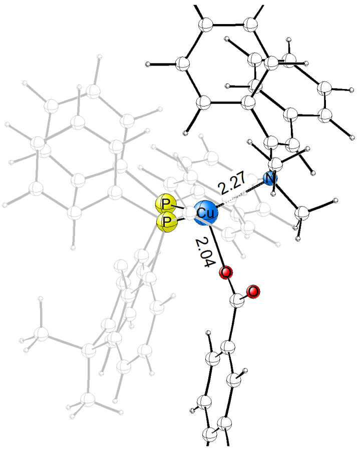

4•PE

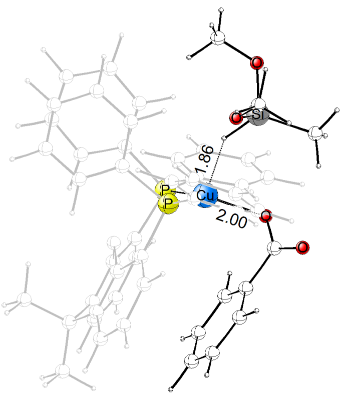

4•H

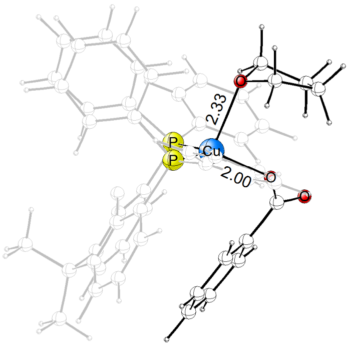

4•T

Figure S11. Selected structural parameter (angstrom) of the optimised structures of various forms of the {P^P}Cu^{I} benzoate **4**.

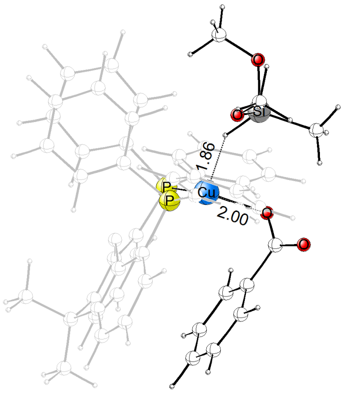

4•H

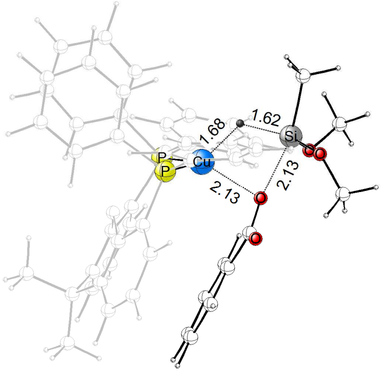

TS[**4**•H–**1**•OS_1]

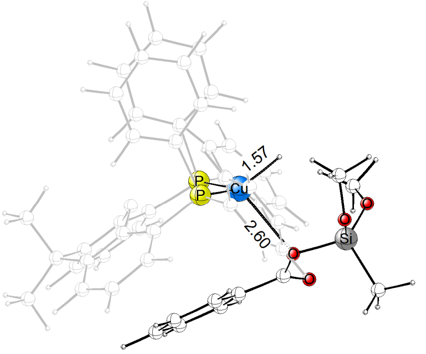

1•OS_1

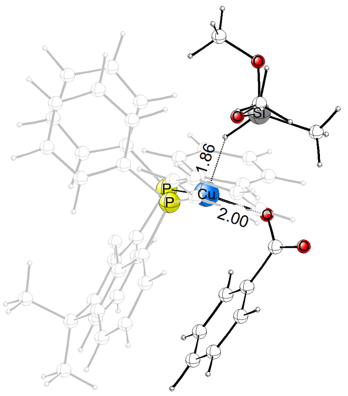

4•H

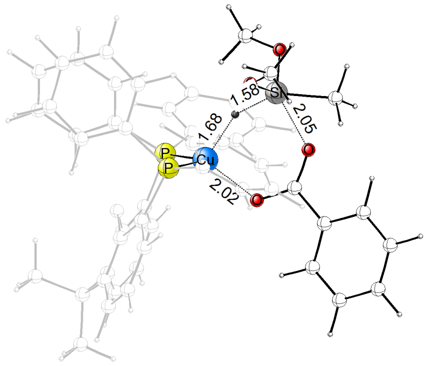

TS[**4**•H–**1**•OS_2]

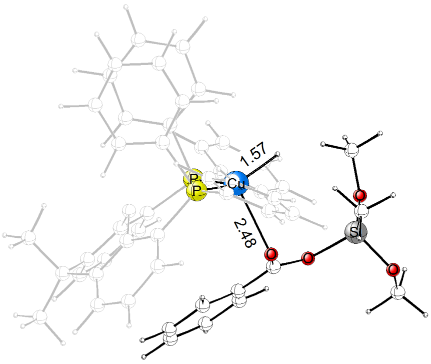

1•OS_2

Figure S12. Selected structural parameter (angstrom) of the optimised structures of key stationary points for transmetalation of {P^P}Cu^I benzoate **4** with dimethoxymethylsilane H.

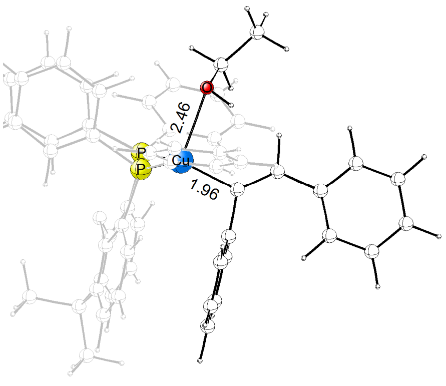

2•R

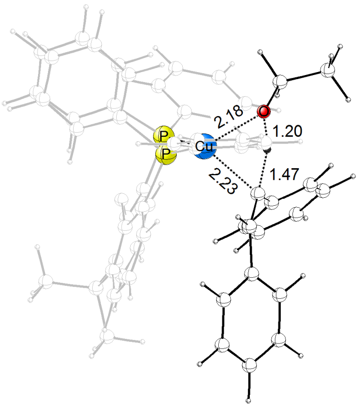

TS[**2**•R–**5**•O]

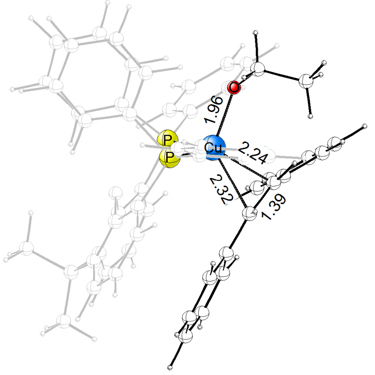

5•O

Figure S13. Selected structural parameter (angstrom) of the optimised structures of key stationary points for protonation of {P^P}Cu^I vinyl **3** by ethanol R.

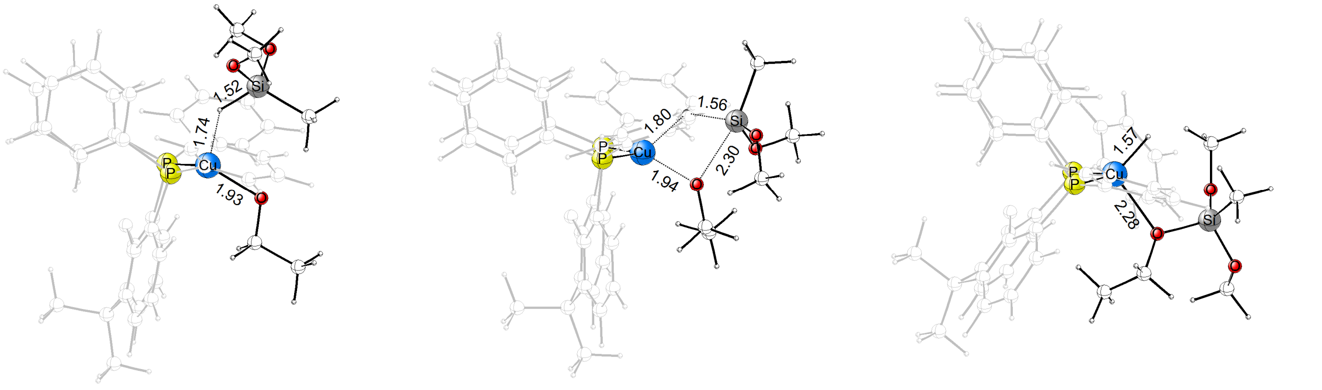

5•H TS[**5**•H–**1**•OS_3] **1**•OS_3

Figure S14. Selected structural parameter (angstrom) of the optimised structures of key stationary points for transmetalation of {P^P}Cu[I] alkoxide **5** with dimethoxymethylsilane H.

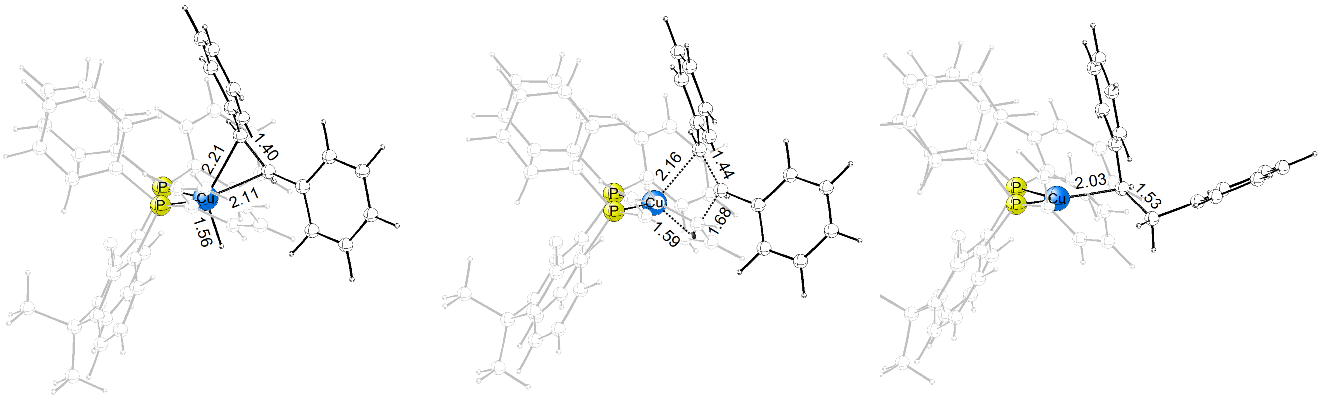

1•O TS[**1**•O–**6**] **6**

Figure S15. Selected structural parameter (angstrom) of the optimised structures of key stationary points for C=C bond insertion into the Cu–H linkage at *cis*-alkene adduct **1**•O of the {P^P}Cu[I] hydride.

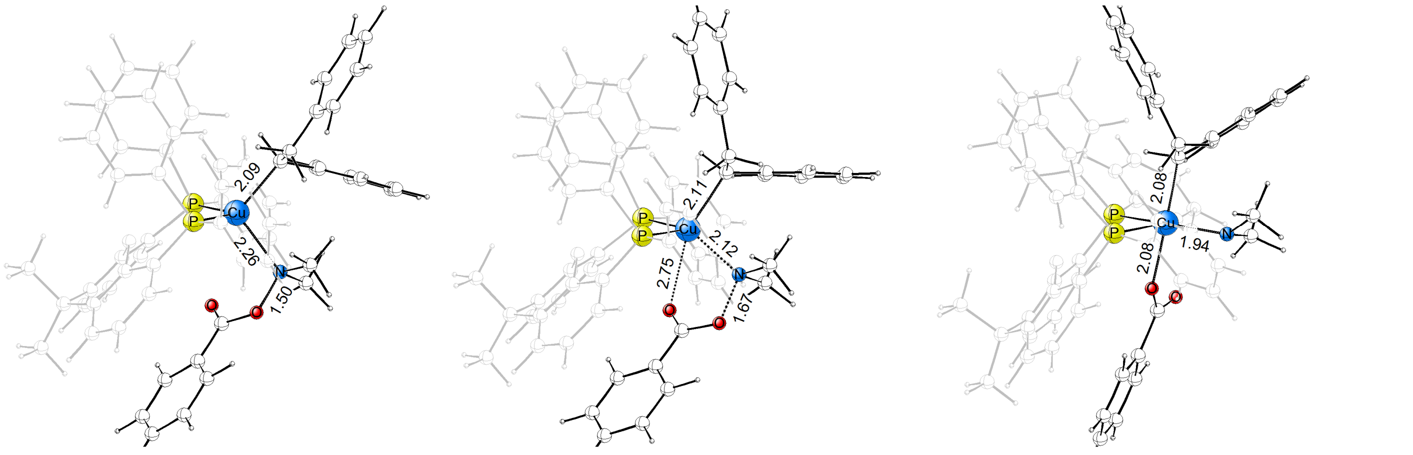

$\mathbf{6_1}$•A TS[$\mathbf{6_1}$•A–**7**] **7**

Figure S16. Selected structural parameter (angstrom) of the optimised structures of key stationary points for S_N2 displacement of the benzoate leaving group via a multicentre TS structure at amine adduct **6**•A of the (P^P)Cu[I] alkyl intermediate.

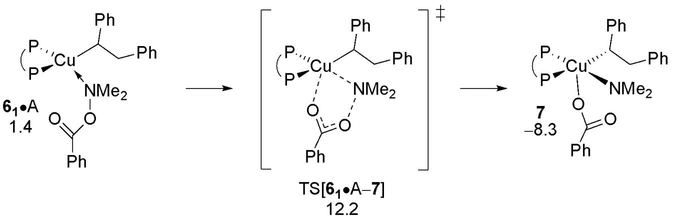

Figure S17. Cleavage of the hydroxylamine ester N–O linkage through S_N2 displacement of the benzoate leaving group at amine adduct **6**•A to involve a multicentre TS structure of the (P^P)Cu^I alkyl intermediate.[16] Free energies are given in kcal mol^{-1} relative to {½**1dim** + reactants}.

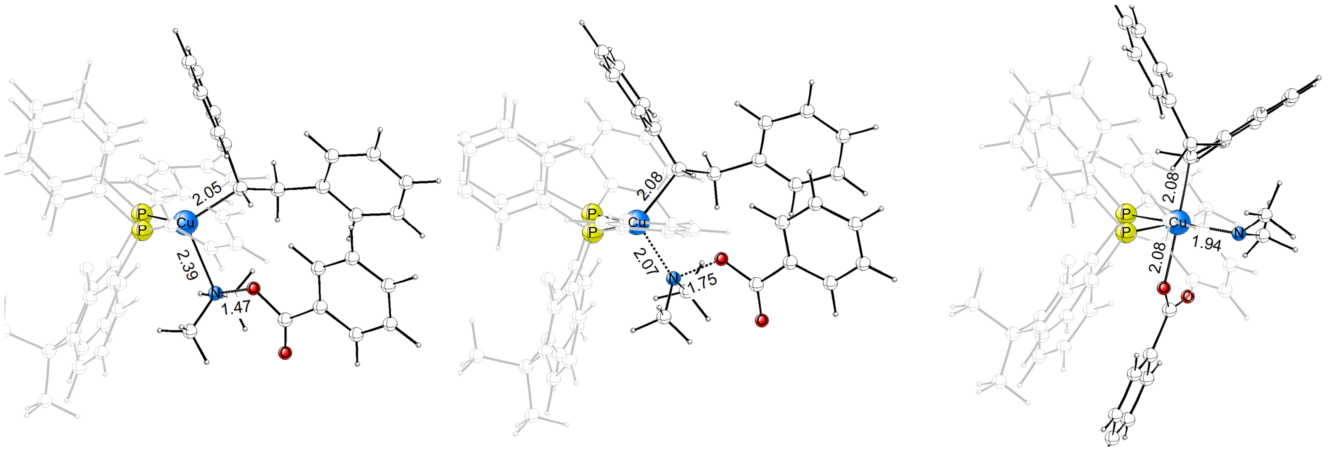

6₂•A TS[**6₂**•A–**7**] **7**

Figure S18. Selected structural parameter (angstrom) of the optimised structures of key stationary points for S_N2 displacement of the benzoate leaving group at amine adduct **6**•A of the (P^P)Cu^I alkyl intermediate.

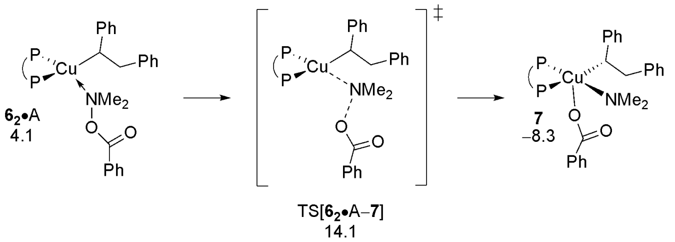

Figure S19. Cleavage of the hydroxylamine ester N–O linkage through S_N2 displacement of the benzoate leaving group at amine adduct **6**•A of the (P^P)Cu^I alkyl intermediate.[16] Free energies are given in kcal mol^{-1} relative to {½**1dim** + reactants}.

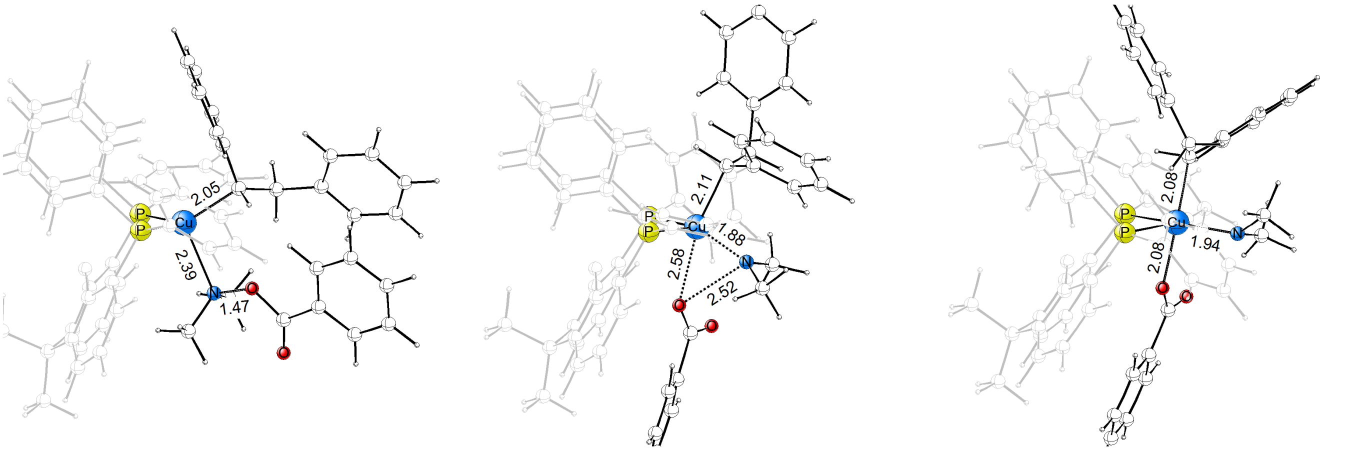

6_2•A TS$_{OA}$[**6_2**•A–**7**] **7**

Figure S20. Selected structural parameter (angstrom) of the optimised structures of key stationary points for oxidative addition of amine electrophile A across the N–O linkage at amine adduct **6**•A of the (P^P)Cu^{I} alkyl intermediate.

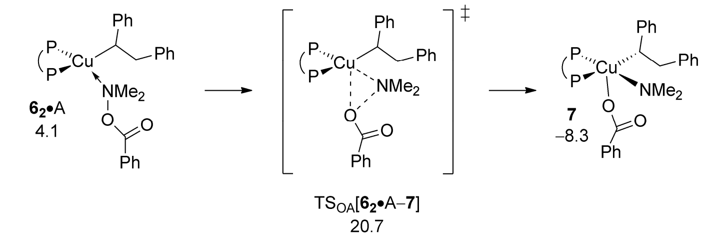

Figure S21. Oxidative addition of hydroxylamine ester A at amine adduct **6**•A of the (P^P)Cu^{I} alkyl intermediate.[16] Free energies are given in kcal mol^{-1} relative to {½**1dim** + reactants}.

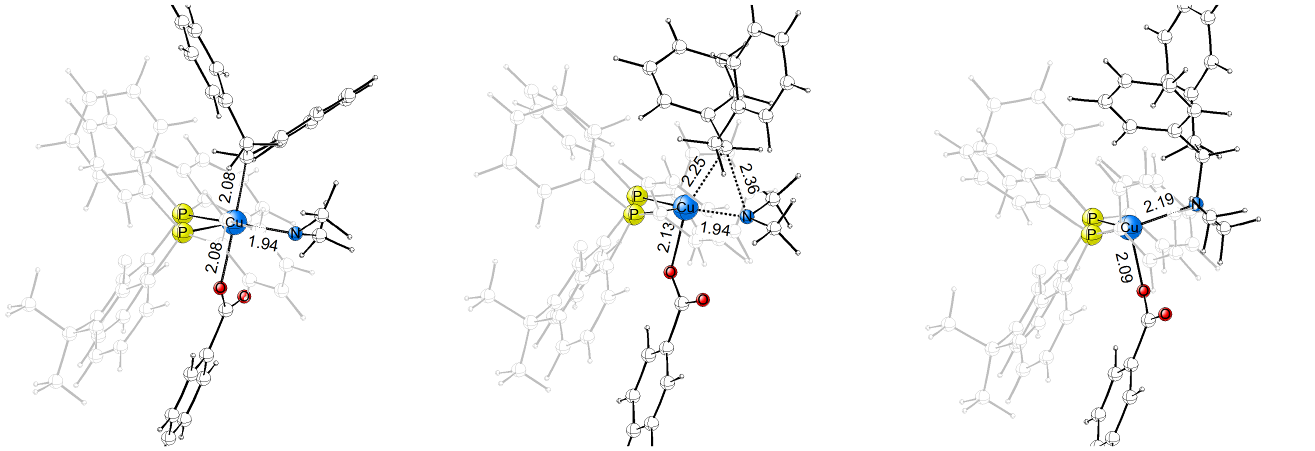

7 TS[**7**–**4**•PA] **4**•PA

Figure S22. Selected structural parameter (angstrom) of the optimised structures of key stationary points for reductive elimination of alkylamine product PA at {P^P}Cu^{III} alkyl benzoate amido intermediate **7**.

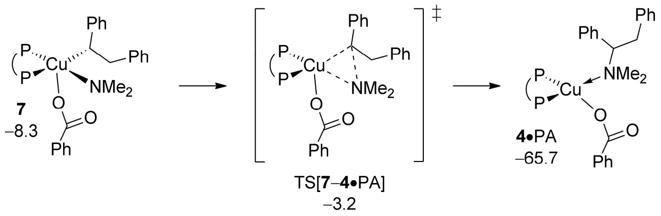

Figure S23. N–C bond-forming reductive elimination at (P^P)Cu^{III} alkyl benzoate amido intermediate **7**.[16] Free energies are given in kcal mol^{-1} relative to {½**1dim** + reactants}.

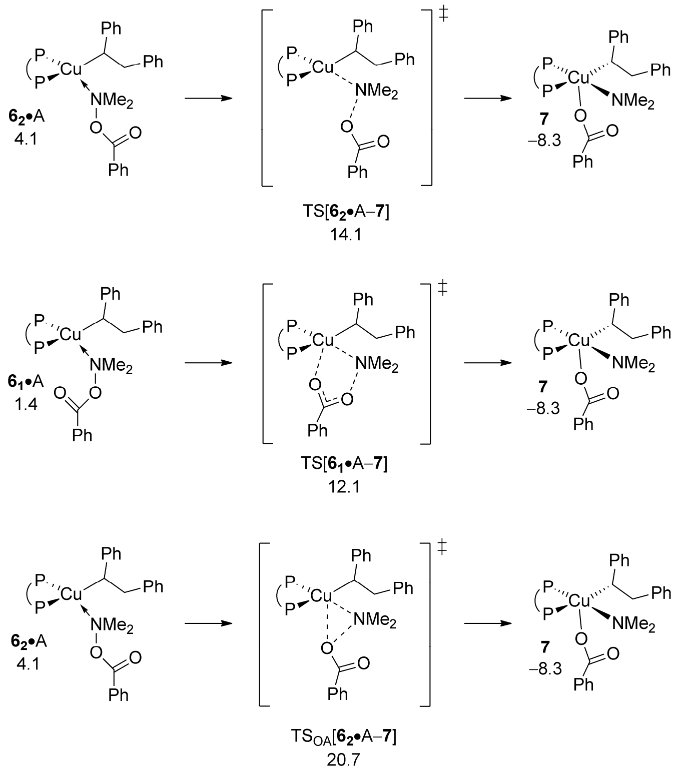

Figure S24. Most accessible pathways for cleavage of the hydroxylamine ester N–O linkage at amine adduct **6**•A of the (P^P)Cu^{I} alkyl intermediate through alternative mechanistic pathways.[16] Free energies are given in kcal mol^{-1} relative to {½**1dim** + reactants}.

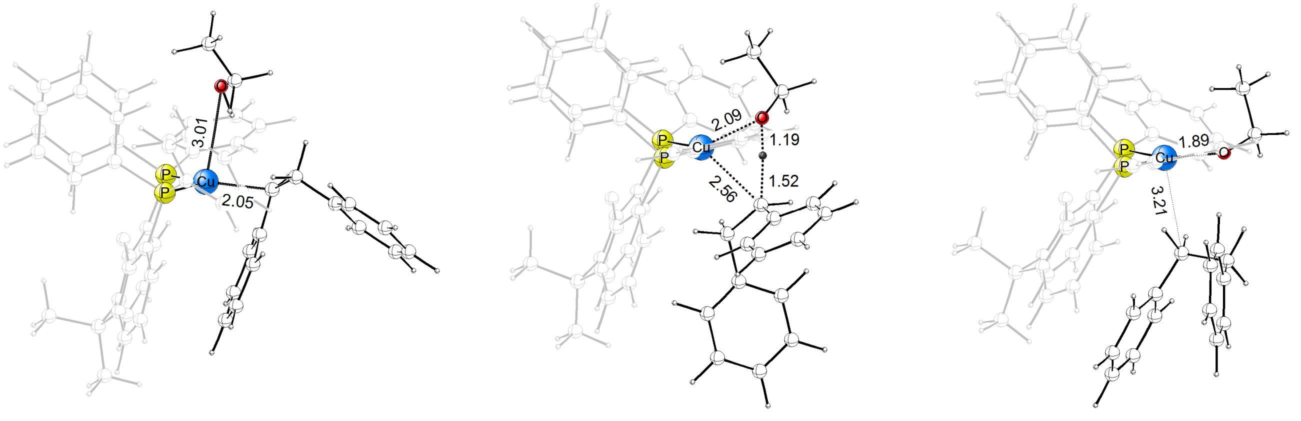

6•R　　TS[**6**•R–**5**•Q]　　**5**•Q

Figure S25. Selected structural parameter (angstrom) of the optimised structures of key stationary points for protonation of {P^P}Cu^{I} alkyl **6** by ethanol R.

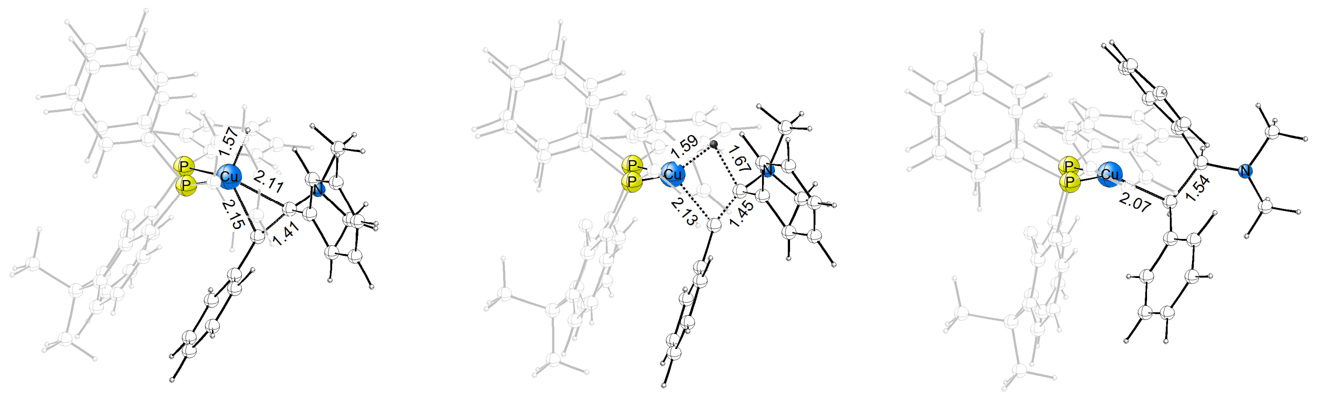

1$_2$•PEa TS[**1**$_2$•PEa–**8a**] **8a**

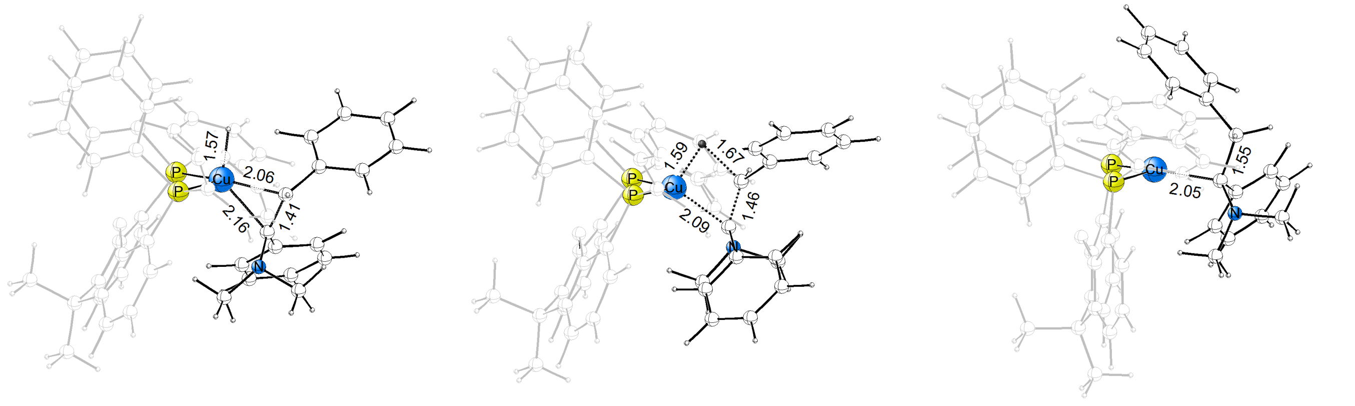

1$_2$•PEb TS[**1**$_2$•PEb–**8b**] **8b**

Figure S26. Selected structural parameter (angstrom) of the optimised structures of key stationary points for enamine C=C bond insertion into the Cu–H linkage at enamine adduct **3**•PE of the {P^P}Cu^I hydride through regioisomeric pathways.

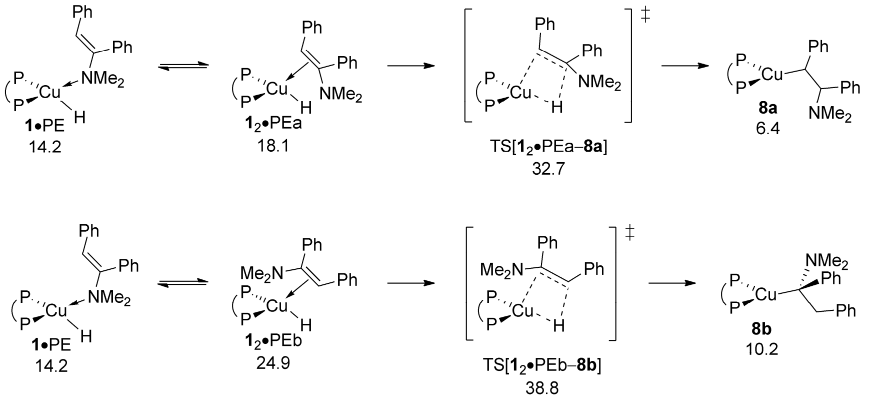

Figure S27. Regioisomeric pathways for C=C bond insertion into the Cu–H linkage at enamine adduct **1**•PE of the {P^P}Cu^I hydride compound.[16] Free energies are given in kcal mol^{-1} relative to {½**1dim** + reactants}.

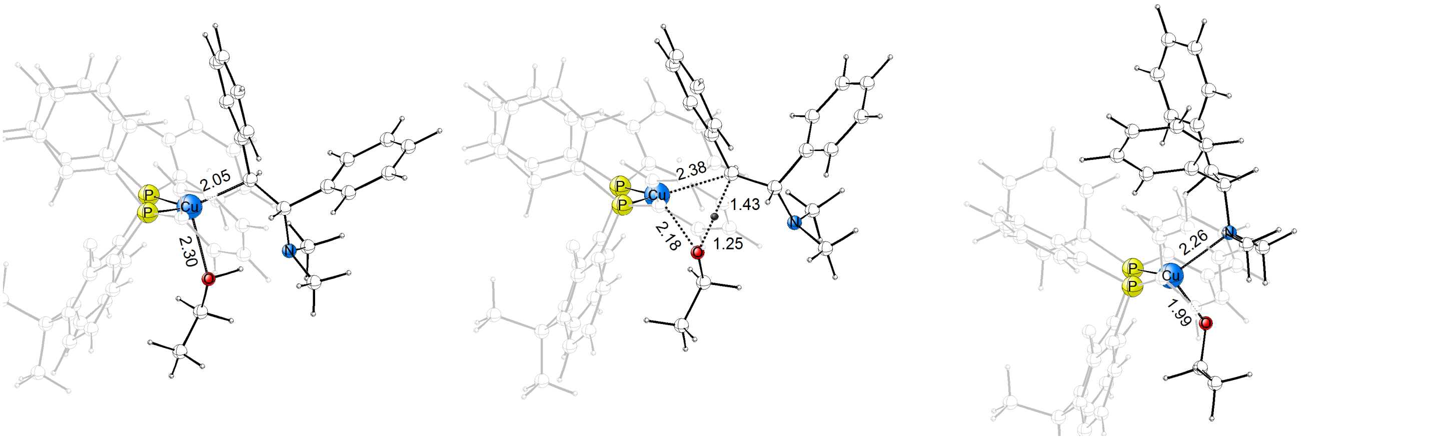

8a•R TS[**8a**•R–**5**•PA] **5**•PA

Figure S28. Selected structural parameter (angstrom) of the optimised structures of key stationary points for protonation of {P^P}Cu^{I} alkylamido **8a** by ethanol R.

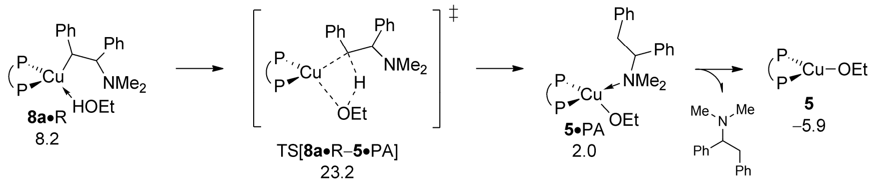

Figure S29. Protonation of the (P^P)Cu^{I} alkylamido intermediate **8a** by EtOH.[16] Free energies are given in kcal mol^{-1} relative to {½**1dim** + reactants}.

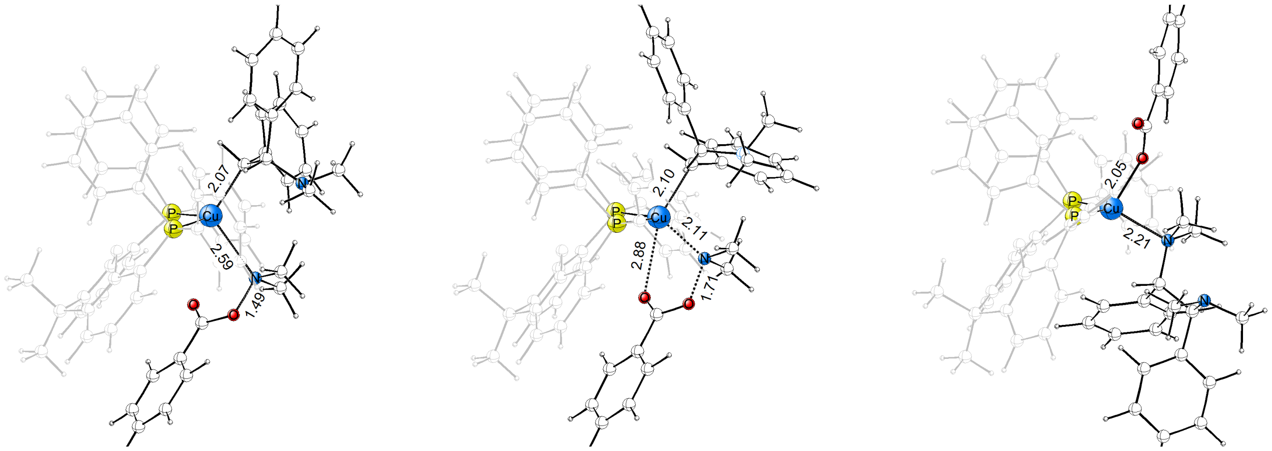

8a$_1$•A TS[**8a$_1$**•A–**9**] **4**•PD

Figure S30. Selected structural parameter (angstrom) of the optimised structures of key stationary points for S_N2 displacement of the benzoate leaving group via a multicentre TS structure at amine adduct **8a**•A of the (P^P)Cu^{I} alkylamido intermediate **8a**.

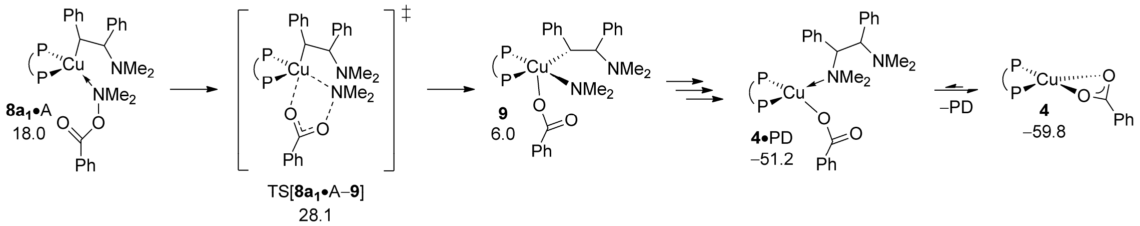

Figure S31. Cleavage of the hydroxylamine ester N–O linkage through a multicentre TS structure for S_N2 displacement of the benzoate leaving group at amine adduct **8a**•A of the (P^P)Cu^{I} alkylamido intermediate **8a.**[16] Free energies are given in kcal mol^{-1} relative to {½**1dim** + reactants}.

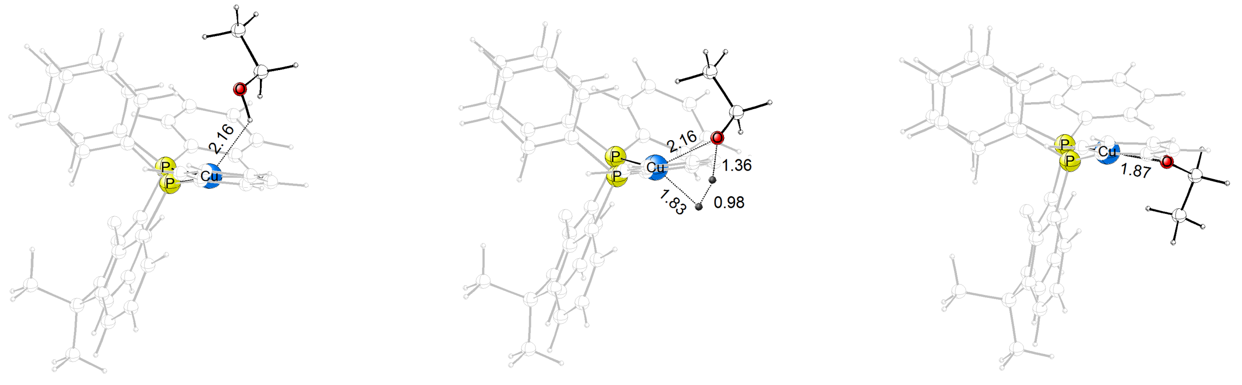

1•R TS[**1**•R–**5**] **5**

Figure S32. Selected structural parameter (angstrom) of the optimised structures of key stationary points for protonolysis of ethanol R by {P^P}Cu^I hydride.

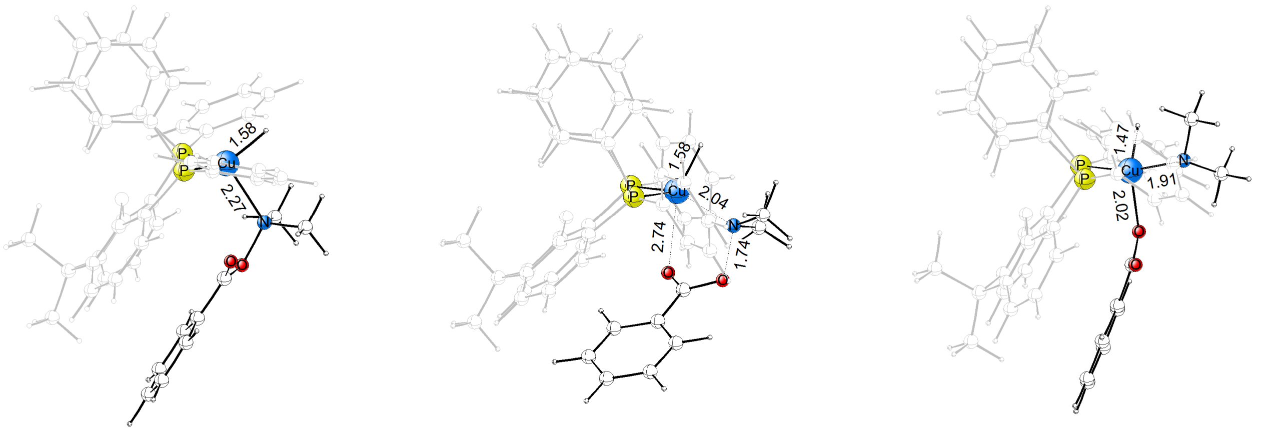

1•A TS[**1**•A–**10**] **10**

Figure S33. Selected structural parameter (angstrom) of the optimised structures of key stationary points for S_N2-type displacement of the benzoate leaving group through a multicentre TS structure at amine adduct **1**•A of the {P^P}Cu^I hydride.

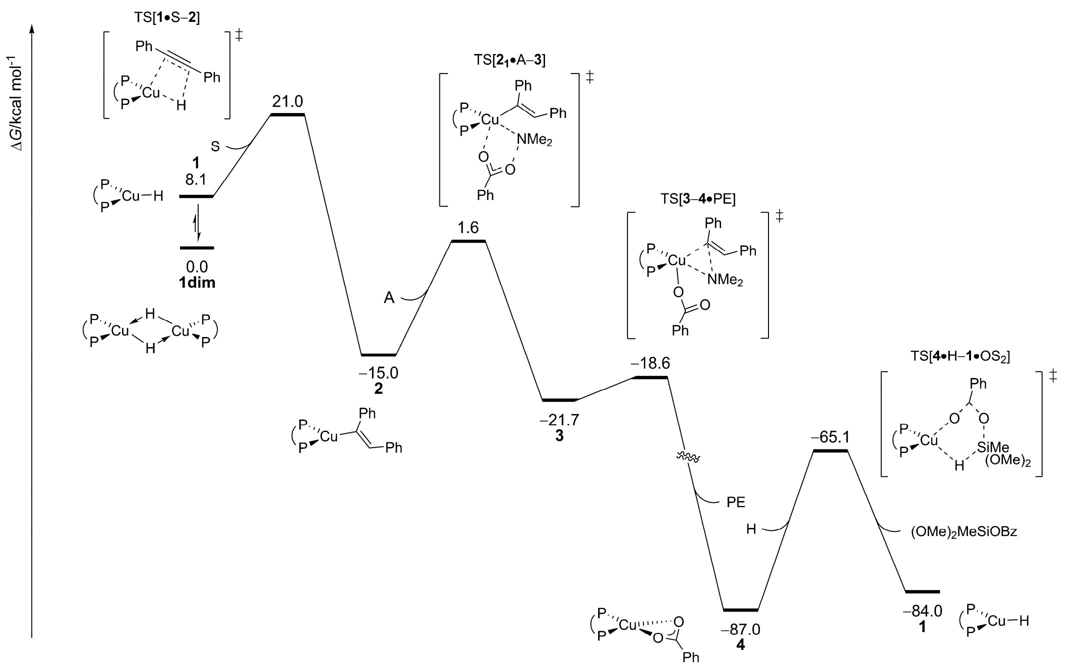

Figure S34. Condensed reaction profile for CuH-mediated direct hydroamination of 1,2-diphenylacetylene (S) with hydroxylamine ester (A) to afford (*E*)-enamine (PE), covering the most accessible pathway of all relevant steps ({P^P} = 4,5-bis(diphenylphosphino)-9,9-dimethylxanthene).

The prevalent $[\{P^{\wedge}P\}Cu(H)]_2$ dimer **1dim** of the catalytically competent Xantphos-ligated copper(I) hydride complex (i.e. ½**1dim** together with the appropriate number of reactant (S, A. H), product (PE) or solvent (T) molecules) was chosen as reference for relative free energies (given in kcal mol^{-1}).

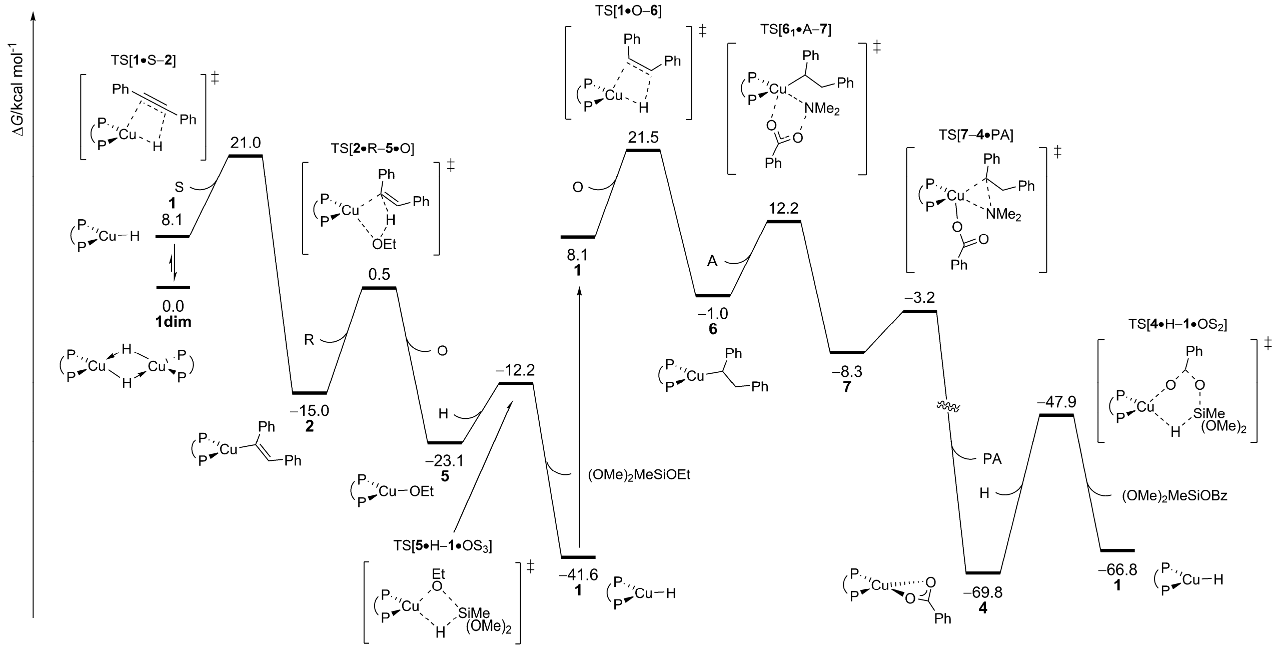

Figure S35. Condensed reaction profile for CuH-mediated reductive hydroamination of 1,2-diphenylacetylene (S) with hydroxylamine ester (A) to afford α-branched alkylamine (PA), covering the most accessible pathway of all relevant steps ({P^P} = 4,5-bis(diphenylphosphino)-9,9-dimethyl-xanthene).

The prevalent $[\{P^{\wedge}P\}Cu(H)]_2$ dimer **1dim** of the catalytically competent Xantphos-ligated copper(I) hydride complex (i.e. ½**1dim** together with the appropriate number of reactant (S, A. H, O) product (PA) or solvent (T) molecules) was chosen as reference for relative free energies (given in kcal mol^{-1}).

References and Notes

1 R. G. Parr and W. Yang, *Density-Functional Theory of Atoms and Molecules*, Oxford University Press, 1989.

2 (a) R. Ahlrichs, M. Bär, M. Häser, H. Horn and C. Kölmel, *Chem. Phys. Lett.*, 1989, **162**, 165; (b) O. Treutler and R. Ahlrichs, *J. Chem. Phys.*, 1995, **102**, 346; (c) R. Ahlrichs, F. Furche, C. Hättig, W. Klopper, M. Sierka and F. Weigend, *TURBOMOLE*, *version 6.6*; University of Karlsruhe, Karlsruhe, Germany, 2014; http://www.turbomole.com.

3 (a) P. A. M. Dirac, *Proc. R. Soc. London, Ser. A*, 1929, **123**, 714; (b) J. C. Slater, *Phys. Rev.*, 1951, **81**, 385; (c) S. Vosko, L. Wilk and M. Nusair, *Can. J. Phys.*, 1980, **58**, 1200; (d) A. D. Becke, *Phys. Rev.*, 1988, **A38**, 3096; (e) J. P. Perdew, *Phys. Rev. B: Condens. Matter*, 1986, **33**, 8822.

4 (a) O. Vahtras, J. Almlöf and M. W. Feyereisen, *Chem. Phys. Lett.*, 1993, **213**, 514; (b) K. Eichkorn, O. Treutler, H. Öhm, M. Häser and R. Ahlrichs, *Chem. Phys. Lett.*, 1995, **242**, 652.

5 (a) S. Grimme, J. Anthony, S. Ehrlich and H. Krieg, *J. Chem. Phys.*, 2010, **132**, 154104; (b) S. Grimme, S. Ehrlich and L. Goerigk, *J. Comput. Chem.*, 2011, **32**, 1456; (c) http://toc.uni-muenster.de/DFTD3/getd3.html.

6 (a) F. Weigend and R. Ahlrichs, *Phys. Chem. Chem. Phys.*, 2005, **7**, 3297; (b) F. Weigend, *Phys. Chem. Chem. Phys.*, 2006, **8**, 1057; (c) A. Schäfer, C. Huber and R. Ahlrichs, *J. Chem. Phys.*, 1992, **97**, 2571.

7 (a) Y. Zao and D. G. Truhlar, *J. Phys. Chem. A*, 2005, **109**, 5656; (b) L. Goerigk and S. Grimme, *Phys. Chem. Chem. Phys*. 2011, **13**, 6670.

8 P. Plessow, *J. Chem. Theory Comput*., 2013, **9**, 1305.

9 S.-L. Shi and S. L. Buchwald, *Nat. Chem.*, 2015, **7**, 38.

10 (a) A. Klamt and G. Schüürmann, *J. Chem. Soc. Perkin Trans. 2,* 1993, 799; (b) A. Klamt, *J. Phys. Chem*., 1995, **99**, 2224; (c) F. Eckert and A. Klamt, *AlChE J*., 2002, **48**, 369.

11 F. Eckert and A. Klamt, COSMO*therm*, version C3.0, release 15.01, COSMOlogic GmbH & Co. KG, Leverkusen, Germany, 2014.

12 W. Koch and M. C. Holthausen, *A Chemist's Guide to Density Functional Theory*, 2nd edition, Wiley-VCH, Weinheim, 2001.

13 S. Grimme, *Chem. – Eur J*., 2012, **18**, 9955.

14 For further details, see http://www.struked.de.

15 S. Tobisch, *Chem. – Eur. J.*, 2016, ***22***, 8290.

16 The prevalent $[\{P\text{^}P\}Cu^{I}(H)]_2$ dimer **1dim** of the catalytically competent Xantphos-ligated copper(I) hydride complex (i.e., ½**1dim** together with the appropriate number of reactant (S, A, H, R, O), enamine/amine product (PE, PA) and THF (T) molecules) was chosen as reference for relative free energies (given in kcal mol^{-1}).

Cartesian coordinates (in Å) of located key structures

A

E = –555.638029 a.u.

N_{imag} = 0

C	1.389450	-2.805515	-0.000080
C	0.296010	-3.676262	-0.000487
C	-1.005646	-3.165592	-0.000375
C	-1.213057	-1.788211	0.000159
C	-0.118434	-0.911081	0.000458
C	1.187328	-1.425828	0.000376
H	2.404536	-3.204015	-0.000168
H	0.458626	-4.754872	-0.000876
H	-1.859416	-3.843996	-0.000723
H	-2.217386	-1.365015	0.000385
C	-0.412677	0.553086	0.000524
H	2.034810	-0.742884	0.000688
O	0.754058	1.276260	-0.000414
O	-1.531823	1.030451	0.001227
N	0.680693	2.741701	-0.000704
C	0.047573	3.213814	1.228641
C	0.045566	3.213213	-1.229277
H	0.559992	2.755983	2.083825
H	-1.034704	3.015446	1.288205
H	0.223493	4.298472	1.268158
H	-1.037101	3.016176	-1.286329
H	0.555515	2.753576	-2.084961
H	0.222885	4.297576	-1.270572

S

E = –540.379565 a.u.

N_{imag} = 0

C	0.610441	0.000000	0.000430
C	-0.610459	-0.000000	0.000481
C	2.028335	0.000000	0.000179
C	-2.028345	-0.000000	0.000212
C	2.747739	-1.214945	-0.003279
C	4.841982	-0.000000	-0.000451
C	2.747741	1.214945	0.003522
C	4.139928	1.209305	0.003078
H	4.682501	2.155430	0.005574
H	2.196318	2.154981	0.006437
C	4.139927	-1.209306	-0.003469
H	2.196315	-2.154982	-0.005731
H	5.932351	-0.000000	-0.000727
H	4.682499	-2.155431	-0.006175
C	-2.747738	1.214938	-0.003258
C	-4.841976	0.000000	-0.000477
C	-2.747740	-1.214937	0.003545
C	-4.139920	-1.209302	0.003070
H	-4.682490	-2.155428	0.005555
H	-2.196321	-2.154965	0.006477
C	-4.139918	1.209303	-0.003476
H	-2.196317	2.154966	-0.005693
H	-5.932345	0.000000	-0.000780
H	-4.682486	2.155429	-0.006193

H

E = –410.332418 a.u.

N_{imag} = 0

Si	-0.000138	0.359690	0.000237
C	0.244576	-0.241643	-1.768759
H	-0.000095	1.860491	0.000121
C	-1.654468	-0.242016	0.672536
C	1.409798	-0.241617	1.096322
H	2.381422	0.116273	0.726494
H	1.444216	-1.341020	1.122933
H	1.291020	0.115996	2.129244
H	-1.694286	-1.341426	0.688572
H	-2.489144	0.115582	0.052580
H	-1.820976	0.115429	1.698878
H	1.199074	0.115514	-2.181404
H	-0.560782	0.116980	-2.425771
H	0.249750	-1.341049	-1.812248

R

E = –155.284333 a.u.

N_{imag} = 0

C	-0.050177	0.029863	0.050018
H	0.508394	-0.441597	0.871143
O	0.428353	1.380220	0.002392
H	0.213078	-0.511179	-0.878731
C	-1.549262	-0.059633	0.305771
H	-0.053529	1.836750	-0.708565
H	-1.815431	0.461603	1.235693
H	-1.869390	-1.109171	0.389913
H	-2.119347	0.395502	-0.519446

T

E = –232.825084 a.u.

N_{imag} = 0

O	1.213227	0.001720	0.008050
C	0.389495	0.132230	-1.167140
H	0.788196	-0.532025	-1.948670
H	0.444494	1.173211	-1.535464
C	-1.035515	-0.227023	-0.739224
H	-1.800125	0.265657	-1.353990
H	-1.193908	-1.314199	-0.801158
C	-1.043155	0.229658	0.726677
H	-1.194653	1.317904	0.787309
H	-1.818383	-0.258369	1.331809
C	0.374046	-0.139150	1.170519
H	0.765654	0.515284	1.963665
H	0.419166	-1.184240	1.529092

Q

E = –542.848919 a.u.

N_{imag} = 0

C	0.899701	-0.149724	-0.275517
C	-0.586952	-0.448394	-0.609694
H	1.478182	-0.153796	-1.212137
H	1.290567	-0.963819	0.353414
C	1.068859	1.169704	0.431820
C	-1.443337	-0.523212	0.627509
H	-0.638583	-1.398437	-1.163534
H	-0.963874	0.340379	-1.278044
C	-1.529280	-1.712293	1.365166
C	-2.909146	-0.627023	3.030340
C	-2.105823	0.612385	1.113218
C	-2.833330	0.563147	2.303447
H	-3.339100	1.458910	2.666350
H	-2.035234	1.548844	0.557197
C	-2.255412	-1.767414	2.556108
H	-1.019348	-2.606699	0.999284
H	-3.477628	-0.667544	3.960255
H	-2.314046	-2.703159	3.114007
C	1.154970	2.364394	-0.296733
C	1.220023	3.654669	1.749183
C	1.061600	1.241141	1.831570
C	1.136949	2.471421	2.486576
H	1.126370	2.505913	3.576800
H	0.979823	0.320223	2.411522
C	1.230760	3.597644	0.352990
H	1.162944	2.325550	-1.388656
H	1.279573	4.616832	2.259306
H	1.301588	4.516232	-0.231364

PE

E = –675.812182 a.u.

N_{imag} = 0

C	-1.921274	-1.605772	-1.223877
C	-2.884257	-1.479555	-0.218377
C	-2.555693	-0.838524	0.979376
C	-1.270167	-0.331643	1.171660
C	-0.293629	-0.467315	0.172709
C	-0.636073	-1.101894	-1.030195
H	-2.173353	-2.099786	-2.162987
H	-3.888707	-1.876988	-0.368840
H	-3.302417	-0.738104	1.768290
H	-1.003691	0.165523	2.105485
C	1.084643	0.047713	0.385839
C	2.195330	-0.731763	0.228606
H	0.123302	-1.206289	-1.805360
C	2.258928	-2.180009	0.042232
N	1.158012	1.378644	0.808598
C	2.445907	1.899002	1.224715
C	0.320923	2.371617	0.139732
H	3.142777	2.063082	0.377674
H	2.917367	1.201386	1.928967
H	2.290859	2.864299	1.725638
H	0.169170	3.231892	0.806540
H	-0.654995	1.940390	-0.104645
H	0.785709	2.734591	-0.797712
C	3.351829	-2.725286	-0.664595
C	2.502531	-4.972893	-0.376403
C	1.308378	-3.082945	0.564933
C	1.426779	-4.454093	0.351379
H	0.676624	-5.127452	0.769330
H	0.475839	-2.703226	1.155168
C	3.470328	-4.097441	-0.876513
H	4.111409	-2.048455	-1.062116
H	2.592502	-6.047410	-0.539037
H	4.323635	-4.486128	-1.434651
H	3.167008	-0.234548	0.240554

PA

E = –677.026145 a.u.

N_{imag} = 0

C	-1.554565	-0.702901	-2.029529
C	-2.331799	-1.661341	-1.371760
C	-2.041312	-1.994446	-0.047378
C	-0.977519	-1.375361	0.612017
C	-0.189530	-0.423495	-0.041621
C	-0.488777	-0.091071	-1.369222
H	-1.779529	-0.433608	-3.062711
H	-3.162568	-2.142860	-1.889145
H	-2.641707	-2.741140	0.473847
H	-0.739984	-1.647543	1.641986
C	1.035756	0.160326	0.635647
C	2.298795	-0.580440	0.117088
H	0.957827	-0.055223	1.728093
H	0.119646	0.662996	-1.870865
H	2.441464	-0.314275	-0.941117
C	2.172820	-2.073177	0.276091
H	3.179778	-0.231949	0.673452
N	1.117694	1.610375	0.408590
C	2.346081	2.210441	0.915584
C	-0.041223	2.287475	0.987140
H	3.221624	1.841674	0.368655
H	2.504744	2.022805	2.002180
H	2.294891	3.297485	0.767556
H	-0.084353	2.183291	2.094556
H	-0.967160	1.873566	0.570295
H	0.002897	3.357879	0.744568
C	1.730812	-2.879581	-0.780841
C	1.788550	-4.838527	0.638737
C	2.426634	-2.675923	1.515665
C	2.235592	-4.046677	1.699276
H	2.438483	-4.498135	2.671532
H	2.774963	-2.060240	2.348254
C	1.540174	-4.250313	-0.603723
H	1.512489	-2.419973	-1.746086
H	1.637824	-5.909533	0.779382
H	1.189743	-4.860227	-1.437382

O

E = –541.621859 a.u.

N_{imag} = 0

C	-0.666241	-1.934904	-0.004108
C	0.684876	-1.928340	0.013876
C	-1.596680	-0.801082	-0.091470
H	-1.149009	-2.916339	0.036915
C	1.604318	-0.785158	0.095422
H	1.177093	-2.905268	-0.022117
C	-1.320914	0.342415	-0.865465
C	-3.453559	1.315842	-0.255870
C	-2.836878	-0.872282	0.569079
C	-3.751301	0.178745	0.498321
H	-4.701932	0.106778	1.028424
H	-3.075619	-1.762433	1.154593
C	-2.237657	1.387337	-0.944319
H	-0.381019	0.401038	-1.413236
H	-4.169155	2.136362	-0.318684
H	-2.006729	2.261907	-1.554073
C	2.845201	-0.847769	-0.564703
C	3.440726	1.350332	0.249195
C	1.317458	0.359568	0.863539
C	2.224110	1.413631	0.937179
H	1.984725	2.289008	1.542500
H	0.376939	0.411963	1.410865
C	3.749487	0.212328	-0.499173
H	3.092512	-1.738495	-1.145772
H	4.148379	2.178018	0.307860
H	4.700864	0.146788	-1.028772

1
E = –3908.703407 a.u.
N_{imag} = 0

Cu	2.110026	0.694740	0.050398
P	0.790230	0.853407	-1.766906
P	0.850588	0.398237	1.899506
O	-0.498895	-1.139214	-0.171100
C	0.364073	-0.830486	-2.345146
C	0.678398	-1.362487	-3.602346
H	1.131850	-0.721510	-4.358075
C	0.436607	-2.711866	-3.869491
H	0.691163	-3.120852	-4.847592
C	-0.117668	-3.548604	-2.894979
H	-0.283482	-4.600553	-3.125382
C	-0.459072	-3.047836	-1.633073
C	-0.208964	-1.693782	-1.404456
C	-1.116630	-3.841475	-0.500169
C	-0.923948	-5.350891	-0.669114
H	0.139739	-5.625148	-0.669695
H	-1.373222	-5.693480	-1.610417
H	-1.425182	-5.897521	0.140328
C	-2.634751	-3.516578	-0.503634
H	-2.807335	-2.438348	-0.390740
H	-3.134376	-4.036338	0.326097
H	-3.087946	-3.841279	-1.450991
C	-0.520945	-3.329241	0.815447
C	-0.256832	-1.960021	0.915306
C	0.294425	-1.346236	2.047206
C	0.553021	-2.164865	3.156418
H	0.996579	-1.731464	4.051959
C	0.280464	-3.532132	3.100594
H	0.491340	-4.158243	3.967939
C	-0.238570	-4.112789	1.939034
H	-0.419887	-5.186796	1.912731
C	1.518506	1.613957	-3.261857
C	2.917785	1.638716	-3.355444
H	3.507589	1.248743	-2.521090
C	3.531251	2.171143	-4.491149
H	4.619746	2.188772	-4.560217
C	2.753865	2.691990	-5.528934
H	3.234601	3.115543	-6.411920
C	1.358696	2.680587	-5.431806
H	0.750934	3.094167	-6.237931
C	0.740571	2.141848	-4.302749
H	-0.347411	2.135124	-4.223128
C	-0.836567	1.677154	-1.635532
C	-2.033044	1.103978	-2.085203
H	-2.019647	0.116506	-2.547959
C	-3.239638	1.793715	-1.946157
H	-4.166689	1.337578	-2.296827
C	-3.259049	3.063516	-1.366835
H	-4.201568	3.601147	-1.257370
C	-2.068801	3.637985	-0.911575
H	-2.080759	4.620171	-0.439400
C	-0.867027	2.945208	-1.034090
H	0.058006	3.383453	-0.654991
C	1.705811	0.675972	3.493736
C	3.107292	0.684511	3.483464
H	3.624737	0.569705	2.526277
C	3.812988	0.854786	4.677183
H	4.903671	0.863507	4.663920
C	3.124875	1.023881	5.880674
H	3.677054	1.162827	6.811291
C	1.725624	1.023061	5.893339
H	1.186777	1.158971	6.832176
C	1.016924	0.848926	4.704945
H	-0.073940	0.852582	4.711957
C	-0.665910	1.401728	2.092212
C	-0.515670	2.749748	2.462692
H	0.479167	3.142617	2.679815
C	-1.629068	3.579842	2.577519
H	-1.498241	4.619512	2.881232
C	-2.906060	3.084056	2.300241
H	-3.777111	3.734909	2.382613
C	-3.058756	1.753787	1.905679
H	-4.050115	1.362443	1.674676
C	-1.948282	0.914777	1.805641
H	-2.083377	-0.124288	1.509691
H	3.669466	0.654788	0.041539

1•A
E = –4464.369528 a.u.
N_{imag} = 0

Cu	1.868900	1.020539	0.041477
P	0.515176	1.315410	-1.740676
P	0.569276	0.788048	1.871121
O	-1.340605	-0.079774	-0.137592
C	-0.426972	-0.148816	-2.311648
C	-0.281073	-0.838485	-3.525196
H	0.386206	-0.446109	-4.292354
C	-0.983259	-2.026254	-3.751424
H	-0.855770	-2.552379	-4.698178
C	-1.859197	-2.539579	-2.788783
H	-2.408853	-3.457407	-2.994452
C	-2.026538	-1.885247	-1.563105
C	-1.280255	-0.723303	-1.358470
C	-2.982127	-2.294932	-0.439222
C	-3.475006	-3.734868	-0.584617
H	-2.645064	-4.450921	-0.544695
H	-4.008107	-3.863904	-1.536193
H	-4.185533	-3.978936	0.216298
C	-4.203769	-1.336683	-0.476752
H	-3.891315	-0.289275	-0.377745
H	-4.890136	-1.572637	0.348719
H	-4.742372	-1.449271	-1.428535
C	-2.229109	-2.067502	0.873870
C	-1.428079	-0.924152	0.958782
C	-0.650324	-0.582464	2.070562
C	-0.732073	-1.437987	3.179637
H	-0.123332	-1.234435	4.058862
C	-1.547318	-2.569562	3.144617
H	-1.593328	-3.224577	4.015470
C	-2.280915	-2.893646	1.998798
H	-2.880874	-3.802428	1.980668
C	1.251898	2.009073	-3.276395
C	2.554402	2.518712	-3.193108
H	3.078473	2.451293	-2.234277
C	3.142367	3.132978	-4.301596
H	4.154775	3.532631	-4.226225
C	2.433262	3.241675	-5.499534
H	2.892395	3.718689	-6.366591
C	1.124055	2.754646	-5.581668
H	0.559363	2.858870	-6.509385
C	0.531793	2.151076	-4.472798
H	-0.501201	1.805195	-4.529968
C	-0.793284	2.574323	-1.481528
C	-2.129277	2.403255	-1.861863
H	-2.456741	1.457783	-2.295731
C	-3.046286	3.442083	-1.689542
H	-4.087384	3.297920	-1.982817
C	-2.631476	4.661703	-1.151712
H	-3.347816	5.473302	-1.018304
C	-1.298341	4.834799	-0.770715
H	-0.972066	5.777866	-0.332777
C	-0.384239	3.794760	-0.924378
H	0.652438	3.915264	-0.605164
C	1.441520	0.782032	3.484496
C	2.821498	0.555540	3.492499
H	3.329367	0.406378	2.539801
C	3.530822	0.563054	4.695375
H	4.607863	0.389589	4.691753
C	2.864099	0.804850	5.898345
H	3.417952	0.815566	6.838266
C	1.486816	1.050506	5.895913
H	0.965743	1.256193	6.832147
C	0.779092	1.043845	4.694250
H	-0.290924	1.257200	4.689437
C	-0.460549	2.290861	2.091110
C	0.218396	3.485624	2.386530
H	1.307705	3.479936	2.452104
C	-0.491784	4.664321	2.602124
H	0.045715	5.582053	2.845602
C	-1.886115	4.673288	2.498210
H	-2.442302	5.597585	2.659736
C	-2.562109	3.496889	2.172234
H	-3.648050	3.499816	2.071175
C	-1.854992	2.308696	1.975942
H	-2.395481	1.392384	1.742508
H	3.087089	2.020561	0.195845

1•A (cont)
E = –4464.369528 a.u.
N_{imag} = 0

C	-0.194059	-5.330404	-1.078219
C	-0.525107	-6.089478	0.049109
C	-0.116547	-5.671849	1.318890
C	0.632679	-4.506522	1.461034
C	0.980809	-3.754792	0.330509
C	0.556213	-4.164782	-0.942464
H	-0.527906	-5.645186	-2.067388
H	-1.108749	-7.004362	-0.062403
H	-0.383413	-6.256745	2.199627
H	0.958939	-4.154870	2.438630
C	1.808868	-2.538881	0.542790
H	0.802723	-3.562800	-1.814778
O	2.346821	-2.128850	-0.640461
O	2.010377	-2.008682	1.618835
N	3.041805	-0.813017	-0.615336
C	3.354019	-0.641840	-2.041793
C	4.297002	-0.967355	0.144636
H	2.423947	-0.637701	-2.617824
H	4.014724	-1.446213	-2.406978
H	3.847021	0.329431	-2.146825
H	4.922488	-1.769605	-0.282549
H	4.063564	-1.179370	1.189753
H	4.806998	0.000766	0.074658

1•S

E = –4449.112772 a.u.

N_{imag} = 0

Cu	1.357187	0.694395	0.111913
P	-0.022107	0.975348	-1.710343
P	0.027380	0.506817	1.950634
O	-1.855863	-0.398199	-0.037112
C	-1.093046	-0.402903	-2.263121
C	-1.095041	-0.987272	-3.536497
H	-0.487193	-0.549748	-4.328460
C	-1.854039	-2.135073	-3.778042
H	-1.846327	-2.587125	-4.770428
C	-2.621228	-2.714224	-2.762207
H	-3.196499	-3.614813	-2.974007
C	-2.654843	-2.150121	-1.481535
C	-1.887152	-1.003483	-1.279365
C	-3.500705	-2.651284	-0.308888
C	-3.894784	-4.121489	-0.472206
H	-3.007937	-4.766593	-0.520654
H	-4.484526	-4.263618	-1.387097
H	-4.523848	-4.449421	0.365552
C	-4.785006	-1.781019	-0.241268
H	-4.534199	-0.717818	-0.131115
H	-5.401684	-2.082747	0.617301
H	-5.372650	-1.903177	-1.162255
C	-2.698653	-2.410856	0.972941
C	-1.919449	-1.253668	1.045649
C	-1.144820	-0.894156	2.155675
C	-1.195608	-1.742820	3.271160
H	-0.598888	-1.509053	4.152152
C	-1.978137	-2.897875	3.241951
H	-2.005332	-3.551848	4.114213
C	-2.713204	-3.236033	2.101719
H	-3.299133	-4.154410	2.095668
C	0.873115	1.391129	-3.249217
C	2.147368	0.831773	-3.430542
H	2.573383	0.204602	-2.646321
C	2.870747	1.093635	-4.594725
H	3.862332	0.658299	-4.724429
C	2.333032	1.926213	-5.580136
H	2.902456	2.139237	-6.485855
C	1.069494	2.496132	-5.398504
H	0.651291	3.153379	-6.162361
C	0.339966	2.229298	-4.238482
H	-0.643701	2.677553	-4.093200
C	-1.208620	2.351704	-1.514798
C	-2.541535	2.281462	-1.940846
H	-2.911150	1.372782	-2.417468
C	-3.396782	3.369530	-1.754335
H	-4.435308	3.304107	-2.082537
C	-2.922823	4.537784	-1.154472
H	-3.591392	5.387049	-1.007278
C	-1.593821	4.610973	-0.728621
H	-1.224709	5.513737	-0.242080
C	-0.741812	3.521176	-0.895627
H	0.286236	3.550860	-0.528936
C	0.995203	0.411235	3.500089
C	2.292492	-0.115738	3.430421
H	2.700008	-0.403028	2.460087
C	3.061566	-0.246555	4.588578
H	4.071596	-0.653061	4.523966
C	2.544076	0.157823	5.821390
H	3.147676	0.063232	6.725100
C	1.255264	0.696216	5.895055
H	0.852028	1.020561	6.855480
C	0.482666	0.822137	4.740160
H	-0.520038	1.247988	4.795063
C	-0.995817	1.997366	2.209552
C	-0.326968	3.196185	2.509173
H	0.760909	3.197454	2.589930
C	-1.047055	4.373986	2.695119
H	-0.518994	5.296287	2.941664
C	-2.437637	4.378335	2.549422
H	-2.999412	5.303866	2.681045
C	-3.102401	3.196386	2.220411
H	-4.185042	3.195375	2.089121
C	-2.387548	2.007640	2.060351
H	-2.917611	1.086301	1.822185
H	1.941774	2.117962	0.346486

1•S (cont)

E = –4449.112772 a.u.

N_{imag} = 0

C	3.077764	-0.365368	-0.168840
C	2.163238	-1.229991	-0.166478
C	4.453144	0.041397	-0.277395
C	1.382303	-2.420764	-0.226083
C	5.446990	-0.938439	-0.489792
C	7.153253	0.778089	-0.520667
C	4.833287	1.391169	-0.190233
C	6.174039	1.752877	-0.310960
H	6.457050	2.804226	-0.242080
H	4.043242	2.126580	-0.026818
C	6.783845	-0.569091	-0.609546
H	5.150723	-1.985769	-0.558595
H	8.201535	1.064350	-0.615558
H	7.543714	-1.334777	-0.773012
C	1.020276	-3.104498	0.953815
C	-0.132765	-4.786265	-0.351088
C	0.958310	-2.936372	-1.468660
C	0.209668	-4.108414	-1.524381
H	-0.113568	-4.489639	-2.492925
H	1.220276	-2.404473	-2.382066
C	0.275235	-4.277315	0.885514
H	1.331580	-2.703201	1.917412
H	-0.713092	-5.708295	-0.399139
H	0.002783	-4.792095	1.806569

1•H

E = –4319.057384 a.u.

N_{imag} = 0

Cu	1.624164	0.945510	0.131965
P	0.337694	1.127224	-1.711103
P	0.307331	0.702303	1.941005
O	-1.205622	-0.721946	-0.045703
C	-0.354421	-0.488435	-2.249551
C	-0.093439	-1.075583	-3.497150
H	0.426092	-0.499943	-4.261888
C	-0.461285	-2.398086	-3.749474
H	-0.245022	-2.839940	-4.722501
C	-1.088713	-3.166198	-2.764802
H	-1.349416	-4.202204	-2.977500
C	-1.368109	-2.618871	-1.508216
C	-0.996869	-1.288469	-1.293125
C	-2.043105	-3.360313	-0.350243
C	-1.993032	-4.879052	-0.534469
H	-0.958989	-5.244158	-0.597136
H	-2.524745	-5.174482	-1.448368
H	-2.492421	-5.384487	0.302450
C	-3.524239	-2.903631	-0.271692
H	-3.595520	-1.817354	-0.131496
H	-4.025678	-3.394658	0.574340
H	-4.050898	-3.169117	-1.199191
C	-1.332982	-2.914628	0.930285
C	-0.966583	-1.571344	1.022354
C	-0.303688	-1.013238	2.123687
C	-0.013049	-1.864963	3.198010
H	0.512506	-1.470131	4.067157
C	-0.370191	-3.214370	3.141763
H	-0.132047	-3.869678	3.980004
C	-1.020613	-3.736747	2.019847
H	-1.276069	-4.795387	1.993245
C	1.198417	1.713216	-3.217239
C	2.595654	1.793837	-3.178377
H	3.104377	1.551568	-2.241026
C	3.304242	2.211345	-4.308245
H	4.392617	2.274587	-4.271760
C	2.620097	2.557332	-5.475000
H	3.173048	2.887645	-6.355586
C	1.222100	2.493267	-5.512834
H	0.686091	2.773856	-6.420693
C	0.512473	2.074298	-4.388449
H	-0.577629	2.032632	-4.412191
C	-1.092959	2.265421	-1.648867
C	-2.413919	1.840135	-1.457753
H	-2.640528	0.778414	-1.380617
C	-3.447744	2.773664	-1.372407
H	-4.470884	2.429020	-1.218459
C	-3.178351	4.138725	-1.483522
H	-3.990001	4.864407	-1.421295
C	-1.861467	4.570488	-1.663541
H	-1.639502	5.635510	-1.745154
C	-0.824330	3.642692	-1.734552
H	0.202900	3.984136	-1.872664
C	1.063538	1.032554	3.574130
C	2.459257	1.139651	3.629425
H	3.025045	1.073618	2.696047
C	3.095374	1.358239	4.853775
H	4.182303	1.441734	4.892448
C	2.340187	1.482013	6.022129
H	2.836631	1.658997	6.977356
C	0.944590	1.393064	5.967492
H	0.353844	1.502297	6.878195
C	0.306551	1.169852	4.747616
H	-0.782043	1.108995	4.700735
C	-1.220623	1.702632	2.000317
C	-1.100248	3.068208	1.698985
H	-0.126077	3.471064	1.415865
C	-2.216340	3.899738	1.749816
H	-2.113230	4.957603	1.508929
C	-3.468462	3.374294	2.080984
H	-4.343737	4.024377	2.106383
C	-3.598478	2.013140	2.361430
H	-4.575129	1.596471	2.612483
C	-2.478439	1.179654	2.326160
H	-2.581774	0.117686	2.552223
H	3.098890	1.478193	0.179822

1•H (cont)
E = –4319.057384 a.u.
N_{imag} = 0

Si	2.723290	-1.826772	-0.176026
H	1.553755	-0.860612	-0.103265
C	3.785646	-1.405545	-1.665028
C	3.672801	-1.768348	1.441954
C	1.937636	-3.524649	-0.407458
H	1.324120	-3.551777	-1.318750
H	2.714528	-4.299902	-0.493484
H	1.293829	-3.777775	0.445981
H	4.301676	-0.450270	-1.504446
H	3.164669	-1.311331	-2.567622
H	4.537055	-2.188403	-1.848636
H	2.988203	-1.889922	2.293799
H	4.181494	-0.801392	1.546764
H	4.423922	-2.571192	1.491198

3•T
E = –4141.548954 a.u.
N_{imag} = 0

Cu	1.613933	0.807286	0.110315
P	0.300858	1.076215	-1.696486
P	0.322486	0.587628	1.936442
O	-1.224581	-0.781524	-0.045047
C	-0.481322	-0.485170	-2.271920
C	-0.315748	-1.018683	-3.559592
H	0.151225	-0.413503	-4.335468
C	-0.700569	-2.330404	-3.837318
H	-0.558602	-2.732150	-4.841047
C	-1.249036	-3.140172	-2.838141
H	-1.522748	-4.168233	-3.072703
C	-1.449457	-2.641118	-1.547400
C	-1.068551	-1.317327	-1.308852
C	-2.080030	-3.419799	-0.389105
C	-2.007232	-4.933561	-0.608147
H	-0.968220	-5.278728	-0.698287
H	-2.550454	-5.219712	-1.518155
H	-2.481313	-5.467181	0.225932
C	-3.568243	-2.993023	-0.273267
H	-3.654936	-1.911155	-0.109514
H	-4.044488	-3.509740	0.572161
H	-4.107643	-3.248367	-1.196384
C	-1.362374	-2.990896	0.892721
C	-0.994117	-1.649393	1.004767
C	-0.349319	-1.104918	2.122151
C	-0.097805	-1.962249	3.202355
H	0.408033	-1.575858	4.086952
C	-0.458107	-3.309177	3.127709
H	-0.249909	-3.970444	3.969334
C	-1.073415	-3.822102	1.981110
H	-1.332055	-4.879760	1.941171
C	1.156470	1.661259	-3.208438
C	2.556317	1.613819	-3.219308
H	3.072250	1.293732	-2.308534
C	3.262674	2.012261	-4.357412
H	4.353043	1.977358	-4.358135
C	2.575009	2.468399	-5.483788
H	3.126522	2.785365	-6.370166
C	1.176781	2.532908	-5.471660
H	0.638791	2.899331	-6.347265
C	0.469177	2.131918	-4.339046
H	-0.620359	2.188769	-4.324910
C	-1.068207	2.280132	-1.563764
C	-2.397663	1.903934	-1.330255
H	-2.661674	0.850031	-1.261182
C	-3.389154	2.875678	-1.191397
H	-4.419058	2.569361	-1.005112
C	-3.068841	4.231113	-1.288670
H	-3.847912	4.987123	-1.184771
C	-1.742850	4.613582	-1.507674
H	-1.481199	5.670427	-1.577732
C	-0.746853	3.646828	-1.633267
H	0.287520	3.950164	-1.802969
C	1.134299	0.864834	3.554847
C	2.535311	0.847647	3.584528
H	3.076015	0.734772	2.639796
C	3.211735	1.013158	4.795519
H	4.302464	1.001878	4.813467
C	2.493025	1.209170	5.977148
H	3.021381	1.346330	6.921761
C	1.094485	1.244844	5.948306
H	0.533011	1.410166	6.869081
C	0.415875	1.073620	4.741671
H	-0.674535	1.107983	4.715396
C	-1.157535	1.651856	2.055614
C	-0.984991	3.022787	1.807668
H	0.000581	3.397771	1.525825
C	-2.064257	3.896664	1.912139
H	-1.920647	4.958619	1.713135
C	-3.331857	3.410540	2.245352
H	-4.178084	4.095026	2.314849
C	-3.514430	2.045352	2.471002
H	-4.503393	1.659289	2.722971
C	-2.431161	1.168389	2.380833
H	-2.573790	0.103106	2.566321
H	3.137501	1.192985	0.124809

3•T (cont)
E = –4141.548954 a.u.
N_{imag} = 0

O	1.804509	-1.675197	-0.233278
C	2.617878	-2.230094	0.818243
H	2.428331	-3.317816	0.880237
H	2.303179	-1.764768	1.758119
C	4.069756	-1.923919	0.423798
H	4.397698	-0.990202	0.896801
H	4.749133	-2.732338	0.725073
C	4.003883	-1.736658	-1.114160
H	4.294606	-0.711795	-1.375058
H	4.652783	-2.436641	-1.657075
C	2.523093	-1.963791	-1.447399
H	2.133052	-1.302307	-2.228030
H	2.324094	-3.011563	-1.740153

TS[**1**•S–**2**]

E = –4449.097654 a.u.

N_{imag} = 1

Cu	0.937065	0.966821	0.149738
P	-0.308612	1.185189	-1.693737
P	-0.260320	0.630099	1.995362
O	-1.697483	-0.746997	-0.133507
C	-0.898264	-0.426879	-2.326737
C	-0.668089	-0.934286	-3.612014
H	-0.194881	-0.301440	-4.362771
C	-1.019196	-2.252165	-3.915461
H	-0.831084	-2.642965	-4.915895
C	-1.603306	-3.078247	-2.950089
H	-1.855517	-4.106087	-3.207990
C	-1.855491	-2.602472	-1.658600
C	-1.497380	-1.280696	-1.392836
C	-2.524780	-3.392809	-0.532739
C	-2.426087	-4.904627	-0.752797
H	-1.379076	-5.232571	-0.776563
H	-2.910721	-5.190015	-1.695662
H	-2.947032	-5.445676	0.047930
C	-4.020817	-2.979029	-0.484382
H	-4.125636	-1.896285	-0.335512
H	-4.528307	-3.493998	0.343597
H	-4.517038	-3.247316	-1.427943
C	-1.873174	-2.961145	0.784441
C	-1.510793	-1.619043	0.923186
C	-0.938532	-1.076050	2.080963
C	-0.760382	-1.940331	3.171070
H	-0.305949	-1.564252	4.086715
C	-1.125347	-3.282981	3.072817
H	-0.970406	-3.946348	3.924116
C	-1.661712	-3.793086	1.887744
H	-1.914768	-4.850552	1.826036
C	0.502483	1.908230	-3.161717
C	1.889880	1.734530	-3.284016
H	2.436008	1.207274	-2.500560
C	2.565682	2.244774	-4.392696
H	3.644298	2.107342	-4.477476
C	1.863456	2.942634	-5.379746
H	2.393180	3.350260	-6.241824
C	0.483186	3.126996	-5.256849
H	-0.065314	3.677324	-6.022747
C	-0.197729	2.609671	-4.153081
H	-1.274195	2.755904	-4.053942
C	-1.844871	2.153798	-1.507716
C	-3.082220	1.733793	-2.011805
H	-3.153027	0.789350	-2.552895
C	-4.221744	2.517245	-1.816289
H	-5.183958	2.179746	-2.204343
C	-4.129149	3.728810	-1.128808
H	-5.019927	4.338705	-0.973405
C	-2.895994	4.151357	-0.624356
H	-2.823386	5.086535	-0.069593
C	-1.761618	3.362407	-0.800285
H	-0.802729	3.666323	-0.375940
C	0.651177	0.751910	3.575123
C	2.043494	0.589025	3.539020
H	2.538693	0.445752	2.577475
C	2.784994	0.623428	4.721375
H	3.867750	0.497865	4.683931
C	2.143115	0.831668	5.944808
H	2.723671	0.866417	6.867661
C	0.756052	1.004510	5.985117
H	0.253010	1.172232	6.938516
C	0.010941	0.961687	4.806017
H	-1.070900	1.096624	4.836727
C	-1.698407	1.725086	2.261569
C	-1.451053	3.048757	2.662392
H	-0.424949	3.373987	2.843556
C	-2.507058	3.940087	2.838209
H	-2.304443	4.960618	3.166416
C	-3.820024	3.531750	2.583221
H	-4.645655	4.233209	2.708270
C	-4.067842	2.226902	2.155466
H	-5.087629	1.906002	1.940298
C	-3.014358	1.323141	2.003119
H	-3.218489	0.301553	1.685082
H	2.188018	1.929083	0.291843

TS[**1**•S–**2**]

E = –4449.097654 a.u.

N_{imag} = 1

C	2.904567	0.393363	-0.030245
C	2.197575	-0.674460	-0.014780
C	4.264671	0.850959	-0.242694
C	1.922032	-2.064790	-0.058396
C	5.280930	-0.114512	-0.405149
C	6.924891	1.637398	-0.713111
C	4.605551	2.209229	-0.321630
C	5.923871	2.599094	-0.556320
H	6.171100	3.659987	-0.617685
H	3.809420	2.946789	-0.197556
C	6.595713	0.278369	-0.635254
H	5.015538	-1.171053	-0.349184
H	7.956158	1.942007	-0.895687
H	7.371855	-0.478917	-0.756238
C	1.919668	-2.835609	1.129528
C	1.324786	-4.827770	-0.118410
C	1.595316	-2.711314	-1.273438
C	1.309906	-4.072656	-1.295585
H	1.064326	-4.546042	-2.247401
H	1.561832	-2.128244	-2.193037
C	1.628771	-4.194366	1.091631
H	2.140448	-2.345441	2.077562
H	1.101871	-5.894909	-0.141729
H	1.629897	-4.765176	2.021204

2

E = –4449.159002 a.u.

N_{imag} = 0

Cu	0.557951	1.059090	0.182614
P	-0.646403	1.306514	-1.681233
P	-0.660396	0.756845	2.030255
O	-1.714061	-0.850009	-0.146412
C	-0.890143	-0.395815	-2.306916
C	-0.526183	-0.850111	-3.579943
H	-0.145157	-0.141614	-4.315123
C	-0.621203	-2.209037	-3.886004
H	-0.325862	-2.560666	-4.874806
C	-1.074801	-3.127269	-2.934126
H	-1.118273	-4.184667	-3.192208
C	-1.463957	-2.706256	-1.657744
C	-1.363396	-1.341596	-1.390201
C	-2.038277	-3.605814	-0.560343
C	-1.639543	-5.071014	-0.755919
H	-0.548714	-5.188988	-0.724844
H	-2.009105	-5.446543	-1.718915
H	-2.087016	-5.702076	0.022953
C	-3.585427	-3.482523	-0.607114
H	-3.901077	-2.439082	-0.475350
H	-4.037322	-4.085158	0.193483
H	-3.963286	-3.837365	-1.576472
C	-1.562751	-3.059511	0.789180
C	-1.447580	-1.674890	0.929996
C	-1.033198	-1.038926	2.107013
C	-0.760300	-1.850166	3.216657
H	-0.417958	-1.394984	4.145330
C	-0.885028	-3.236008	3.117676
H	-0.661639	-3.859831	3.983462
C	-1.268747	-3.835272	1.914406
H	-1.329641	-4.921187	1.854867
C	0.145305	2.151242	-3.088695
C	1.549156	2.143525	-3.129752
H	2.105417	1.680165	-2.310054
C	2.217112	2.734785	-4.202675
H	3.307354	2.723427	-4.231358
C	1.491732	3.348906	-5.227980
H	2.015644	3.817410	-6.062221
C	0.094577	3.371397	-5.180428
H	-0.471893	3.856421	-5.976649
C	-0.580085	2.771839	-4.115370
H	-1.670105	2.786438	-4.076030
C	-2.335618	1.992906	-1.613341
C	-3.450119	1.341901	-2.158242
H	-3.319773	0.392399	-2.678792
C	-4.722603	1.903758	-2.031863
H	-5.586077	1.387639	-2.454167
C	-4.888945	3.122632	-1.371930
H	-5.883287	3.559068	-1.271151
C	-3.780205	3.775282	-0.824834
H	-3.907664	4.716448	-0.290467
C	-2.513204	3.207944	-0.932898
H	-1.652362	3.699231	-0.475516
C	0.188045	1.067052	3.615956
C	1.589508	1.129067	3.592752
H	2.110620	1.038926	2.635171
C	2.300355	1.307713	4.781278
H	3.389719	1.353327	4.756054
C	1.617852	1.434802	5.993503
H	2.173775	1.579156	6.920915
C	0.220006	1.383284	6.018898
H	-0.313994	1.485374	6.964681
C	-0.494767	1.198075	4.835334
H	-1.584687	1.157806	4.853202
C	-2.269357	1.597899	2.219880
C	-2.276277	2.942227	2.631189
H	-1.338481	3.429774	2.903998
C	-3.474277	3.649666	2.711532
H	-3.467093	4.687409	3.048068
C	-4.678425	3.034409	2.357365
H	-5.614853	3.590471	2.411879
C	-4.674462	1.707610	1.923263
H	-5.608049	1.224469	1.633199
C	-3.479747	0.989861	1.858762
H	-3.489970	-0.048281	1.529578
H	3.470055	2.217850	0.195394

2 (cont)
E = –4449.159002 a.u.
N_{imag} = 0

C	3.548081	1.127805	0.090738
C	2.389879	0.423343	0.072811
C	4.943008	0.678966	-0.020177
C	2.278876	-1.032589	-0.100830
C	5.357216	-0.670912	-0.052551
C	7.686848	-0.010858	-0.244367
C	5.950497	1.664968	-0.095038
C	7.298703	1.331054	-0.207520
H	8.050301	2.120523	-0.265416
H	5.653428	2.716222	-0.064816
C	6.705504	-1.004447	-0.163498
H	4.616479	-1.463961	0.013246
H	8.740351	-0.279835	-0.331666
H	6.994575	-2.056808	-0.185274
C	2.117261	-1.893735	1.006496
C	1.938607	-3.823914	-0.448137
C	2.229117	-1.609138	-1.388609
C	2.066745	-2.982048	-1.557407
H	2.041326	-3.398536	-2.565245
H	2.331530	-0.960396	-2.259227
C	1.959646	-3.267019	0.834440
H	2.131636	-1.469280	2.011096
H	1.829312	-4.900924	-0.581262
H	1.849716	-3.907259	1.710555

2$_1$•A
E = –5004.827509 a.u.
N_{imag} = 0

Cu	0.830104	0.222014	-0.134160
P	-0.499459	0.455924	-1.983876
P	-0.447405	-0.059079	1.720363
O	-2.361294	-0.834752	-0.321435
C	-1.712071	-0.786894	-2.579241
C	-1.849143	-1.284949	-3.880752
H	-1.273647	-0.841749	-4.692472
C	-2.705798	-2.358971	-4.135171
H	-2.797757	-2.745270	-5.150820
C	-3.448869	-2.942915	-3.104619
H	-4.105887	-3.782845	-3.328552
C	-3.369857	-2.444734	-1.798492
C	-2.502845	-1.373433	-1.584987
C	-4.199653	-2.934409	-0.609950
C	-4.710212	-4.363732	-0.815314
H	-3.882102	-5.074972	-0.935785
H	-5.349256	-4.421127	-1.705813
H	-5.322754	-4.683875	0.037404
C	-5.413189	-1.978958	-0.449411
H	-5.079078	-0.942534	-0.309456
H	-6.013713	-2.271258	0.423829
H	-6.046177	-2.017649	-1.347386
C	-3.332842	-2.809014	0.645468
C	-2.458652	-1.721393	0.728773
C	-1.646573	-1.452614	1.838491
C	-1.746369	-2.327109	2.930379
H	-1.133908	-2.156311	3.814175
C	-2.604061	-3.425446	2.879742
H	-2.657674	-4.106510	3.728803
C	-3.385714	-3.667610	1.747774
H	-4.040249	-4.537787	1.727664
C	0.414977	0.900754	-3.507654
C	1.123690	-0.095896	-4.201191
H	1.033098	-1.136649	-3.890257
C	1.939188	0.231473	-5.283521
H	2.477580	-0.555979	-5.812875
C	2.072341	1.564869	-5.683164
H	2.718320	1.823844	-6.522752
C	1.378929	2.562194	-4.995016
H	1.485409	3.606234	-5.291790
C	0.554293	2.234484	-3.917295
H	0.023260	3.021369	-3.383177
C	-1.564702	1.920514	-1.711159
C	-2.857402	2.019833	-2.243654
H	-3.267683	1.195791	-2.828031
C	-3.620070	3.168074	-2.025309
H	-4.628896	3.232991	-2.435882
C	-3.091691	4.233199	-1.291613
H	-3.688263	5.130838	-1.123618
C	-1.802692	4.139338	-0.763037
H	-1.386550	4.957169	-0.175316
C	-1.047684	2.984211	-0.958443
H	-0.051334	2.893214	-0.525926
C	0.431313	-0.261262	3.322351
C	1.372649	-1.300431	3.402235
H	1.515345	-1.953729	2.542145
C	2.109568	-1.511279	4.566228
H	2.837780	-2.322780	4.607785
C	1.921751	-0.676900	5.672511
H	2.506670	-0.828400	6.580396
C	0.976299	0.348088	5.607452
H	0.815324	0.998520	6.468300
C	0.229526	0.551457	4.444385
H	-0.505438	1.353768	4.409603
C	-1.490083	1.422246	1.994978
C	-0.851869	2.629747	2.324587
H	0.231584	2.667364	2.417879
C	-1.598202	3.785500	2.541638
H	-1.079836	4.706777	2.809569
C	-2.986709	3.764958	2.391931
H	-3.569716	4.674397	2.542685
C	-3.623474	2.575970	2.032577
H	-4.705790	2.552440	1.899941
C	-2.883154	1.407054	1.847850
H	-3.397877	0.482233	1.593885
H	2.721805	1.171295	-2.220857

2$_1$•A (cont)
E = –5004.827509 a.u.
N_{imag} = 0

C	2.973818	1.795632	-1.355154
C	2.201737	1.671012	-0.243804
C	4.131574	2.660563	-1.625964
C	2.311797	2.582743	0.898074
C	4.903718	3.294837	-0.629814
C	6.351404	4.283769	-2.308283
C	4.525172	2.841706	-2.968510
C	5.611385	3.646184	-3.307989
H	5.885224	3.773723	-4.357049
H	3.949197	2.342564	-3.750491
C	5.993064	4.094644	-0.969078
H	4.648400	3.152646	0.418380
H	7.204299	4.912432	-2.567650
H	6.573572	4.572913	-0.178106
C	2.645873	2.128274	2.192587
C	2.303605	4.336685	3.134162
C	1.979313	3.951014	0.761444
C	1.964490	4.807218	1.860718
H	1.692828	5.855602	1.721999
H	1.737336	4.330892	-0.232160
C	2.656737	2.990643	3.286910
H	2.881099	1.074367	2.337914
H	2.290904	5.006768	3.994213
H	2.918928	2.601231	4.271794
C	0.160803	-5.886956	2.636187
C	-0.888054	-6.629943	2.086123
C	-1.426870	-6.269314	0.848519
C	-0.924782	-5.165105	0.164996
C	0.135410	-4.425480	0.707607
C	0.679394	-4.792819	1.947257
H	0.576083	-6.162640	3.605913
H	-1.286810	-7.490237	2.625151
H	-2.247289	-6.846306	0.420600
H	-1.336703	-4.850202	-0.793557
C	0.619067	-3.263736	-0.082340
H	1.499929	-4.217226	2.370596
O	1.832753	-2.827472	0.372253
O	0.030552	-2.783076	-1.031480
N	2.298685	-1.549026	-0.245832
C	3.474504	-1.217529	0.572237
C	2.736310	-1.856700	-1.617313
H	3.164193	-1.028148	1.603126
H	4.224299	-2.025200	0.539826
H	3.885897	-0.290965	0.156099
H	3.525875	-2.626755	-1.621672
H	1.875894	-2.182956	-2.203321
H	3.129905	-0.919217	-2.029667

TS[**2**$_1$•A–**3**]

E = –5004.804059 a.u.

N$_{imag}$ = 1

Cu	0.975168	0.127026	-0.129165
P	-0.462103	0.372248	-1.998336
P	-0.429433	-0.140751	1.709591
O	-2.248809	-1.032763	-0.365219
C	-1.555728	-0.972419	-2.605929
C	-1.625732	-1.499797	-3.901108
H	-1.058194	-1.035732	-4.706768
C	-2.408880	-2.628904	-4.157666
H	-2.446204	-3.039764	-5.167126
C	-3.151208	-3.235170	-3.139209
H	-3.754088	-4.114286	-3.365637
C	-3.139819	-2.709791	-1.841620
C	-2.331459	-1.593943	-1.622655
C	-3.990398	-3.206358	-0.670078
C	-4.459206	-4.651146	-0.865860
H	-3.612345	-5.344595	-0.954612
H	-5.074748	-4.737125	-1.770822
H	-5.085880	-4.972419	-0.023628
C	-5.234425	-2.282844	-0.562579
H	-4.935034	-1.234820	-0.431251
H	-5.852601	-2.578204	0.297179
H	-5.839011	-2.355535	-1.477960
C	-3.159653	-3.034713	0.604212
C	-2.327872	-1.914739	0.691243
C	-1.540347	-1.607790	1.805263
C	-1.607315	-2.484696	2.897254
H	-1.004125	-2.291617	3.782598
C	-2.422609	-3.614995	2.844266
H	-2.450006	-4.299135	3.692046
C	-3.189941	-3.889028	1.709768
H	-3.806957	-4.786267	1.686492
C	0.352998	0.942782	-3.538940
C	1.212553	0.059864	-4.216384
H	1.309830	-0.969064	-3.869108
C	1.944740	0.486683	-5.323125
H	2.606584	-0.212225	-5.836292
C	1.839966	1.809168	-5.765708
H	2.423577	2.148442	-6.622269
C	0.989631	2.693069	-5.098901
H	0.906499	3.728104	-5.433092
C	0.248043	2.264208	-3.995755
H	-0.407908	2.964507	-3.479954
C	-1.660740	1.719891	-1.672678
C	-2.965835	1.717358	-2.184809
H	-3.311440	0.877412	-2.788220
C	-3.825149	2.786135	-1.922586
H	-4.843247	2.771045	-2.315119
C	-3.382515	3.874791	-1.167281
H	-4.055413	4.708716	-0.962483
C	-2.081381	3.884013	-0.660797
H	-1.732028	4.718774	-0.054040
C	-1.230170	2.806191	-0.898197
H	-0.223879	2.795072	-0.481321
C	0.452401	-0.326129	3.308496
C	1.519070	-1.238575	3.330275
H	1.768184	-1.793899	2.425132
C	2.252816	-1.446164	4.497389
H	3.081432	-2.155941	4.496431
C	1.933702	-0.738128	5.660006
H	2.515489	-0.887305	6.570404
C	0.861882	0.157013	5.649863
H	0.599158	0.705215	6.555715
C	0.118698	0.357081	4.484136
H	-0.717767	1.054391	4.489297
C	-1.576977	1.259674	2.007845
C	-1.041301	2.483070	2.443043
H	0.028414	2.581397	2.613143
C	-1.873207	3.576463	2.675655
H	-1.433696	4.511170	3.025909
C	-3.246599	3.478830	2.442878
H	-3.895787	4.339816	2.607713
C	-3.781425	2.275361	1.980268
H	-4.849882	2.192588	1.777691
C	-2.956436	1.168811	1.774466
H	-3.396255	0.233886	1.433966
H	2.552690	1.442337	-2.250422

TS[**2**$_1$•A–**3**] (cont)

E = –5004.804059 a.u.

N$_{imag}$ = 1

C	2.767622	2.055828	-1.367299
C	2.093367	1.781473	-0.226860
C	3.783498	3.080260	-1.638516
C	2.110422	2.631362	0.961428
C	4.555908	3.700384	-0.636118
C	5.730645	5.019862	-2.297161
C	4.036751	3.433688	-2.978818
C	4.990227	4.396183	-3.305260
H	5.159936	4.657975	-4.351160
H	3.460527	2.942319	-3.765539
C	5.512405	4.658194	-0.963104
H	4.405330	3.420089	0.405143
H	6.479636	5.771861	-2.547753
H	6.098765	5.123546	-0.169094
C	2.465307	2.143496	2.237382
C	1.984461	4.285451	3.263795
C	1.678981	3.976140	0.878745
C	1.612433	4.784513	2.010203
H	1.272037	5.816834	1.912638
H	1.409439	4.375608	-0.099511
C	2.418410	2.958738	3.365728
H	2.765825	1.103365	2.342654
H	1.930405	4.918588	4.149551
H	2.697150	2.544341	4.335561
C	0.309648	-5.771781	2.511336
C	-0.772355	-6.399097	1.885238
C	-1.266975	-5.900211	0.677727
C	-0.681809	-4.780544	0.095882
C	0.416412	-4.164188	0.708411
C	0.912358	-4.661883	1.920926
H	0.689381	-6.157653	3.458086
H	-1.230892	-7.278288	2.339093
H	-2.116224	-6.381606	0.191662
H	-1.052858	-4.352171	-0.835294
C	1.021516	-3.005418	-0.014377
H	1.766275	-4.176534	2.392076
O	2.255776	-2.715760	0.380160
O	0.408496	-2.415926	-0.904890
N	2.626482	-1.127341	-0.178646
C	3.788610	-0.823841	0.667356
C	3.098480	-1.370266	-1.546322
H	3.492693	-0.766253	1.718406
H	4.577829	-1.580494	0.540625
H	4.176061	0.154785	0.350769
H	3.974050	-2.038019	-1.557693
H	2.282094	-1.797352	-2.131506
H	3.392232	-0.394361	-1.964093

3

E = –5004.842188 a.u.

N$_{imag}$ = 0

Cu	1.242987	0.087177	0.151683
P	-0.383931	0.382628	-1.528272
P	-0.382062	-0.155063	2.265987
O	-2.013094	-1.223166	0.113827
C	-1.235583	-1.131409	-2.103126
C	-1.203403	-1.675719	-3.392090
H	-0.661278	-1.164015	-4.184090
C	-1.850230	-2.883081	-3.655213
H	-1.797289	-3.312888	-4.655532
C	-2.561404	-3.549695	-2.653441
H	-3.048699	-4.496384	-2.881833
C	-2.658034	-3.009172	-1.368762
C	-1.972818	-1.815650	-1.131499
C	-3.519614	-3.566010	-0.235885
C	-3.828972	-5.052911	-0.424281
H	-2.908384	-5.649513	-0.454179
H	-4.382716	-5.213736	-1.358610
H	-4.467600	-5.421423	0.389234
C	-4.850147	-2.763153	-0.222202
H	-4.661254	-1.688715	-0.095360
H	-5.487704	-3.103731	0.606164
H	-5.388220	-2.909958	-1.169523
C	-2.792946	-3.284202	1.079167
C	-2.088927	-2.079938	1.190001
C	-1.413583	-1.674365	2.343625
C	-1.477909	-2.522500	3.458761
H	-0.958960	-2.244621	4.374938
C	-2.168101	-3.732448	3.383921
H	-2.200341	-4.388886	4.253926
C	-2.812806	-4.113615	2.203031
H	-3.334444	-5.069020	2.161260
C	0.279284	1.159739	-3.041197
C	1.161318	0.438284	-3.864384
H	1.412595	-0.591193	-3.607746
C	1.737220	1.050385	-4.976269
H	2.424912	0.482844	-5.604061
C	1.461191	2.388504	-5.268686
H	1.925721	2.867722	-6.131353
C	0.594508	3.110986	-4.447080
H	0.378155	4.158545	-4.659868
C	0.000799	2.500409	-3.341046
H	-0.671798	3.075150	-2.705745
C	-1.820592	1.454710	-1.115572
C	-3.097817	1.175132	-1.626704
H	-3.250661	0.303203	-2.261730
C	-4.176349	2.006856	-1.326737
H	-5.163537	1.771290	-1.726956
C	-3.993669	3.139274	-0.528761
H	-4.837876	3.790202	-0.298130
C	-2.724666	3.431351	-0.029577
H	-2.563105	4.309303	0.594419
C	-1.650114	2.589281	-0.312482
H	-0.669835	2.825654	0.084439
C	0.702089	-0.455867	3.705969
C	1.659569	-1.473918	3.530892
H	1.679310	-2.031370	2.594039
C	2.567145	-1.759462	4.548943
H	3.301618	-2.553888	4.408967
C	2.546034	-1.024954	5.740359
H	3.266930	-1.242311	6.529622
C	1.593720	-0.020128	5.919197
H	1.560997	0.545386	6.851492
C	0.669750	0.260124	4.907078
H	-0.081503	1.034841	5.060454
C	-1.522627	1.171986	2.785225
C	-0.989873	2.433211	3.110252
H	0.092846	2.572980	3.136432
C	-1.828073	3.503351	3.416679
H	-1.397271	4.469514	3.683756
C	-3.215996	3.341006	3.372976
H	-3.873406	4.180579	3.601097
C	-3.753819	2.100615	3.024861
H	-4.835347	1.968439	2.976508
C	-2.915785	1.021628	2.740091
H	-3.345166	0.053754	2.481421
H	0.972702	2.652695	1.136351

3 (cont)
E = –5004.842188 a.u.
N_{imag} = 0

C	1.591216	2.835482	0.251307
C	2.179062	1.749082	-0.299519
C	1.530827	4.223732	-0.199757
C	3.326674	1.674050	-1.182410
C	1.893812	4.647040	-1.495277
C	1.202920	6.917157	-0.990617
C	0.973441	5.180337	0.674719
C	0.818741	6.510524	0.289291
H	0.391076	7.231225	0.987856
H	0.656161	4.860183	1.669402
C	1.734239	5.974049	-1.879260
H	2.283241	3.921479	-2.206332
H	1.079425	7.955711	-1.299077
H	2.018552	6.276866	-2.888231
C	3.553549	0.561925	-2.014966
C	5.652533	1.503734	-2.770047
C	4.331906	2.667541	-1.109190
C	5.471696	2.587430	-1.902197
H	6.231241	3.367619	-1.834033
H	4.201313	3.504314	-0.423990
C	4.695261	0.487807	-2.809020
H	2.846334	-0.269948	-2.001894
H	6.549424	1.440571	-3.387690
H	4.843244	-0.382726	-3.449403
C	0.209374	-6.023608	-2.185997
C	-0.287494	-6.619829	-1.022731
C	-0.286581	-5.906990	0.180869
C	0.200448	-4.601121	0.218563
C	0.706046	-4.002424	-0.943282
C	0.710769	-4.722715	-2.143413
H	0.206124	-6.577020	-3.126435
H	-0.673890	-7.640033	-1.053729
H	-0.674743	-6.367079	1.090860
H	0.188803	-4.023214	1.141733
C	1.199527	-2.578930	-0.922560
H	1.103563	-4.232692	-3.033772
O	1.726363	-2.090048	-1.948541
O	0.999290	-1.949497	0.183757
N	2.754635	0.077281	1.359556
C	3.164605	1.125769	2.267807
C	3.897015	-0.647803	0.845960
H	2.296168	1.672977	2.652643
H	3.652004	0.643441	3.135561
H	3.882527	1.851468	1.836855
H	4.398418	-1.129230	1.707804
H	3.575849	-1.433659	0.153012
H	4.653230	-0.010888	0.346756

2$_2$•A
E = –5004.821535 a.u.
N_{imag} = 0

Cu	0.208014	0.147731	-0.319295
P	-1.119219	0.433050	-2.113597
P	-1.126873	-0.064480	1.503313
O	-2.861688	-1.199820	-0.552931
C	-1.915857	-1.101005	-2.716587
C	-1.683993	-1.717823	-3.954747
H	-1.082769	-1.203890	-4.704874
C	-2.198772	-2.990573	-4.214193
H	-2.002614	-3.464494	-5.176341
C	-2.961831	-3.664963	-3.255500
H	-3.348543	-4.658209	-3.480350
C	-3.230736	-3.074129	-2.014187
C	-2.691768	-1.806142	-1.785216
C	-4.076773	-3.686954	-0.894779
C	-4.251201	-5.197806	-1.066262
H	-3.285733	-5.721926	-1.056116
H	-4.759517	-5.421477	-2.013272
H	-4.877927	-5.606935	-0.263255
C	-5.471543	-3.006350	-0.913878
H	-5.382148	-1.918470	-0.798782
H	-6.090148	-3.392145	-0.091378
H	-5.978950	-3.212894	-1.866799
C	-3.383190	-3.332947	0.424149
C	-2.816194	-2.060066	0.532426
C	-2.116476	-1.605727	1.657945
C	-2.043446	-2.474466	2.757728
H	-1.503159	-2.161885	3.650608
C	-2.616500	-3.745440	2.692483
H	-2.543210	-4.413721	3.550900
C	-3.266633	-4.178952	1.532656
H	-3.683542	-5.184687	1.495950
C	-0.323248	1.113576	-3.616159
C	1.064680	1.303558	-3.592873
H	1.616732	1.070987	-2.679400
C	1.718889	1.835462	-4.707211
H	2.798223	1.989566	-4.676103
C	0.989809	2.181102	-5.846454
H	1.499251	2.599786	-6.715498
C	-0.399055	2.006526	-5.868788
H	-0.971452	2.291128	-6.752848
C	-1.055059	1.480488	-4.756783
H	-2.140151	1.364869	-4.765875
C	-2.543525	1.563955	-1.910027
C	-3.825384	1.279765	-2.398897
H	-4.022014	0.326918	-2.891794
C	-4.854122	2.212982	-2.256472
H	-5.850717	1.980733	-2.635058
C	-4.606585	3.440673	-1.639274
H	-5.410139	4.169947	-1.529618
C	-3.330551	3.724698	-1.146593
H	-3.133724	4.670509	-0.642600
C	-2.307272	2.787461	-1.268033
H	-1.322416	3.000027	-0.850599
C	-0.224994	-0.045070	3.095401
C	1.059323	-0.607797	3.103223
H	1.473946	-0.997825	2.173578
C	1.810365	-0.637056	4.278577
H	2.810292	-1.072606	4.267197
C	1.290230	-0.084490	5.452002
H	1.882325	-0.089859	6.368189
C	0.012083	0.482478	5.448817
H	-0.395203	0.917584	6.362601
C	-0.747904	0.496001	4.277956
H	-1.742626	0.941714	4.277536
C	-2.353601	1.274173	1.712891
C	-1.869518	2.554746	2.025916
H	-0.798467	2.721483	2.135874
C	-2.754618	3.613233	2.214694
H	-2.358477	4.596971	2.469260
C	-4.129535	3.416538	2.060267
H	-4.822319	4.248045	2.196079
C	-4.612431	2.152772	1.715374
H	-5.682569	1.994412	1.577080
C	-3.731564	1.082483	1.550631
H	-4.122951	0.096641	1.303852
H	3.227651	0.005530	-0.978999

2$_2$•A (cont)
E = –5004.821535 a.u.
N_{imag} = 0

C	3.078319	0.976393	-0.491428
C	1.820480	1.283631	-0.085029
C	4.355095	1.688245	-0.332662
C	1.512098	2.526235	0.629615
C	4.497351	3.031084	0.079485
C	6.919311	2.858830	-0.003961
C	5.539258	0.964752	-0.594109
C	6.800242	1.532793	-0.428782
H	7.693218	0.938676	-0.630820
H	5.453559	-0.074959	-0.918460
C	5.759111	3.600928	0.240808
H	3.613476	3.633937	0.270971
H	7.903048	3.311523	0.127191
H	5.836951	4.641804	0.560061
C	1.720506	2.646545	2.021094
C	0.831071	4.904828	2.019782
C	0.948366	3.632645	-0.042313
C	0.616307	4.801912	0.640787
H	0.190960	5.643567	0.090659
H	0.803900	3.567689	-1.122445
C	1.388385	3.817235	2.701904
H	2.152645	1.805920	2.563090
H	0.570484	5.818875	2.554214
H	1.563034	3.878463	3.777534
C	5.357873	-0.868285	2.592121
C	6.281420	-1.838187	2.989406
C	6.165106	-3.152844	2.522879
C	5.129795	-3.495846	1.657155
C	4.195087	-2.524901	1.265565
C	4.309756	-1.208140	1.738525
H	5.459171	0.162953	2.930288
H	7.101670	-1.568743	3.656234
H	6.889151	-3.908109	2.830360
H	5.027502	-4.508931	1.268203
C	3.124163	-2.936663	0.315424
H	3.602632	-0.448256	1.408327
O	2.169804	-1.947632	0.261011
O	3.096643	-3.977447	-0.312971
N	1.060251	-2.031168	-0.700636
C	0.214155	-3.182631	-0.359595
C	1.585199	-2.104450	-2.072309
H	-0.097023	-3.097645	0.686495
H	0.718260	-4.142560	-0.535379
H	-0.675886	-3.110527	-0.996334
H	2.113558	-3.045819	-2.277090
H	2.241235	-1.245998	-2.246943
H	0.714655	-2.016915	-2.734769

TS[**2**$_2$•A–**3**]
E = –5004.799639 a.u.
N_{imag} = 1

Cu	0.229981	-0.059915	-0.301972
P	-1.162665	0.207199	-2.084278
P	-1.146499	-0.274783	1.565369
O	-2.576955	-1.702526	-0.513163
C	-1.653009	-1.454011	-2.671249
C	-1.314076	-2.021786	-3.907253
H	-0.814290	-1.411144	-4.659212
C	-1.600309	-3.366115	-4.163054
H	-1.322632	-3.801789	-5.123045
C	-2.236433	-4.159001	-3.203415
H	-2.446599	-5.204129	-3.427273
C	-2.604671	-3.622721	-1.962289
C	-2.291695	-2.280866	-1.735723
C	-3.356390	-4.366438	-0.853919
C	-3.252459	-5.885806	-1.008982
H	-2.209290	-6.227461	-0.965876
H	-3.685460	-6.206187	-1.965508
H	-3.818419	-6.393842	-0.217482
C	-4.848918	-3.945376	-0.921393
H	-4.957178	-2.857852	-0.817287
H	-5.413784	-4.428505	-0.111783
H	-5.282918	-4.245372	-1.885712
C	-2.780547	-3.883678	0.480849
C	-2.436119	-2.533766	0.586340
C	-1.869718	-1.954275	1.728555
C	-1.700321	-2.779990	2.849401
H	-1.253727	-2.368269	3.754087
C	-2.052821	-4.129111	2.787665
H	-1.905911	-4.764530	3.661329
C	-2.572709	-4.680960	1.612124
H	-2.815012	-5.742567	1.581169
C	-0.498686	1.020002	-3.579887
C	0.843415	1.422096	-3.577788
H	1.444093	1.273468	-2.678908
C	1.387715	2.053675	-4.698943
H	2.430573	2.372211	-4.684244
C	0.594581	2.285585	-5.823978
H	1.018273	2.780853	-6.698677
C	-0.749968	1.896072	-5.825763
H	-1.373874	2.088866	-6.699605
C	-1.298060	1.270991	-4.706904
H	-2.351225	0.985969	-4.700290
C	-2.778638	1.040268	-1.881903
C	-3.973035	0.512317	-2.391493
H	-3.972759	-0.459367	-2.886192
C	-5.165209	1.228175	-2.269017
H	-6.090786	0.807305	-2.664560
C	-5.171207	2.480188	-1.651111
H	-6.102562	3.040001	-1.558117
C	-3.983728	3.005260	-1.136276
H	-3.982687	3.970562	-0.631081
C	-2.796155	2.284219	-1.237457
H	-1.880515	2.683093	-0.802075
C	-0.254927	-0.071955	3.146750
C	1.106840	-0.410687	3.153866
H	1.586687	-0.760284	2.237691
C	1.857492	-0.273572	4.322624
H	2.917132	-0.532473	4.311448
C	1.258148	0.216869	5.485656
H	1.848261	0.340319	6.394834
C	-0.099046	0.554409	5.482231
H	-0.569802	0.937328	6.388857
C	-0.856943	0.405327	4.319755
H	-1.912992	0.675194	4.318368
C	-2.590630	0.832111	1.746327
C	-2.354866	2.180634	2.059080
H	-1.335033	2.540162	2.186155
C	-3.421056	3.060623	2.229015
H	-3.215333	4.100712	2.484933
C	-4.734693	2.615997	2.058219
H	-5.569675	3.306733	2.182315
C	-4.972939	1.284187	1.713380
H	-5.993958	0.931551	1.563138
C	-3.909079	0.393027	1.565608
H	-4.111081	-0.648521	1.320277
H	3.012707	0.423102	-0.933018

TS[**2**$_2$•A–**3**] (cont)
E = –5004.799639 a.u.
N_{imag} = 1

C	2.722108	1.385715	-0.498306
C	1.448496	1.517621	-0.067121
C	3.865032	2.309332	-0.429355
C	0.926098	2.698449	0.608713
C	3.796073	3.663040	-0.038934
C	6.200741	3.903981	-0.275436
C	5.134913	1.789962	-0.758674
C	6.286522	2.568873	-0.678522
H	7.254692	2.129875	-0.924080
H	5.209709	0.742621	-1.057969
C	4.948489	4.443545	0.036293
H	2.835447	4.108575	0.206976
H	7.098760	4.519550	-0.208243
H	4.866928	5.487999	0.342418
C	1.123086	2.889460	1.994205
C	-0.107521	4.979121	1.932734
C	0.196021	3.681445	-0.092549
C	-0.311787	4.804045	0.559549
H	-0.864274	5.553680	-0.009912
H	0.067246	3.565864	-1.170103
C	0.618425	4.015558	2.642396
H	1.687582	2.143766	2.552597
H	-0.504183	5.857753	2.442242
H	0.789983	4.139043	3.712888
C	7.011253	-1.608531	2.020998
C	6.834624	-0.352343	2.611434
C	5.619634	0.322865	2.467894
C	4.579890	-0.257881	1.742729
C	4.756973	-1.513463	1.144275
C	5.977159	-2.186998	1.285768
H	7.961037	-2.134370	2.129823
H	7.649138	0.103476	3.176439
H	5.486238	1.313383	2.904001
H	3.637781	0.269787	1.602590
C	3.667805	-2.158497	0.316400
H	6.092735	-3.156985	0.801722
O	2.523148	-1.532282	0.470178
O	3.881610	-3.145899	-0.395239
N	1.151254	-1.779554	-0.638428
C	0.748641	-3.072022	-0.108949
C	1.768363	-1.909346	-1.951224
H	0.466018	-2.977826	0.943037
H	1.540444	-3.824028	-0.230727
H	-0.142281	-3.391072	-0.679423
H	2.526225	-2.703485	-1.970684
H	2.206391	-0.953520	-2.258129
H	0.958750	-2.158291	-2.660796

TS_{OA}[**2**$_2$•A–**3**]
E = –5004.786123 a.u.
N_{imag} = 1

Cu	1.089725	0.266445	0.028602
P	-0.416320	0.531901	-1.754603
P	-0.427286	-0.002985	1.969937
O	-1.995210	-1.209924	-0.115307
C	-1.307809	-0.956656	-2.350604
C	-1.241275	-1.456018	-3.655637
H	-0.711211	-0.897169	-4.424675
C	-1.800325	-2.701855	-3.954332
H	-1.715835	-3.098961	-4.966054
C	-2.420646	-3.463385	-2.962533
H	-2.806174	-4.451487	-3.211132
C	-2.529989	-2.981542	-1.651774
C	-1.974772	-1.729634	-1.394378
C	-3.214176	-3.711668	-0.493064
C	-3.326338	-5.218181	-0.751077
H	-2.341115	-5.682784	-0.883868
H	-3.929655	-5.409059	-1.648693
H	-3.839388	-5.716307	0.082681
C	-4.637784	-3.114312	-0.327540
H	-4.591843	-2.032522	-0.144781
H	-5.153052	-3.586737	0.520962
H	-5.225916	-3.283915	-1.240824
C	-2.424167	-3.399542	0.783066
C	-1.872406	-2.122407	0.913926
C	-1.175246	-1.680765	2.046764
C	-0.993654	-2.601817	3.087634
H	-0.423222	-2.318702	3.971240
C	-1.525133	-3.890177	2.987273
H	-1.376382	-4.598050	3.803513
C	-2.238946	-4.282202	1.851823
H	-2.635222	-5.295114	1.793273
C	0.382918	1.136626	-3.282213
C	1.513847	0.436856	-3.736210
H	1.824573	-0.474630	-3.218507
C	2.190432	0.875418	-4.872995
H	3.069996	0.331356	-5.218837
C	1.749645	2.008182	-5.564267
H	2.289016	2.354635	-6.447282
C	0.615991	2.694939	-5.123755
H	0.263419	3.574035	-5.665644
C	-0.067639	2.262591	-3.985118
H	-0.947651	2.805299	-3.638713
C	-1.761164	1.735500	-1.446404
C	-3.042790	1.560433	-1.990233
H	-3.258233	0.677562	-2.592589
C	-4.039165	2.511226	-1.761048
H	-5.033976	2.364509	-2.184696
C	-3.765466	3.645254	-0.992816
H	-4.548031	4.382673	-0.808481
C	-2.493834	3.820807	-0.442943
H	-2.280361	4.688796	0.180935
C	-1.498913	2.868476	-0.660939
H	-0.516560	2.991875	-0.205947
C	0.436792	0.167422	3.582545
C	1.806045	0.470611	3.589752
H	2.333279	0.584313	2.642848
C	2.483700	0.667344	4.795579
H	3.545078	0.918028	4.785591
C	1.799871	0.558674	6.007343
H	2.326985	0.715520	6.949538
C	0.432860	0.260648	6.011452
H	-0.107460	0.184079	6.955913
C	-0.245517	0.072725	4.808067
H	-1.316887	-0.129965	4.813695
C	-1.857742	1.139248	2.237816
C	-1.679936	2.298818	3.012109
H	-0.732646	2.475657	3.518739
C	-2.714825	3.219712	3.177963
H	-2.548563	4.109285	3.787078
C	-3.954942	2.998668	2.578013
H	-4.765922	3.716656	2.706079
C	-4.143762	1.850630	1.807279
H	-5.102977	1.668521	1.321730
C	-3.108597	0.933356	1.632019
H	-3.277986	0.064240	1.004982
H	0.732315	2.430842	2.003026

TS$_{OA}$[**2**$_2$•A–**3**] (cont)
E = –5004.786123 a.u.
N_{imag} = 1

C	1.482583	2.837431	1.322424
C	1.782474	2.128936	0.221604
C	2.050017	4.104041	1.807254
C	2.713000	2.461981	-0.835155
C	2.950651	4.911252	1.083746
C	3.064575	6.501364	2.913651
C	1.675376	4.531842	3.097697
C	2.174714	5.711689	3.645937
H	1.869202	6.015765	4.648296
H	0.989251	3.912995	3.678713
C	3.446536	6.092017	1.632059
H	3.261854	4.615762	0.084693
H	3.457885	7.426624	3.336299
H	4.140370	6.700568	1.050056
C	4.058260	2.030638	-0.787017
C	4.535671	3.176309	-2.865211
C	2.302326	3.249209	-1.934296
C	3.203626	3.604651	-2.929095
H	2.866045	4.217957	-3.763904
H	1.271862	3.597098	-1.975762
C	4.959466	2.397573	-1.786694
H	4.389825	1.447248	0.071693
H	5.237483	3.450007	-3.653424
H	5.997854	2.069298	-1.722830
C	0.215749	-6.237573	0.245743
C	0.100631	-7.110757	-0.840804
C	0.290589	-6.634820	-2.143428
C	0.613593	-5.295276	-2.356710
C	0.712074	-4.418160	-1.274167
C	0.503031	-4.891458	0.026879
H	0.063496	-6.602010	1.263093
H	-0.139624	-8.161183	-0.673510
H	0.193226	-7.312317	-2.992615
H	0.785468	-4.892725	-3.355151
C	0.996119	-2.954447	-1.489313
H	0.552740	-4.179730	0.850707
O	0.594960	-2.193449	-0.565364
O	1.612710	-2.605238	-2.544047
N	2.529485	-0.947876	0.203984
C	3.449047	-1.114688	-0.867211
C	2.785035	-1.806254	1.334451
H	2.930210	-1.727237	-1.662740
H	4.390235	-1.618047	-0.582059
H	3.661900	-0.150625	-1.360066
H	3.022449	-2.828472	0.988871
H	1.910682	-1.863182	1.992009
H	3.657201	-1.459859	1.924414

TS[**3**–**4**•PE]
E = –5004.836764 a.u.
N_{imag} = 1

Cu	1.336574	0.133100	0.003474
P	-0.203980	0.354276	-2.099715
P	-0.168171	-0.220215	1.795064
O	-1.803885	-1.317624	-0.346055
C	-1.079603	-1.186097	-2.597992
C	-1.097332	-1.707944	-3.897629
H	-0.557735	-1.193834	-4.692152
C	-1.774331	-2.897423	-4.170378
H	-1.770647	-3.301037	-5.183417
C	-2.450445	-3.577661	-3.154069
H	-2.955738	-4.515598	-3.380196
C	-2.479066	-3.069318	-1.852712
C	-1.800638	-1.869569	-1.615812
C	-3.218719	-3.707071	-0.677683
C	-3.506091	-5.192661	-0.906106
H	-2.577153	-5.763823	-1.030766
H	-4.132150	-5.331891	-1.797136
H	-4.064414	-5.609480	-0.057484
C	-4.561893	-2.950314	-0.489786
H	-4.391239	-1.877065	-0.333950
H	-5.099878	-3.349006	0.382047
H	-5.190404	-3.071981	-1.383243
C	-2.370603	-3.469172	0.571593
C	-1.736594	-2.228252	0.691817
C	-1.017604	-1.836266	1.826678
C	-0.916438	-2.752915	2.881993
H	-0.348358	-2.486507	3.771292
C	-1.504829	-4.011555	2.774579
H	-1.395119	-4.727865	3.588734
C	-2.224521	-4.367671	1.630651
H	-2.667797	-5.359894	1.564057
C	1.047719	0.551048	-3.412131
C	1.383393	1.816689	-3.919279
H	0.818347	2.695733	-3.612396
C	2.462341	1.965004	-4.792490
H	2.712179	2.956944	-5.171331
C	3.221062	0.855090	-5.170294
H	4.060386	0.971320	-5.857035
C	2.909031	-0.403668	-4.647668
H	3.510582	-1.272632	-4.918122
C	1.841313	-0.555719	-3.764200
H	1.634441	-1.528816	-3.318862
C	-1.566619	1.515162	-2.536256
C	-1.789702	2.023638	-3.822730
H	-1.129156	1.742920	-4.642753
C	-2.851877	2.898529	-4.059163
H	-3.009674	3.297004	-5.062495
C	-3.711528	3.262510	-3.018844
H	-4.532588	3.956328	-3.203611
C	-3.518155	2.726542	-1.742868
H	-4.183814	2.991754	-0.921284
C	-2.455097	1.856658	-1.505491
H	-2.306255	1.444389	-0.507584
C	0.612034	-0.129776	3.453604
C	1.771598	-0.891532	3.683837
H	2.149778	-1.531299	2.882674
C	2.398259	-0.851541	4.929269
H	3.295003	-1.450047	5.096375
C	1.883638	-0.052516	5.954959
H	2.380737	-0.017697	6.925415
C	0.731224	0.701936	5.729463
H	0.324684	1.333111	6.520220
C	0.096284	0.664547	4.486396
H	-0.798662	1.262336	4.323298
C	-1.535877	0.987853	1.924390
C	-1.222986	2.354349	2.029045
H	-0.184134	2.671526	2.089584
C	-2.234017	3.312432	2.069448
H	-1.965640	4.366278	2.149911
C	-3.574312	2.922465	2.004823
H	-4.365903	3.672213	2.031197
C	-3.894713	1.565663	1.917815
H	-4.937713	1.249355	1.878153
C	-2.883853	0.603194	1.884082
H	-3.145529	-0.452325	1.827419
H	2.636126	1.249588	2.391965

TS[**3**–**4**•PE] (cont)
E = –5004.836764 a.u.
N_{imag} = 1

C	2.308351	2.138212	1.847049
C	1.885203	1.967971	0.576715
C	2.200877	3.313210	2.726913
C	1.585063	3.023101	-0.392065
C	1.667797	4.571296	2.364622
C	1.782031	5.340502	4.667360
C	2.534300	3.117726	4.085296
C	2.328241	4.109814	5.040392
H	2.579810	3.915415	6.084359
H	2.928899	2.148337	4.391769
C	1.462912	5.563516	3.324137
H	1.395202	4.772911	1.330694
H	1.602323	6.115025	5.414728
H	1.039525	6.523166	3.021942
C	2.633673	3.777847	-0.961196
C	1.052933	5.070732	-2.266619
C	0.267721	3.341453	-0.770064
C	0.000685	4.356581	-1.685426
H	-1.033361	4.571815	-1.958892
H	-0.555713	2.801159	-0.316029
C	2.368919	4.779142	-1.894627
H	3.656543	3.572475	-0.651834
H	0.849454	5.848609	-3.003927
H	3.196952	5.337290	-2.333689
C	0.578737	-6.473970	0.496179
C	0.078190	-6.772420	-0.774860
C	0.034844	-5.775994	-1.755269
C	0.472738	-4.483362	-1.462095
C	0.981993	-4.181432	-0.192920
C	1.039193	-5.188784	0.778827
H	0.610044	-7.246536	1.266498
H	-0.275947	-7.780009	-1.000606
H	-0.354713	-6.004695	-2.748517
H	0.413952	-3.693983	-2.210452
C	1.424721	-2.785027	0.181943
H	1.438252	-4.928184	1.758370
O	2.077393	-2.628559	1.236464
O	1.038991	-1.846915	-0.611528
N	3.212697	0.239734	-0.433220
C	3.861446	1.076819	-1.405155
C	4.080459	-0.081620	0.675751
H	4.465150	0.430747	-2.072793
H	4.549139	1.823497	-0.959220
H	3.139464	1.593411	-2.045120
H	4.787742	-0.868792	0.343623
H	3.514194	-0.528917	1.498418
H	4.674287	0.781644	1.039589

4•PE

E = –5004.944128 a.u.

N_{imag} = 0

Cu	0.854482	-0.033201	-0.163268
P	-0.233704	0.271060	-2.102360
P	-0.200799	-0.285322	1.823370
O	-1.748257	-1.470539	-0.433985
C	-0.913543	-1.344092	-2.648203
C	-0.785262	-1.920157	-3.917514
H	-0.257951	-1.382400	-4.703555
C	-1.304210	-3.190552	-4.166227
H	-1.171565	-3.643189	-5.149083
C	-1.989244	-3.891183	-3.169760
H	-2.381237	-4.883813	-3.385507
C	-2.181087	-3.326000	-1.906613
C	-1.616681	-2.069367	-1.677555
C	-3.032860	-3.927843	-0.789610
C	-3.232450	-5.435154	-0.963559
H	-2.272995	-5.967513	-0.958449
H	-3.744968	-5.644791	-1.911532
H	-3.869088	-5.836815	-0.164303
C	-4.418512	-3.224991	-0.823367
H	-4.314499	-2.138554	-0.705536
H	-5.052963	-3.604717	-0.009732
H	-4.915340	-3.421558	-1.783897
C	-2.364714	-3.576681	0.539800
C	-1.759321	-2.318011	0.653055
C	-1.138682	-1.866405	1.825997
C	-1.175986	-2.712463	2.944493
H	-0.702045	-2.393412	3.871896
C	-1.776100	-3.968026	2.864325
H	-1.785748	-4.619521	3.738432
C	-2.354632	-4.399945	1.667545
H	-2.802708	-5.391445	1.616183
C	0.698944	0.939396	-3.520536
C	1.673609	0.153049	-4.160220
H	1.813465	-0.884940	-3.857298
C	2.519729	0.724724	-5.110529
H	3.271888	0.102574	-5.597674
C	2.429589	2.085599	-5.416587
H	3.104248	2.529884	-6.149586
C	1.479178	2.875672	-4.765962
H	1.408913	3.942909	-4.979884
C	0.615230	2.307083	-3.830107
H	-0.108981	2.938250	-3.319402
C	-1.793219	1.246511	-2.102785
C	-2.435612	1.612848	-3.295156
H	-1.993254	1.343738	-4.254902
C	-3.633615	2.326249	-3.257361
H	-4.120979	2.614048	-4.190018
C	-4.212623	2.666004	-2.029709
H	-5.148595	3.225755	-2.004099
C	-3.593083	2.278499	-0.839681
H	-4.034768	2.524760	0.126388
C	-2.390079	1.572629	-0.880901
H	-1.911174	1.266740	0.046015
C	0.924085	-0.550829	3.244446
C	1.904712	-1.549469	3.089563
H	1.942898	-2.115849	2.155505
C	2.813430	-1.800966	4.115343
H	3.568109	-2.578243	3.987417
C	2.764969	-1.056203	5.299652
H	3.481389	-1.250699	6.098717
C	1.793415	-0.066517	5.454641
H	1.745301	0.515771	6.375830
C	0.871988	0.184827	4.433738
H	0.115965	0.957607	4.567553
C	-1.403936	0.944686	2.430408
C	-0.953546	2.260299	2.637105
H	0.101529	2.501217	2.513197
C	-1.845867	3.265426	3.003339
H	-1.469214	4.275604	3.166935
C	-3.205654	2.973321	3.149729
H	-3.908286	3.760457	3.426064
C	-3.661702	1.668807	2.941764
H	-4.721057	1.434869	3.057059
C	-2.766887	0.655594	2.589395
H	-3.126498	-0.361106	2.429030
H	3.262781	1.638344	2.441476

4•PE (cont)

E = –5004.944128 a.u.

N_{imag} = 0

C	2.745928	2.436492	1.909199
C	2.504346	2.236648	0.585406
C	2.311633	3.512638	2.797768
C	1.812754	3.249960	-0.262642
C	1.705887	4.731958	2.416428
C	1.411005	5.381523	4.740115
C	2.478166	3.285027	4.183293
C	2.032318	4.194551	5.137552
H	2.169442	3.975230	6.197619
H	2.947946	2.353820	4.503256
C	1.263426	5.642293	3.374859
H	1.574291	4.975867	1.366670
H	1.056718	6.097541	5.482271
H	0.798339	6.573069	3.045646
C	0.415988	3.265153	-0.341085
C	0.482715	5.252941	-1.712513
C	2.541004	4.242033	-0.934376
C	1.879906	5.234457	-1.659236
H	2.457462	6.001447	-2.176432
H	3.629723	4.241310	-0.870351
C	-0.249227	4.262741	-1.054343
H	-0.147930	2.498425	0.184621
H	-0.032840	6.035623	-2.270486
H	-1.338838	4.254756	-1.096195
C	0.972632	-6.007162	-2.632043
C	0.432307	-6.665344	-1.523023
C	0.329170	-5.996123	-0.299114
C	0.756768	-4.673630	-0.187898
C	1.311734	-4.013917	-1.292750
C	1.417334	-4.690466	-2.513763
H	1.047944	-6.524751	-3.589781
H	0.092243	-7.698581	-1.612282
H	-0.093728	-6.503791	0.569351
H	0.661843	-4.129430	0.750898
C	1.756322	-2.574937	-1.189742
H	1.845530	-4.154434	-3.360383
O	2.341468	-2.039576	-2.155955
O	1.459100	-1.992139	-0.077093
N	2.827233	1.009020	-0.066760
C	3.702768	0.089549	0.677248
C	3.334713	1.134438	-1.452963
H	3.260148	-0.174093	1.640638
H	3.798315	-0.826096	0.086033
H	4.696198	0.545064	0.841453
H	4.365539	1.531672	-1.450692
H	3.314147	0.135066	-1.903002
H	2.696047	1.801825	-2.034815

4

E = –4329.098749 a.u.

N_{imag} = 0

Cu	1.234153	0.075534	-0.429170
P	0.101188	0.348547	-2.272940
P	0.058357	-0.142026	1.490903
O	-1.779897	-1.032936	-0.582228
C	-0.925345	-1.076420	-2.786431
C	-0.871846	-1.691710	-4.043882
H	-0.232931	-1.270902	-4.819846
C	-1.613711	-2.849132	-4.289582
H	-1.558965	-3.326799	-5.268081
C	-2.422766	-3.404324	-3.293483
H	-2.989159	-4.309902	-3.508201
C	-2.514837	-2.807620	-2.030471
C	-1.755458	-1.654600	-1.816631
C	-3.416451	-3.280318	-0.886964
C	-3.831774	-4.745084	-1.048088
H	-2.960612	-5.414093	-1.057867
H	-4.391766	-4.886902	-1.981603
H	-4.496818	-5.049963	-0.229664
C	-4.686825	-2.387314	-0.878743
H	-4.422828	-1.327403	-0.769228
H	-5.341279	-2.669738	-0.041963
H	-5.240752	-2.510169	-1.820239
C	-2.655201	-3.037849	0.418970
C	-1.872755	-1.882599	0.505843
C	-1.123384	-1.531114	1.636164
C	-1.213844	-2.368871	2.757092
H	-0.639265	-2.127560	3.651137
C	-2.001132	-3.519922	2.711447
H	-2.059279	-4.168830	3.585649
C	-2.703704	-3.859315	1.550493
H	-3.296012	-4.773602	1.532432
C	0.999472	0.787906	-3.800608
C	2.359135	0.452695	-3.870490
H	2.836177	-0.021455	-3.009481
C	3.093711	0.748118	-5.020713
H	4.151736	0.488154	-5.069707
C	2.477701	1.390196	-6.097656
H	3.054205	1.629395	-6.992246
C	1.125430	1.740549	-6.024792
H	0.647701	2.253113	-6.860901
C	0.386636	1.441145	-4.880086
H	-0.665448	1.723109	-4.816049
C	-1.112845	1.697139	-2.069708
C	-2.433860	1.617529	-2.527953
H	-2.778766	0.716618	-3.037180
C	-3.311188	2.684121	-2.321657
H	-4.341868	2.613322	-2.672316
C	-2.870136	3.836650	-1.668238
H	-3.557020	4.667621	-1.503265
C	-1.552089	3.918613	-1.211182
H	-1.209817	4.808277	-0.683703
C	-0.677508	2.850365	-1.398851
H	0.341473	2.895210	-1.008435
C	1.078962	-0.294599	2.996343
C	2.201131	-1.135870	2.922884
H	2.417291	-1.664489	1.993872
C	3.069170	-1.247011	4.008505
H	3.954789	-1.877139	3.928810
C	2.832247	-0.512503	5.171750
H	3.528535	-0.576918	6.008483
C	1.711822	0.318788	5.254641
H	1.522927	0.893656	6.162407
C	0.834749	0.425788	4.174421
H	-0.031366	1.084385	4.237953
C	-0.942382	1.359544	1.760483
C	-0.263721	2.572615	1.976317
H	0.827493	2.582885	1.971594
C	-0.986114	3.748618	2.168265
H	-0.452588	4.682988	2.349322
C	-2.382919	3.738732	2.106627
H	-2.944278	4.664004	2.242577
C	-3.056243	2.542381	1.854880
H	-4.144939	2.529312	1.790821
C	-2.341229	1.354022	1.693366
H	-2.875328	0.419715	1.520898
C	6.486715	-0.083026	2.625580

4 (cont)
E = –4329.098749 a.u.
N_{imag} = 0

C	6.480588	0.887467	3.633035
C	5.480745	1.864413	3.653202
C	4.490614	1.870458	2.671582
C	4.482489	0.890904	1.670490
C	5.488328	-0.085128	1.652206
H	7.268449	-0.844142	2.606459
H	7.255093	0.881918	4.401633
H	5.471459	2.619340	4.440831
H	3.694122	2.613826	2.672858
C	3.341817	0.831658	0.701003
H	5.455736	-0.849114	0.875742
O	3.213426	-0.234491	-0.007541
O	2.494477	1.771559	0.668089

4•S
E = –4869.507212 a.u.
N_{imag} = 0

Cu	0.955961	0.598341	0.175645
P	-0.332771	0.839842	-1.785144
P	-0.320879	0.323120	2.033016
O	-1.820325	-0.974545	-0.078206
C	-1.138481	-0.717093	-2.329897
C	-1.083277	-1.229446	-3.633154
H	-0.592955	-0.652131	-4.416286
C	-1.606005	-2.492077	-3.917959
H	-1.551511	-2.882286	-4.934681
C	-2.177542	-3.268449	-2.906292
H	-2.550383	-4.264726	-3.140361
C	-2.262011	-2.786065	-1.597151
C	-1.758719	-1.504361	-1.352997
C	-2.880292	-3.541143	-0.420030
C	-2.944967	-5.050381	-0.665070
H	-1.943038	-5.474319	-0.810026
H	-3.557146	-5.272506	-1.549001
H	-3.420536	-5.555365	0.186104
C	-4.319413	-2.998559	-0.203192
H	-4.312558	-1.913128	-0.039029
H	-4.776082	-3.480898	0.672718
H	-4.937629	-3.208093	-1.087777
C	-2.056505	-3.195502	0.821742
C	-1.613936	-1.876139	0.951736
C	-0.947364	-1.390451	2.082432
C	-0.684237	-2.292960	3.120906
H	-0.140476	-1.952160	4.001048
C	-1.076531	-3.625762	3.003485
H	-0.844156	-4.328425	3.803716
C	-1.756560	-4.073249	1.866584
H	-2.047553	-5.120132	1.794810
C	0.794422	1.178231	-3.177154
C	1.785023	0.214462	-3.446778
H	1.823846	-0.696182	-2.846399
C	2.718697	0.435210	-4.454644
H	3.481741	-0.317211	-4.653697
C	2.702510	1.631317	-5.180234
H	3.443881	1.807130	-5.960664
C	1.744849	2.604991	-4.892248
H	1.732615	3.542896	-5.449609
C	0.787710	2.378958	-3.898020
H	0.028311	3.133428	-3.689237
C	-1.675070	2.067460	-1.948689
C	-2.542393	2.099718	-3.052112
H	-2.422772	1.372641	-3.856569
C	-3.560172	3.050585	-3.114712
H	-4.233082	3.069785	-3.973219
C	-3.722993	3.974613	-2.075892
H	-4.523936	4.713612	-2.125748
C	-2.868703	3.941306	-0.972656
H	-2.999558	4.642698	-0.148811
C	-1.850958	2.988054	-0.909103
H	-1.199395	2.941179	-0.037851
C	0.565992	0.523671	3.617098
C	1.910767	0.117795	3.650192
H	2.344493	-0.362495	2.768382
C	2.663858	0.304096	4.809926
H	3.706341	-0.016476	4.829733
C	2.092464	0.908775	5.933771
H	2.689123	1.068085	6.833139
C	0.751720	1.301842	5.905260
H	0.297058	1.763537	6.782976
C	-0.013218	1.103002	4.754487
H	-1.057565	1.413280	4.736831
C	-1.810677	1.368473	2.227351
C	-1.631632	2.735822	2.499526
H	-0.630500	3.131097	2.659081
C	-2.727173	3.591853	2.583749
H	-2.563074	4.647105	2.806359
C	-4.019666	3.102354	2.374465
H	-4.877590	3.773716	2.426854
C	-4.205312	1.746504	2.100117
H	-5.210084	1.353195	1.940695
C	-3.110499	0.881832	2.036085
H	-3.273268	-0.177027	1.843012
C	2.828016	1.565649	-0.147454

4•S (cont)
E = –4869.507212 a.u.
N_{imag} = 0

C	2.124425	2.328001	0.538239
C	3.870154	1.026622	-0.959574
C	1.634135	3.416934	1.322994
C	4.444929	1.837309	-1.959214
C	5.926279	0.028700	-2.577991
C	4.335397	-0.289635	-0.776880
C	5.357414	-0.777996	-1.586844
H	5.711645	-1.799468	-1.443148
H	3.885546	-0.906101	0.001609
C	5.469364	1.337991	-2.757173
H	4.063470	2.846812	-2.109632
H	6.723711	-0.362710	-3.211067
H	5.903412	1.969235	-3.533362
C	1.886005	3.483042	2.708461
C	0.641694	5.558191	2.847095
C	0.889178	4.445671	0.711273
C	0.399528	5.505265	1.470665
H	-0.178512	6.292339	0.985049
H	0.699080	4.395526	-0.360183
C	1.389915	4.546984	3.458633
H	2.456367	2.688131	3.185047
H	0.250721	6.385044	3.440736
H	1.583236	4.578935	4.531102
C	0.601083	-5.205168	-1.670314
C	0.786025	-6.236134	-0.743532
C	1.280966	-5.944996	0.531878
C	1.601155	-4.631064	0.872694
C	1.408740	-3.594617	-0.048803
C	0.899034	-3.888575	-1.319906
H	0.213006	-5.425811	-2.665854
H	0.545071	-7.265413	-1.014838
H	1.421164	-6.747006	1.258489
H	1.996631	-4.374823	1.855311
C	1.727387	-2.176601	0.350286
H	0.735811	-3.071468	-2.021249
O	1.283699	-1.277066	-0.464469
O	2.366863	-1.957597	1.399195

4•A
E = –4884.770455 a.u.
N_{imag} = 0

Cu	1.091183	0.285373	0.424435
P	-0.062497	0.636976	-1.458873
P	-0.096857	0.160543	2.324748
O	-1.751736	-0.961038	0.169498
C	-0.825243	-0.935996	-2.005176
C	-0.644297	-1.561926	-3.242997
H	-0.028282	-1.082285	-4.002393
C	-1.217898	-2.811518	-3.482696
H	-1.051694	-3.304822	-4.440554
C	-1.992125	-3.445411	-2.506039
H	-2.418039	-4.425984	-2.713301
C	-2.215108	-2.836184	-1.267869
C	-1.606620	-1.598820	-1.053177
C	-3.117862	-3.375317	-0.157054
C	-3.372398	-4.877428	-0.300599
H	-2.434626	-5.446052	-0.258609
H	-3.869991	-5.090187	-1.255948
H	-4.042240	-5.234612	0.492878
C	-4.471204	-2.616886	-0.234571
H	-4.323483	-1.533095	-0.141487
H	-5.136677	-2.948825	0.575275
H	-4.958242	-2.817392	-1.199477
C	-2.455337	-3.023485	1.176329
C	-1.794807	-1.792536	1.267184
C	-1.130079	-1.353982	2.419071
C	-1.193546	-2.175343	3.553992
H	-0.685508	-1.868368	4.467435
C	-1.866839	-3.396010	3.504388
H	-1.901956	-4.029469	4.391108
C	-2.479522	-3.822051	2.322031
H	-2.973673	-4.792416	2.294027
C	0.770834	1.257415	-2.962376
C	1.940355	0.598009	-3.379962
H	2.283111	-0.278062	-2.822001
C	2.624011	1.046220	-4.510067
H	3.522918	0.521366	-4.837367
C	2.165139	2.161062	-5.218075
H	2.708272	2.514816	-6.095587
C	1.015698	2.829022	-4.792175
H	0.661681	3.708518	-5.330852
C	0.317663	2.379661	-3.670266
H	-0.579970	2.903197	-3.341855
C	-1.544882	1.707919	-1.319067
C	-2.695569	1.474600	-2.087397
H	-2.713301	0.645936	-2.796322
C	-3.819169	2.287769	-1.935965
H	-4.711973	2.094098	-2.532507
C	-3.802613	3.343670	-1.019550
H	-4.684987	3.973238	-0.895605
C	-2.656852	3.584289	-0.258827
H	-2.639882	4.394747	0.469824
C	-1.533313	2.769113	-0.405130
H	-0.643245	2.946762	0.194583
C	0.982315	-0.064523	3.785708
C	1.966250	-1.066603	3.672683
H	2.010899	-1.664567	2.757444
C	2.868607	-1.279512	4.712959
H	3.622257	-2.063161	4.621595
C	2.817273	-0.485423	5.865106
H	3.531581	-0.646488	6.673464
C	1.847395	0.512441	5.975896
H	1.799452	1.130908	6.873435
C	0.926262	0.718994	4.943896
H	0.162697	1.491072	5.041000
C	-1.229860	1.512628	2.781701
C	-0.701080	2.811353	2.883049
H	0.360861	2.977387	2.698677
C	-1.532726	3.888904	3.180923
H	-1.109266	4.890736	3.265153
C	-2.907576	3.689798	3.346132
H	-3.560759	4.535618	3.564376
C	-3.441972	2.406609	3.217549
H	-4.514845	2.246967	3.332244
C	-2.607274	1.320165	2.947021
H	-3.026525	0.317809	2.858424
C	3.569557	4.828538	-3.567300

4•A (cont)
E = –4884.770455 a.u.
N_{imag} = 0

C	2.608935	5.835931	-3.683043
C	1.579385	5.934766	-2.741706
C	1.519880	5.037358	-1.680122
C	2.487214	4.027764	-1.559658
C	3.509938	3.920199	-2.512639
H	4.361417	4.740656	-4.311319
H	2.656575	6.540482	-4.514411
H	0.821689	6.712890	-2.838426
H	0.724710	5.089029	-0.937310
C	2.360016	3.098392	-0.410978
H	4.241084	3.118718	-2.431408
O	3.324150	2.138048	-0.430951
O	1.501315	3.176961	0.452663
N	3.094533	1.035518	0.546981
C	3.522908	1.539405	1.865740
C	4.026286	0.000057	0.067704
H	2.838862	2.325944	2.193218
H	4.557918	1.915577	1.825334
H	3.459926	0.696405	2.564035
H	5.056996	0.387438	0.033670
H	3.689912	-0.371198	-0.905082
H	3.950221	-0.833436	0.774728
C	0.086895	-5.645214	0.702054
C	0.097113	-6.429100	-0.456180
C	0.626985	-5.910550	-1.642251
C	1.153488	-4.619359	-1.664983
C	1.145602	-3.829870	-0.508431
C	0.601216	-4.349102	0.673182
H	-0.332752	-6.041428	1.627995
H	-0.308967	-7.442019	-0.435136
H	0.629650	-6.517628	-2.549177
H	1.572401	-4.187455	-2.573496
C	1.693492	-2.422574	-0.557783
H	0.582189	-3.716760	1.560124
O	1.556786	-1.742370	0.530470
O	2.211789	-2.011946	-1.619575

4•H
E = –4739.448455 a.u.
N_{imag} = 0

Cu	1.277628	0.372746	-0.028312
P	0.103145	0.602926	-1.919537
P	0.079380	0.075939	1.836116
O	-1.606584	-0.990144	-0.213627
C	-0.901879	-0.830818	-2.459359
C	-0.871093	-1.375622	-3.750648
H	-0.301838	-0.874512	-4.532703
C	-1.536358	-2.572102	-4.023035
H	-1.500044	-2.991103	-5.028860
C	-2.238336	-3.245710	-3.017937
H	-2.737192	-4.185112	-3.253331
C	-2.295305	-2.732336	-1.718585
C	-1.622162	-1.530167	-1.482884
C	-3.056336	-3.363470	-0.549406
C	-3.327969	-4.852573	-0.778781
H	-2.394073	-5.418133	-0.896793
H	-3.942493	-5.001079	-1.676207
H	-3.888491	-5.276827	0.064037
C	-4.408326	-2.614795	-0.397400
H	-4.246248	-1.543101	-0.221679
H	-4.971025	-3.022143	0.454454
H	-5.010376	-2.729845	-1.309916
C	-2.238196	-3.117463	0.720790
C	-1.561407	-1.902224	0.825630
C	-0.811135	-1.517980	1.941341
C	-0.760418	-2.402231	3.024977
H	-0.181423	-2.135181	3.908552
C	-1.430135	-3.625357	2.957503
H	-1.379277	-4.314030	3.801084
C	-2.155036	-3.983108	1.816971
H	-2.657899	-4.949106	1.785474
C	1.101515	0.996369	-3.393737
C	2.453475	0.622370	-3.372790
H	2.868964	0.159311	-2.473197
C	3.262703	0.878150	-4.482179
H	4.314702	0.591975	-4.458531
C	2.732144	1.513742	-5.606661
H	3.368219	1.719638	-6.468609
C	1.386666	1.898054	-5.625395
H	0.973543	2.401876	-6.500333
C	0.571692	1.639872	-4.523682
H	-0.475493	1.945656	-4.531850
C	-1.067214	1.999284	-1.815407
C	-2.442979	1.825849	-1.618477
H	-2.867471	0.823235	-1.590185
C	-3.275086	2.935431	-1.462600
H	-4.343640	2.787886	-1.303175
C	-2.745932	4.226203	-1.508811
H	-3.399878	5.090765	-1.390355
C	-1.372346	4.405448	-1.696997
H	-0.949756	5.410614	-1.729378
C	-0.536181	3.300382	-1.838699
H	0.535751	3.443237	-1.982364
C	0.860399	0.202391	3.482297
C	2.152828	-0.320861	3.642487
H	2.692583	-0.753877	2.798686
C	2.777536	-0.265937	4.889136
H	3.784177	-0.670525	4.997657
C	2.128169	0.323562	5.976595
H	2.624131	0.376934	6.946751
C	0.845657	0.856526	5.817136
H	0.337579	1.324260	6.661673
C	0.210249	0.793651	4.576059
H	-0.789762	1.210240	4.453057
C	-1.285189	1.296923	1.923252
C	-0.986498	2.636417	1.633092
H	0.023162	2.908591	1.328021
C	-1.974940	3.614241	1.715508
H	-1.728292	4.649962	1.483215
C	-3.280929	3.262822	2.066290
H	-4.058000	4.026442	2.116237
C	-3.590391	1.929030	2.338314
H	-4.609294	1.647002	2.607741
C	-2.596202	0.950073	2.274483
H	-2.839954	-0.088453	2.500218
C	0.948809	-3.663108	-1.287871

4•H (cont)

E = –4739.448455 a.u.

N_{imag} = 0

C	0.679664	-4.572799	-0.261812
C	1.302918	-4.427371	0.980179
C	2.196251	-3.378122	1.195417
C	2.450625	-2.444860	0.183357
C	1.818238	-2.598096	-1.059304
H	0.473604	-3.776545	-2.262555
H	-0.013112	-5.398322	-0.430615
H	1.088761	-5.133694	1.782814
H	2.710338	-3.259752	2.149586
C	3.388509	-1.280820	0.446481
H	2.028493	-1.880340	-1.851246
O	3.169985	-0.216709	-0.278280
O	4.260466	-1.381519	1.320722
Si	2.902181	2.934004	0.391413
H	1.633135	2.133662	0.083510
C	4.003096	2.865994	-1.121634
C	3.731566	2.261980	1.924274
C	2.244811	4.681517	0.683478
H	3.467653	3.160497	-2.034812
H	4.363709	1.836408	-1.244211
H	4.870055	3.532821	-1.001562
H	4.089068	1.238164	1.742151
H	3.042241	2.245405	2.779565
H	4.593055	2.891438	2.195577
H	1.593074	4.721371	1.568415
H	1.667567	5.045579	-0.178363
H	3.075730	5.383114	0.852905

4•T

E = –4561.943901 a.u.

N_{imag} = 0

Cu	1.281464	0.478209	0.169180
P	0.103250	0.731793	-1.706529
P	0.101108	0.190764	2.049275
O	-1.605941	-0.842808	-0.060578
C	-0.800746	-0.753442	-2.272781
C	-0.756292	-1.292824	-3.563308
H	-0.165143	-0.796882	-4.332649
C	-1.449280	-2.470961	-3.848864
H	-1.404380	-2.891710	-4.853612
C	-2.188693	-3.125232	-2.859031
H	-2.708050	-4.050811	-3.104895
C	-2.264852	-2.606117	-1.561734
C	-1.568074	-1.423700	-1.315649
C	-3.094073	-3.188300	-0.414020
C	-3.390937	-4.676422	-0.617253
H	-2.466498	-5.267108	-0.663461
H	-3.952620	-4.834339	-1.546900
H	-4.013393	-5.063345	0.200010
C	-4.432456	-2.403980	-0.347057
H	-4.251751	-1.329383	-0.212471
H	-5.040112	-2.762879	0.495876
H	-4.998129	-2.543895	-1.279110
C	-2.328055	-2.928635	0.886438
C	-1.631175	-1.722112	1.001245
C	-0.903037	-1.343022	2.135367
C	-0.892246	-2.231134	3.219887
H	-0.321841	-1.981698	4.113825
C	-1.582345	-3.441879	3.142745
H	-1.561693	-4.128096	3.989659
C	-2.290365	-3.789277	1.987503
H	-2.808909	-4.746564	1.947035
C	0.897278	1.315539	-3.247794
C	2.183788	0.835627	-3.544591
H	2.700303	0.153887	-2.864541
C	2.829766	1.249857	-4.710214
H	3.829621	0.872521	-4.927194
C	2.208570	2.152335	-5.577846
H	2.721049	2.483458	-6.482176
C	0.932785	2.639165	-5.279448
H	0.446407	3.348811	-5.950352
C	0.275925	2.221039	-4.120759
H	-0.717374	2.605356	-3.887508
C	-1.254899	1.932460	-1.441298
C	-2.532515	1.764049	-1.992324
H	-2.748605	0.884533	-2.600094
C	-3.529319	2.713762	-1.759950
H	-4.523622	2.569947	-2.185439
C	-3.252581	3.845285	-0.989459
H	-4.031525	4.586794	-0.807390
C	-1.980176	4.017167	-0.439401
H	-1.764749	4.887007	0.180319
C	-0.988796	3.060012	-0.649948
H	-0.010491	3.172203	-0.180342
C	1.092523	0.092924	3.581254
C	2.413287	-0.369618	3.460911
H	2.813156	-0.603153	2.468945
C	3.211303	-0.498800	4.599832
H	4.236956	-0.856297	4.500804
C	2.703884	-0.158976	5.856379
H	3.331909	-0.253694	6.743284
C	1.392169	0.311426	5.977090
H	0.995971	0.582115	6.956816
C	0.586495	0.434921	4.844706
H	-0.435832	0.803834	4.936887
C	-1.088594	1.531072	2.398999
C	-0.572284	2.783910	2.773080
H	0.505087	2.913626	2.883525
C	-1.429255	3.856237	3.009260
H	-1.017770	4.819577	3.313905
C	-2.809504	3.702185	2.844395
H	-3.479464	4.544761	3.019055
C	-3.324365	2.468433	2.444026
H	-4.398309	2.344287	2.300775
C	-2.470880	1.384735	2.229263
H	-2.884143	0.421059	1.933509
O	2.172673	2.611630	0.436318

4•T (cont)

E = –4561.943901 a.u.

N_{imag} = 0

C	3.220856	2.636304	1.439435
H	2.973896	1.871603	2.183761
H	3.212740	3.630120	1.921960
C	4.543596	2.375912	0.695472
H	5.320300	3.079023	1.025181
H	4.885995	1.350212	0.867594
C	4.180194	2.559608	-0.799901
H	4.889544	3.204095	-1.335433
H	4.154562	1.577912	-1.291322
C	2.784028	3.172161	-0.752737
H	2.818471	4.272243	-0.644151
H	2.146474	2.917075	-1.606370
C	0.839635	-3.904959	0.204614
C	0.580072	-4.471542	-1.046117
C	1.203579	-3.958936	-2.186732
C	2.091570	-2.889414	-2.076391
C	2.340646	-2.300658	-0.830742
C	1.703163	-2.815736	0.306626
H	0.360475	-4.302896	1.099303
H	-0.106204	-5.315208	-1.132968
H	0.994390	-4.394864	-3.164168
H	2.606671	-2.488355	-2.949586
C	3.279530	-1.113893	-0.728593
H	1.902115	-2.358636	1.274913
O	3.098799	-0.348856	0.311499
O	4.124314	-0.923533	-1.618557

TS[**4**•H–**1**•OS$_1$]
E = –4739.419366 a.u.
N_{imag} = 1

Cu	1.147740	0.383653	-0.005221
P	-0.023949	0.657947	-1.873889
P	0.032565	0.110409	1.894083
O	-1.880616	-0.691979	-0.101452
C	-1.229377	-0.629105	-2.372028
C	-1.308086	-1.181181	-3.658640
H	-0.704601	-0.760187	-4.461870
C	-2.125966	-2.285805	-3.901914
H	-2.172995	-2.710668	-4.904844
C	-2.879692	-2.857938	-2.871886
H	-3.504176	-3.724831	-3.084554
C	-2.834657	-2.331249	-1.577359
C	-2.003789	-1.226101	-1.368429
C	-3.642299	-2.845893	-0.383443
C	-4.139048	-4.277920	-0.597731
H	-3.305796	-4.978385	-0.745010
H	-4.798194	-4.331719	-1.473929
H	-4.728774	-4.614641	0.264588
C	-4.864009	-1.907340	-0.188268
H	-4.541269	-0.871275	-0.022439
H	-5.450552	-2.229240	0.683815
H	-5.507440	-1.932385	-1.079072
C	-2.751274	-2.722447	0.854364
C	-1.918688	-1.605133	0.937708
C	-1.082602	-1.333785	2.026380
C	-1.103509	-2.234849	3.098142
H	-0.460028	-2.057482	3.959237
C	-1.924895	-3.362705	3.048722
H	-1.928597	-4.063634	3.883817
C	-2.735839	-3.608506	1.937069
H	-3.361779	-4.500056	1.918078
C	0.959192	0.927655	-3.389020
C	2.274875	0.442376	-3.411451
H	2.680677	-0.054552	-2.527781
C	3.072543	0.631796	-4.542006
H	4.098391	0.262229	-4.546132
C	2.564901	1.311984	-5.650634
H	3.192319	1.470242	-6.528694
C	1.255164	1.803527	-5.630712
H	0.860329	2.342704	-6.492854
C	0.454020	1.613180	-4.505204
H	-0.561889	2.009899	-4.482458
C	-0.992211	2.199659	-1.735941
C	-2.371323	2.216289	-1.496609
H	-2.928021	1.281602	-1.444131
C	-3.038038	3.431085	-1.327730
H	-4.110762	3.433153	-1.132053
C	-2.338367	4.636057	-1.406628
H	-2.863490	5.583165	-1.278200
C	-0.959466	4.623629	-1.636391
H	-0.403240	5.560390	-1.690965
C	-0.285872	3.413615	-1.786812
H	0.792656	3.405934	-1.950894
C	0.870694	0.119334	3.517833
C	2.169015	-0.401168	3.606961
H	2.680193	-0.783410	2.723859
C	2.833236	-0.421698	4.834887
H	3.846069	-0.822412	4.886379
C	2.210642	0.083985	5.977696
H	2.735178	0.078445	6.934137
C	0.917000	0.608743	5.892493
H	0.429824	1.010384	6.782054
C	0.247744	0.626739	4.668605
H	-0.757456	1.044716	4.601696
C	-1.111730	1.533964	2.006232
C	-0.582660	2.799831	1.709965
H	0.462662	2.884375	1.406728
C	-1.390125	3.931814	1.782965
H	-0.971368	4.909366	1.545211
C	-2.738623	3.809703	2.129233
H	-3.374129	4.695135	2.166963
C	-3.274103	2.551331	2.408652
H	-4.328224	2.450854	2.671232
C	-2.462524	1.415716	2.353980
H	-2.881230	0.433915	2.578243
C	0.760355	-4.296031	1.045473

TS[**4**•H–**1**•OS$_1$] (cont)
E = –4739.419366 a.u.
N_{imag} = 1

C	0.057976	-4.339825	-0.160809
C	0.415335	-3.486839	-1.208973
C	1.452943	-2.572981	-1.036714
C	2.157317	-2.516675	0.176235
C	1.813266	-3.396008	1.209088
H	0.479189	-4.957674	1.864572
H	-0.769014	-5.039496	-0.284887
H	-0.123310	-3.524198	-2.156209
H	1.731006	-1.903406	-1.848760
C	3.236718	-1.489625	0.420862
H	2.376945	-3.346847	2.140791
O	4.108965	-1.661955	1.272163
O	3.077125	-0.393304	-0.302065
Si	3.818814	1.622619	-0.002636
H	2.195015	1.696055	0.069258
C	3.736660	3.065983	-1.248296
C	5.481522	0.823266	-0.446775
C	4.027496	2.225307	1.775938
H	5.022441	2.673426	1.919507
H	3.931884	1.392749	2.486269
H	3.260988	2.971014	2.031145
H	6.269265	1.592573	-0.386627
H	5.487773	0.411073	-1.466051
H	5.716910	0.009887	0.252093
H	3.015425	3.826331	-0.913202
H	3.393370	2.692782	-2.225974
H	4.717336	3.540950	-1.402143

1•OS$_1$
E = –4739.438881 a.u.
N_{imag} = 0

Cu	1.442442	0.690701	0.096101
P	0.182963	0.987488	-1.742544
P	0.251866	0.391090	1.986612
O	-1.845501	0.043079	0.060437
C	-1.330966	0.065911	-2.239778
C	-1.652453	-0.331500	-3.545211
H	-1.023564	-0.011950	-4.375293
C	-2.752785	-1.157495	-3.779964
H	-2.991953	-1.459201	-4.800306
C	-3.540278	-1.617995	-2.720604
H	-4.381911	-2.278449	-2.926736
C	-3.256528	-1.238966	-1.404421
C	-2.166772	-0.386836	-1.211120
C	-4.071665	-1.638707	-0.172858
C	-4.880866	-2.916741	-0.409897
H	-4.228658	-3.766653	-0.650266
H	-5.591668	-2.778078	-1.234791
H	-5.471964	-3.170486	0.479515
C	-5.043334	-0.473372	0.158443
H	-4.491160	0.458197	0.339433
H	-5.624280	-0.711141	1.060867
H	-5.736547	-0.308872	-0.678736
C	-3.097826	-1.781759	0.999470
C	-2.037075	-0.877441	1.072275
C	-1.117178	-0.826382	2.127413
C	-1.281542	-1.753260	3.164891
H	-0.589798	-1.745230	4.006295
C	-2.316226	-2.690428	3.113439
H	-2.427978	-3.412705	3.922609
C	-3.217642	-2.704313	2.045000
H	-4.022813	-3.438337	2.033515
C	1.108760	1.077133	-3.317503
C	2.273053	0.307312	-3.432853
H	2.609729	-0.272757	-2.573342
C	3.010879	0.316428	-4.617810
H	3.919474	-0.282047	-4.696415
C	2.596288	1.106658	-5.692294
H	3.176407	1.121418	-6.615907
C	1.445631	1.893552	-5.576845
H	1.128178	2.523101	-6.409340
C	0.704569	1.880661	-4.394769
H	-0.184829	2.504973	-4.298676
C	-0.429379	2.704456	-1.561625
C	-1.778675	3.018336	-1.360666
H	-2.525707	2.225915	-1.333943
C	-2.174590	4.347853	-1.203037
H	-3.227231	4.579969	-1.037625
C	-1.230691	5.374362	-1.258931
H	-1.543911	6.412781	-1.143563
C	0.119565	5.065051	-1.448632
H	0.864871	5.861113	-1.481116
C	0.522850	3.738247	-1.584121
H	1.578554	3.491241	-1.702292
C	1.048248	0.247398	3.631466
C	1.859275	-0.869338	3.890115
H	2.006009	-1.627555	3.119746
C	2.484150	-1.012335	5.128984
H	3.107785	-1.887420	5.317578
C	2.325127	-0.035780	6.115870
H	2.823296	-0.144069	7.080250
C	1.529360	1.082633	5.858941
H	1.401529	1.851241	6.622593
C	0.888471	1.222960	4.626173
H	0.264239	2.095594	4.436460
C	-0.637917	1.990553	2.114408
C	0.073513	3.157594	1.793853
H	1.102777	3.071309	1.438876
C	-0.538949	4.404557	1.911179
H	0.020203	5.304013	1.655050
C	-1.868473	4.499028	2.328351
H	-2.349655	5.474992	2.404177
C	-2.583814	3.339749	2.636103
H	-3.623541	3.406794	2.960034
C	-1.969203	2.090175	2.538161
H	-2.526516	1.188884	2.794644
C	-1.158614	-4.156057	-0.350730

1•OS$_1$ (cont)
E = –4739.438881 a.u.
N_{imag} = 0

C	-1.299882	-3.965923	-1.728323
C	-0.353879	-3.217243	-2.432480
C	0.728856	-2.654804	-1.762066
C	0.884449	-2.859569	-0.383593
C	-0.066182	-3.613511	0.319586
H	-1.899335	-4.731372	0.204165
H	-2.150390	-4.397638	-2.256612
H	-0.467615	-3.061441	-3.504038
H	1.469602	-2.071772	-2.304495
C	2.090639	-2.392389	0.344720
H	0.070021	-3.763217	1.389368
O	2.417402	-2.822667	1.444541
O	2.828525	-1.478325	-0.329032
Si	4.478105	-1.144070	0.129612
H	2.676101	1.657570	0.083813
C	4.600267	-0.329387	1.799578
C	5.073156	-0.020849	-1.237783
C	5.374495	-2.790267	0.075614
H	6.452718	-2.633876	0.231332
H	5.246042	-3.282418	-0.899449
H	5.009709	-3.468287	0.857752
H	6.113966	0.278470	-1.041855
H	4.451714	0.884736	-1.269993
H	5.046199	-0.514712	-2.219101
H	3.911998	0.525800	1.844480
H	5.625540	0.043195	1.949326
H	4.360344	-1.026878	2.610165

TS[**4**•H–**1**•OS$_2$]
E = –4739.418912 a.u.
N_{imag} = 1

Cu	1.147740	0.383653	-0.005221
P	-0.023949	0.657947	-1.873889
P	0.032565	0.110409	1.894083
O	-1.880616	-0.691979	-0.101452
C	-1.229377	-0.629105	-2.372028
C	-1.308086	-1.181181	-3.658640
H	-0.704601	-0.760187	-4.461870
C	-2.125966	-2.285805	-3.901914
H	-2.172995	-2.710668	-4.904844
C	-2.879692	-2.857938	-2.871886
H	-3.504176	-3.724831	-3.084554
C	-2.834657	-2.331249	-1.577359
C	-2.003789	-1.226101	-1.368429
C	-3.642299	-2.845893	-0.383443
C	-4.139048	-4.277920	-0.597731
H	-3.305796	-4.978385	-0.745010
H	-4.798194	-4.331719	-1.473929
H	-4.728774	-4.614641	0.264588
C	-4.864009	-1.907340	-0.188268
H	-4.541269	-0.871275	-0.022439
H	-5.450552	-2.229240	0.683815
H	-5.507440	-1.932385	-1.079072
C	-2.751274	-2.722447	0.854364
C	-1.918688	-1.605133	0.937708
C	-1.082602	-1.333785	2.026380
C	-1.103509	-2.234849	3.098142
H	-0.460028	-2.057482	3.959237
C	-1.924895	-3.362705	3.048722
H	-1.928597	-4.063634	3.883817
C	-2.735839	-3.608506	1.937069
H	-3.361779	-4.500056	1.918078
C	0.959192	0.927655	-3.389020
C	2.274875	0.442376	-3.411451
H	2.680677	-0.054552	-2.527781
C	3.072543	0.631796	-4.542006
H	4.098391	0.262229	-4.546132
C	2.564901	1.311984	-5.650634
H	3.192319	1.470242	-6.528694
C	1.255164	1.803527	-5.630712
H	0.860329	2.342704	-6.492854
C	0.454020	1.613180	-4.505204
H	-0.561889	2.009899	-4.482458
C	-0.992211	2.199659	-1.735941
C	-2.371323	2.216289	-1.496609
H	-2.928021	1.281602	-1.444131
C	-3.038038	3.431085	-1.327730
H	-4.110762	3.433153	-1.132053
C	-2.338367	4.636057	-1.406628
H	-2.863490	5.583165	-1.278200
C	-0.959466	4.623629	-1.636391
H	-0.403240	5.560390	-1.690965
C	-0.285872	3.413615	-1.786812
H	0.792656	3.405934	-1.950894
C	0.870694	0.119334	3.517833
C	2.169015	-0.401168	3.606961
H	2.680193	-0.783410	2.723859
C	2.833236	-0.421698	4.834887
H	3.846069	-0.822412	4.886379
C	2.210642	0.083985	5.977696
H	2.735178	0.078445	6.934137
C	0.917000	0.608743	5.892493
H	0.429824	1.010384	6.782054
C	0.247744	0.626739	4.668605
H	-0.757456	1.044716	4.601696
C	-1.111730	1.533964	2.006232
C	-0.582660	2.799831	1.709965
H	0.462662	2.884375	1.406728
C	-1.390125	3.931814	1.782965
H	-0.971368	4.909366	1.545211
C	-2.738623	3.809703	2.129233
H	-3.374129	4.695135	2.166963
C	-3.274103	2.551331	2.408652
H	-4.328224	2.450854	2.671232
C	-2.462524	1.415716	2.353980
H	-2.881230	0.433915	2.578243
C	0.760355	-4.296031	1.045473

TS[**4**•H–**1**•OS$_2$] (cont)
E = –4739.418912 a.u.
N_{imag} = 1

C	0.057976	-4.339825	-0.160809
C	0.415335	-3.486839	-1.208973
C	1.452943	-2.572981	-1.036714
C	2.157317	-2.516675	0.176235
C	1.813266	-3.396008	1.209088
H	0.479189	-4.957674	1.864572
H	-0.769014	-5.039496	-0.284887
H	-0.123310	-3.524198	-2.156209
H	1.731006	-1.903406	-1.848760
C	3.236718	-1.489625	0.420862
H	2.376945	-3.346847	2.140791
O	4.108965	-1.661955	1.272163
O	3.077125	-0.393304	-0.302065
Si	3.818814	1.622619	-0.002636
H	2.195015	1.696055	0.069258
C	3.736660	3.065983	-1.248296
C	5.481522	0.823266	-0.446775
C	4.027496	2.225307	1.775938
H	5.022441	2.673426	1.919507
H	3.931884	1.392749	2.486269
H	3.260988	2.971014	2.031145
H	6.269265	1.592573	-0.386627
H	5.487773	0.411073	-1.466051
H	5.716910	0.009887	0.252093
H	3.015425	3.826331	-0.913202
H	3.393370	2.692782	-2.225974
H	4.717336	3.540950	-1.402143

1•OS_2
E = 4739.435154 a.u.
N_{imag} = 0

Cu	1.438398	0.628542	-0.245520
P	0.179194	1.010413	-2.076428
P	0.210502	0.443212	1.642766
O	-1.913487	0.252354	-0.256984
C	-1.373633	0.141956	-2.549498
C	-1.675376	-0.342755	-3.830531
H	-1.003967	-0.122555	-4.659684
C	-2.816109	-1.120769	-4.040790
H	-3.037217	-1.491898	-5.042088
C	-3.678070	-1.429037	-2.983216
H	-4.560185	-2.039920	-3.171866
C	-3.409343	-0.968645	-1.689856
C	-2.258132	-0.195710	-1.517217
C	-4.286302	-1.214153	-0.461239
C	-5.222886	-2.410052	-0.651728
H	-4.662213	-3.337130	-0.832176
H	-5.898563	-2.241157	-1.500242
H	-5.854285	-2.552095	0.234730
C	-5.135073	0.062351	-0.212414
H	-4.491072	0.938132	-0.059349
H	-5.757943	-0.067737	0.683910
H	-5.787810	0.257132	-1.075213
C	-3.352885	-1.396224	0.737817
C	-2.200282	-0.608779	0.784046
C	-1.287949	-0.609991	1.847764
C	-1.590707	-1.432239	2.941862
H	-0.930311	-1.441614	3.807296
C	-2.728778	-2.240436	2.926576
H	-2.944741	-2.881114	3.782226
C	-3.597056	-2.233027	1.831807
H	-4.478243	-2.873599	1.841416
C	1.101346	1.011124	-3.654729
C	2.199568	0.146612	-3.767415
H	2.496428	-0.453831	-2.905721
C	2.918022	0.081889	-4.963627
H	3.773138	-0.590431	-5.045017
C	2.553760	0.886796	-6.045510
H	3.121061	0.841192	-6.976207
C	1.468742	1.762150	-5.931318
H	1.189067	2.399520	-6.771451
C	0.743874	1.824556	-4.741011
H	-0.097344	2.512603	-4.646474
C	-0.354967	2.754873	-1.924012
C	-1.684865	3.139889	-1.718459
H	-2.473150	2.388745	-1.687501
C	-2.010385	4.488474	-1.559299
H	-3.048642	4.775237	-1.388110
C	-1.015018	5.464426	-1.622874
H	-1.272748	6.517819	-1.505853
C	0.315604	5.084739	-1.824497
H	1.101162	5.840776	-1.865237
C	0.648679	3.738573	-1.957594
H	1.689941	3.436710	-2.077302
C	1.097280	0.171202	3.221944
C	1.509823	-1.125874	3.570503
H	1.258279	-1.964966	2.924805
C	2.251717	-1.348157	4.729288
H	2.545698	-2.364555	4.994574
C	2.627128	-0.273942	5.540980
H	3.219795	-0.446059	6.440011
C	2.241692	1.021239	5.188507
H	2.534101	1.867348	5.811799
C	1.474186	1.243342	4.043133
H	1.169724	2.256498	3.781830
C	-0.509514	2.121228	1.849154
C	0.241584	3.225281	1.418297
H	1.213742	3.050424	0.950282
C	-0.265331	4.516296	1.566533
H	0.323190	5.366287	1.222269
C	-1.529325	4.716999	2.124544
H	-1.930287	5.726778	2.222903
C	-2.284004	3.619717	2.546543
H	-3.273485	3.769360	2.981364
C	-1.773784	2.327148	2.417953
H	-2.363548	1.474683	2.755825
C	-0.863412	-4.216085	0.678386

1•OS_2 (cont)
E = 4739.435154 a.u.
N_{imag} = 0

C	-1.550562	-4.038132	-0.523861
C	-0.994983	-3.256675	-1.542635
C	0.233775	-2.637254	-1.348009
C	0.928136	-2.807193	-0.140310
C	0.379201	-3.613666	0.867115
H	-1.299549	-4.820589	1.473236
H	-2.522847	-4.510170	-0.668599
H	-1.527051	-3.117028	-2.482835
H	0.675334	-2.015263	-2.124440
C	2.235278	-2.127427	0.020004
H	0.932530	-3.763690	1.792634
O	2.651566	-1.272705	-0.774107
O	2.951167	-2.521574	1.079929
Si	4.536710	-1.874830	1.393274
H	2.609523	1.672900	-0.165241
C	4.436938	-0.084899	1.885615
C	5.098074	-2.975638	2.800821
C	5.605404	-2.152104	-0.118656
H	6.662682	-1.981079	0.134796
H	5.512080	-3.182712	-0.490559
H	5.323953	-1.464180	-0.925172
H	6.121217	-2.709194	3.104974
H	4.447835	-2.855054	3.677569
H	5.095931	-4.035596	2.509660
H	5.454080	0.335369	1.927591
H	3.853197	0.500424	1.154358
H	3.976381	0.037872	2.874459

2•R
E = –4604.461627 a.u.
N_{imag} = 0

Cu	0.474912	0.738663	0.192919
P	-0.725560	0.966789	-1.661307
P	-0.740646	0.468330	2.042345
O	-2.017540	-1.032918	-0.080237
C	-1.150036	-0.712288	-2.249609
C	-0.843386	-1.228423	-3.514219
H	-0.391259	-0.579684	-4.264129
C	-1.085283	-2.574607	-3.794236
H	-0.833265	-2.974910	-4.776549
C	-1.633350	-3.419165	-2.824304
H	-1.793812	-4.470134	-3.061741
C	-1.971782	-2.932789	-1.556743
C	-1.722513	-1.582089	-1.314133
C	-2.640852	-3.743939	-0.444113
C	-2.406855	-5.247411	-0.613368
H	-2.918635	-5.810955	0.177555
H	-1.335722	-5.485285	-0.581492
H	-2.818671	-5.597241	-1.568953
C	-4.165057	-3.451478	-0.494011
H	-4.363416	-2.377537	-0.380750
H	-4.679397	-3.986707	0.316829
H	-4.580877	-3.779645	-1.457280
C	-2.104530	-3.229516	0.894612
C	-1.834377	-1.864132	1.008561
C	-1.336882	-1.259539	2.169982
C	-1.154192	-2.075415	3.294744
H	-0.755082	-1.644778	4.212497
C	-1.439474	-3.439114	3.224178
H	-1.285672	-4.068137	4.101366
C	-1.894886	-4.012977	2.033487
H	-2.080047	-5.085711	1.994969
C	0.134572	1.695897	-3.096275
C	1.504834	1.410978	-3.230755
H	1.998120	0.790218	-2.478324
C	2.228083	1.939945	-4.299225
H	3.290069	1.711110	-4.396987
C	1.596942	2.771795	-5.229943
H	2.165406	3.193205	-6.060083
C	0.238456	3.068367	-5.091189
H	-0.255184	3.720392	-5.813274
C	-0.494186	2.529422	-4.030717
H	-1.555079	2.758370	-3.923902
C	-2.334146	1.826426	-1.609000
C	-3.493809	1.323271	-2.213274
H	-3.451076	0.382453	-2.763533
C	-4.699402	2.019893	-2.105329
H	-5.600416	1.618311	-2.571518
C	-4.751208	3.228399	-1.407167
H	-5.693753	3.770525	-1.322080
C	-3.596910	3.733607	-0.802125
H	-3.637220	4.664037	-0.236607
C	-2.398288	3.029238	-0.888979
H	-1.507254	3.394872	-0.376063
C	0.167207	0.704001	3.611189
C	1.563036	0.566209	3.576565
H	2.052263	0.356157	2.620853
C	2.311769	0.698472	4.747367
H	3.396248	0.587871	4.710528
C	1.672994	0.978533	5.957924
H	2.257890	1.087714	6.872175
C	0.282565	1.125249	5.996415
H	-0.217632	1.346965	6.940248
C	-0.469339	0.987209	4.829068
H	-1.553283	1.104520	4.857913
C	-2.219884	1.513938	2.250442
C	-2.025238	2.873777	2.552137
H	-1.012640	3.253136	2.700378
C	-3.117478	3.731633	2.661922
H	-2.956672	4.781021	2.913819
C	-4.412929	3.253960	2.438167
H	-5.266445	3.928584	2.513860
C	-4.607349	1.912593	2.104577
H	-5.613394	1.536682	1.914714
C	-3.518711	1.042861	2.018551
H	-3.680094	-0.007061	1.775385
H	3.497294	1.508618	-0.077064

2•R (cont)
E = –4604.461627 a.u.
N_{imag} = 0

C	3.436335	0.409116	-0.081863
C	2.206698	-0.154603	0.027863
C	4.764219	-0.195099	-0.256039
C	1.942408	-1.597771	-0.069915
C	5.040949	-1.573938	-0.131124
C	7.387323	-1.203834	-0.638492
C	5.846139	0.657009	-0.564438
C	7.135870	0.165752	-0.756579
H	7.948582	0.853261	-0.997908
H	5.655480	1.729340	-0.657650
C	6.331105	-2.064553	-0.321397
H	4.238423	-2.263121	0.120612
H	8.394397	-1.595735	-0.786530
H	6.515226	-3.135378	-0.217593
C	1.694847	-2.387348	1.073594
C	1.298120	-4.349600	-0.292804
C	1.829068	-2.224491	-1.329797
C	1.513538	-3.576718	-1.437888
H	1.435950	-4.030835	-2.426650
H	1.998698	-1.632655	-2.229893
C	1.388814	-3.741903	0.963185
H	1.755984	-1.926050	2.059751
H	1.066908	-5.412094	-0.377545
H	1.212407	-4.325621	1.867376
C	1.706732	3.671898	-0.334409
O	1.277947	2.972952	0.854124
H	0.806231	4.180082	-0.706915
C	2.818829	4.665301	-0.046328
H	2.013859	2.946551	-1.104699
H	2.028053	2.427507	1.163407
H	3.719742	4.148456	0.317753
H	2.507900	5.394950	0.713987
H	3.092633	5.208544	-0.962853

TS[**2**•R–**5**•O]
E = –4604.441178 a.u.
N_{imag} = 1

Cu	0.793061	1.018415	0.293906
P	-0.440009	1.318371	-1.529817
P	-0.369581	0.779317	2.213266
O	-1.741479	-0.626270	0.112245
C	-1.066834	-0.300271	-2.113042
C	-0.929838	-0.784930	-3.417756
H	-0.525314	-0.134406	-4.192220
C	-1.257182	-2.111512	-3.703924
H	-1.135033	-2.491359	-4.718447
C	-1.720131	-2.965114	-2.698962
H	-1.950500	-4.000450	-2.947091
C	-1.905794	-2.506927	-1.387282
C	-1.580778	-1.172392	-1.143089
C	-2.520234	-3.342433	-0.259425
C	-2.179466	-4.830774	-0.422883
H	-1.094546	-4.993989	-0.376214
H	-2.555662	-5.214001	-1.379817
H	-2.657776	-5.428298	0.363431
C	-4.058197	-3.146901	-0.310461
H	-4.320862	-2.086442	-0.197318
H	-4.540278	-3.710756	0.500781
H	-4.454369	-3.499620	-1.273306
C	-2.016792	-2.812514	1.086660
C	-1.632008	-1.474247	1.191991
C	-1.109460	-0.898453	2.360000
C	-1.059573	-1.701266	3.506660
H	-0.643459	-1.299687	4.429427
C	-1.507548	-3.023070	3.457687
H	-1.469511	-3.638640	4.356586
C	-1.961231	-3.576664	2.258198
H	-2.264020	-4.622685	2.236231
C	0.195578	2.072753	-3.070341
C	1.526346	1.814545	-3.431905
H	2.153524	1.194593	-2.792476
C	2.045375	2.334735	-4.617299
H	3.078829	2.114315	-4.887366
C	1.246674	3.130276	-5.443226
H	1.655034	3.543702	-6.366487
C	-0.077198	3.400043	-5.081940
H	-0.704079	4.023220	-5.722094
C	-0.604332	2.871591	-3.902367
H	-1.637978	3.081511	-3.625485
C	-1.961699	2.279646	-1.218435
C	-3.229491	1.857946	-1.636901
H	-3.339248	0.898741	-2.144259
C	-4.346611	2.666009	-1.414346
H	-5.331987	2.329029	-1.739505
C	-4.201426	3.903676	-0.785548
H	-5.073681	4.535057	-0.612843
C	-2.938660	4.325246	-0.359787
H	-2.824323	5.280668	0.151529
C	-1.826299	3.511940	-0.562033
H	-0.842791	3.826340	-0.207943
C	0.529660	0.979018	3.791320
C	1.930753	1.014443	3.758723
H	2.451202	0.997040	2.800565
C	2.652870	1.133609	4.949300
H	3.742345	1.165988	4.915503
C	1.984642	1.225185	6.171202
H	2.551189	1.320795	7.098505
C	0.585578	1.211419	6.206421
H	0.060284	1.297485	7.158729
C	-0.139667	1.092320	5.022241
H	-1.230380	1.098386	5.047081
C	-1.786426	1.912285	2.451198
C	-1.519355	3.217614	2.897365
H	-0.491985	3.514956	3.113507
C	-2.561000	4.123349	3.088617
H	-2.342448	5.128614	3.451707
C	-3.878637	3.746124	2.814829
H	-4.693613	4.455754	2.961338
C	-4.145130	2.460544	2.340763
H	-5.168727	2.164066	2.109570
C	-3.106786	1.545446	2.161721
H	-3.327545	0.540469	1.804557
H	1.838944	-1.165485	1.912635

TS[**2**•R–**5**•O] (cont)
E = –4604.441178 a.u.
N_{imag} = 1

C	2.188455	-1.446472	0.911886
C	2.448922	-0.447174	0.025408
C	2.255160	-2.904793	0.727023
C	2.863494	-0.714447	-1.361064
C	3.118070	-3.552426	-0.179721
C	2.185485	-5.712606	0.412735
C	1.395945	-3.705038	1.502426
C	1.347811	-5.088204	1.340720
H	0.658771	-5.679982	1.946044
H	0.747852	-3.220129	2.231715
C	3.080185	-4.936658	-0.332952
H	3.824263	-2.964814	-0.762520
H	2.156420	-6.795323	0.283282
H	3.758370	-5.416993	-1.040017
C	4.054344	-0.155896	-1.868558
C	3.616392	-1.072210	-4.069052
C	2.061975	-1.459690	-2.250369
C	2.428825	-1.628818	-3.582576
H	1.780196	-2.200770	-4.247677
H	1.141493	-1.907166	-1.877810
C	4.430790	-0.339981	-3.199892
H	4.685868	0.430080	-1.198864
H	3.900388	-1.202992	-5.113726
H	5.361333	0.100197	-3.562733
C	3.129672	2.991357	-0.166529
O	2.625166	2.078752	0.796840
H	2.510845	3.907749	-0.142322
C	4.586948	3.345183	0.108467
H	3.030990	2.577176	-1.188711
H	2.726614	0.930432	0.460422
H	5.218114	2.445573	0.058934
H	4.693173	3.776451	1.114100
H	4.964748	4.072598	-0.627263

5•O

E = –4604.468162 a.u.

N_{imag} = 0

Cu	1.226490	0.634165	0.299784
P	-0.160700	0.865688	-1.536640
P	-0.090986	0.343135	2.139354
O	-1.760796	-0.779794	0.068247
C	-0.952927	-0.666054	-2.143635
C	-0.799913	-1.278773	-3.396173
H	-0.234100	-0.774555	-4.178195
C	-1.348891	-2.540509	-3.636253
H	-1.209813	-3.009147	-4.610886
C	-2.080287	-3.205715	-2.648313
H	-2.506317	-4.184565	-2.864163
C	-2.266781	-2.624287	-1.389152
C	-1.673675	-1.380516	-1.175266
C	-3.125322	-3.199562	-0.259959
C	-3.357490	-4.703865	-0.420622
H	-2.408626	-5.255186	-0.404761
H	-3.872474	-4.914512	-1.366927
H	-4.001654	-5.084334	0.382733
C	-4.493486	-2.464475	-0.286336
H	-4.359992	-1.380240	-0.174941
H	-5.129985	-2.821752	0.535628
H	-5.004733	-2.653257	-1.240888
C	-2.431740	-2.862065	1.061331
C	-1.774389	-1.632823	1.153522
C	-1.084633	-1.194437	2.291420
C	-1.101511	-2.036891	3.412295
H	-0.564195	-1.745936	4.313997
C	-1.774730	-3.258538	3.366082
H	-1.779844	-3.903394	4.245364
C	-2.425339	-3.672734	2.200084
H	-2.921853	-4.642123	2.179337
C	0.524360	1.746909	-2.983900
C	1.703764	2.475636	-2.764339
H	2.150301	2.480205	-1.763860
C	2.270223	3.204039	-3.810324
H	3.190719	3.762966	-3.637990
C	1.673977	3.207041	-5.073531
H	2.130032	3.764174	-5.893308
C	0.479786	2.510468	-5.282757
H	-0.005412	2.535444	-6.259768
C	-0.103914	1.795402	-4.236337
H	-1.057070	1.289016	-4.392657
C	-1.574749	1.971378	-1.178705
C	-2.895055	1.698573	-1.552205
H	-3.143566	0.750478	-2.031358
C	-3.895172	2.645589	-1.321594
H	-4.924335	2.427467	-1.611566
C	-3.576254	3.870385	-0.732239
H	-4.356924	4.611607	-0.556068
C	-2.257622	4.139160	-0.354102
H	-2.009547	5.086560	0.124553
C	-1.256934	3.192104	-0.562599
H	-0.225004	3.372128	-0.241869
C	0.806130	0.412693	3.735811
C	2.202846	0.316814	3.726374
H	2.721913	0.225737	2.771980
C	2.928825	0.382368	4.917354
H	4.017045	0.316655	4.893124
C	2.260234	0.552437	6.130702
H	2.824467	0.611050	7.062283
C	0.865758	0.666794	6.149274
H	0.342097	0.816413	7.094422
C	0.142361	0.601548	4.959361
H	-0.942380	0.715552	4.971851
C	-1.323687	1.670363	2.393440
C	-0.845914	2.935267	2.774183
H	0.226718	3.099408	2.881171
C	-1.737374	3.976175	3.022637
H	-1.355198	4.950641	3.329685
C	-3.111716	3.777274	2.866077
H	-3.808726	4.595231	3.051789
C	-3.587405	2.532195	2.452164
H	-4.656606	2.374846	2.307168
C	-2.700098	1.479805	2.222271
H	-3.084986	0.507965	1.916941
H	2.523168	-1.337286	1.432436

5•O (cont)

E = –4604.468162 a.u.

N_{imag} = 0

C	2.352703	-1.398063	0.354503
C	3.078921	-0.446961	-0.351500
C	1.639829	-2.635888	0.024574
C	3.385457	-0.227530	-1.768916
C	1.520467	-3.225631	-1.249746
C	0.377312	-5.151075	-0.318571
C	1.122008	-3.361973	1.120937
C	0.504589	-4.596580	0.957327
H	0.122234	-5.125494	1.829721
H	1.223070	-2.941564	2.121821
C	0.892387	-4.457494	-1.415604
H	1.955965	-2.744186	-2.117678
H	-0.099601	-6.122499	-0.454633
H	0.815963	-4.884010	-2.416236
C	4.574176	0.475168	-2.057773
C	4.154363	0.334396	-4.435409
C	2.578193	-0.603106	-2.857422
C	2.959104	-0.335119	-4.168646
H	2.297645	-0.618763	-4.988066
H	1.609146	-1.057080	-2.676016
C	4.957374	0.746648	-3.368558
H	5.197485	0.813808	-1.228511
H	4.443401	0.557525	-5.462509
H	5.882352	1.292677	-3.558693
C	2.596804	3.047186	1.423664
O	1.873259	2.480286	0.380048
H	2.181950	2.792729	2.429096
C	4.083688	2.670929	1.437210
H	2.529357	4.156035	1.349556
H	3.724084	0.159056	0.288168
H	4.534772	2.881189	0.456172
H	4.219750	1.599444	1.652256
H	4.633405	3.236320	2.207388

5•H

E = –4473.184047 a.u.

N_{imag} = 0

Cu	1.093987	0.318614	0.022637
P	-0.125961	0.465395	-1.816329
P	-0.081192	-0.019865	1.879342
O	-1.512837	-1.458440	-0.218092
C	-0.651895	-1.180117	-2.402058
C	-0.366763	-1.719546	-3.662940
H	0.111364	-1.094253	-4.416985
C	-0.658925	-3.058173	-3.931688
H	-0.423713	-3.475802	-4.910952
C	-1.239171	-3.873580	-2.954512
H	-1.445239	-4.918294	-3.184828
C	-1.557461	-3.362431	-1.690241
C	-1.255646	-2.019968	-1.456838
C	-2.242796	-4.138022	-0.562197
C	-2.101453	-5.652429	-0.738040
H	-1.047650	-5.962398	-0.737380
H	-2.560110	-5.975099	-1.681802
H	-2.623145	-6.185541	0.067417
C	-3.749167	-3.762027	-0.566922
H	-3.885298	-2.679209	-0.448506
H	-4.267659	-4.269212	0.259065
H	-4.210855	-4.066269	-1.517013
C	-1.634193	-3.653478	0.757214
C	-1.315133	-2.296769	0.865616
C	-0.748008	-1.718704	2.009792
C	-0.542230	-2.551574	3.119490
H	-0.088409	-2.139986	4.020639
C	-0.868872	-3.905640	3.051933
H	-0.695083	-4.546341	3.916791
C	-1.395143	-4.455249	1.878378
H	-1.619344	-5.520714	1.841454
C	0.820034	1.157809	-3.208070
C	2.188308	0.832923	-3.241301
H	2.604475	0.192268	-2.455351
C	2.999925	1.357703	-4.246637
H	4.060562	1.103875	-4.271940
C	2.461916	2.223833	-5.204743
H	3.102766	2.645326	-5.980364
C	1.105641	2.558683	-5.162502
H	0.687413	3.239983	-5.904870
C	0.281805	2.022831	-4.169821
H	-0.776597	2.284022	-4.131366
C	-1.671891	1.429437	-1.748318
C	-2.878652	0.981061	-2.301078
H	-2.908728	0.027660	-2.830140
C	-4.039307	1.746652	-2.167768
H	-4.976885	1.386768	-2.594180
C	-3.999640	2.969489	-1.494901
H	-4.907281	3.564895	-1.388746
C	-2.797191	3.422190	-0.943782
H	-2.764691	4.366082	-0.400060
C	-1.643033	2.651575	-1.057666
H	-0.713421	2.982331	-0.593508
C	0.961503	0.151501	3.363118
C	2.331674	-0.104388	3.179264
H	2.700193	-0.356388	2.175143
C	3.205119	0.000004	4.263375
H	4.268329	-0.196860	4.119089
C	2.724403	0.371613	5.522283
H	3.411877	0.463099	6.364434
C	1.362502	0.633861	5.702950
H	0.987372	0.927383	6.684452
C	0.479983	0.520047	4.627517
H	-0.582430	0.726368	4.764709
C	-1.490890	1.099191	2.147279
C	-1.222097	2.435664	2.493774
H	-0.194019	2.745359	2.687787
C	-2.259429	3.359301	2.600431
H	-2.038395	4.389111	2.885307
C	-3.576494	2.971120	2.334892
H	-4.387208	3.696854	2.406864
C	-3.846465	1.651675	1.967178
H	-4.869347	1.344244	1.746811
C	-2.813164	0.717827	1.879612
H	-3.035309	-0.311652	1.600487
C	2.567069	-2.057422	-0.026398

5•H (cont)
E = –4473.184047 a.u.
N_{imag} = 0

O	2.744671	-0.667641	-0.060565
H	1.898052	-2.411813	-0.845765
C	3.910022	-2.776395	-0.146206
H	2.070276	-2.389983	0.918235
Si	2.510430	2.958139	0.271459
H	1.286456	2.033902	0.278213
C	4.052659	2.107532	-0.355331
C	2.685960	3.520598	2.058780
C	1.981237	4.363563	-0.865241
H	4.053442	1.044430	-0.063409
H	4.952475	2.604614	0.037003
H	4.087394	2.149743	-1.452782
H	2.855632	2.657774	2.718637
H	1.781430	4.040579	2.405867
H	3.535040	4.210885	2.174206
H	1.077384	4.870357	-0.497737
H	1.766551	3.970144	-1.870077
H	2.776871	5.117650	-0.961330
H	4.574385	-2.477832	0.678527
H	4.402499	-2.505244	-1.091822
H	3.784834	-3.870795	-0.116345

TS[**5**•H–**1**•OS_3]
E = –4473.172909 a.u.
N_{imag} = 1

Cu	1.096893	-0.070256	0.038606
P	-0.069682	0.242272	-1.851146
P	-0.152276	-0.323095	1.854656
O	-1.988335	-1.147716	-0.246738
C	-1.102865	-1.150977	-2.433103
C	-1.035247	-1.738812	-3.703388
H	-0.378298	-1.308851	-4.459085
C	-1.787148	-2.881406	-3.986695
H	-1.721858	-3.337385	-4.974959
C	-2.620666	-3.450307	-3.018053
H	-3.194231	-4.343593	-3.263474
C	-2.726473	-2.880762	-1.743559
C	-1.955906	-1.743263	-1.494629
C	-3.650203	-3.362440	-0.621604
C	-4.094222	-4.814075	-0.820478
H	-3.238336	-5.502383	-0.835405
H	-4.645115	-4.921792	-1.763969
H	-4.776500	-5.122542	-0.017552
C	-4.903456	-2.445456	-0.606752
H	-4.621093	-1.393127	-0.472358
H	-5.572381	-2.732849	0.216771
H	-5.448970	-2.537694	-1.556592
C	-2.897781	-3.163116	0.697224
C	-2.101509	-2.020340	0.819393
C	-1.356542	-1.702621	1.962108
C	-1.463569	-2.569895	3.059469
H	-0.895368	-2.362446	3.965616
C	-2.263856	-3.710421	2.978992
H	-2.333733	-4.380554	3.836208
C	-2.963565	-4.012927	1.806479
H	-3.565932	-4.919723	1.761393
C	0.907488	0.667336	-3.331446
C	2.249206	0.258785	-3.345316
H	2.666903	-0.219276	-2.453754
C	3.044595	0.527406	-4.461301
H	4.091696	0.221962	-4.464046
C	2.509731	1.207804	-5.557300
H	3.135640	1.424228	-6.424292
C	1.175285	1.629078	-5.538220
H	0.761455	2.171549	-6.389516
C	0.373944	1.361599	-4.428163
H	-0.664311	1.696541	-4.406038
C	-1.261462	1.621095	-1.704846
C	-2.592615	1.540196	-2.132579
H	-2.962601	0.619076	-2.584287
C	-3.447112	2.634684	-1.979728
H	-4.484837	2.560837	-2.308598
C	-2.974641	3.819087	-1.412172
H	-3.643270	4.672181	-1.290222
C	-1.646761	3.903576	-0.983642
H	-1.276799	4.818803	-0.521799
C	-0.797017	2.808431	-1.117104
H	0.229319	2.862227	-0.750060
C	0.727897	-0.442678	3.450969
C	1.984303	-1.063308	3.449023
H	2.403132	-1.414998	2.504408
C	2.703303	-1.200881	4.637524
H	3.684715	-1.675983	4.624417
C	2.176540	-0.706769	5.832941
H	2.743940	-0.800689	6.759778
C	0.926327	-0.079318	5.839838
H	0.516826	0.313172	6.771653
C	0.201619	0.050238	4.654966
H	-0.770306	0.544958	4.658086
C	-1.145275	1.200782	2.045376
C	-0.460245	2.380507	2.382151
H	0.614845	2.351070	2.560938
C	-1.145968	3.588113	2.486232
H	-0.601276	4.494296	2.754131
C	-2.519080	3.638972	2.229451
H	-3.054007	4.587050	2.295223
C	-3.199670	2.474766	1.870343
H	-4.267127	2.510763	1.650532
C	-2.519825	1.258447	1.784621
H	-3.063415	0.354742	1.513222

TS[**5**•H–**1**•OS_3] (cont)
E = –4473.172909 a.u.
N_{imag} = 1

C	3.795995	-1.477508	-0.015956
O	3.040099	-0.314746	-0.215744
H	3.230745	-2.363098	-0.371497
C	5.138278	-1.416617	-0.745361
H	3.990743	-1.658926	1.066691
Si	3.275130	1.800744	0.491917
H	1.746931	1.533140	0.435096
C	4.127488	2.146231	-1.155628
H	4.819316	2.994945	-1.044017
H	4.670114	1.280855	-1.548209
H	3.374152	2.433914	-1.903867
C	4.304678	1.157276	1.938607
H	5.060840	1.916592	2.191845
H	4.813976	0.204320	1.755009
H	3.665625	1.033092	2.824868
H	2.485408	3.621580	2.101801
H	3.887581	4.188166	1.175346
C	2.953391	3.607127	1.104172
H	2.279892	4.147092	0.419124
H	5.749774	-0.583020	-0.371495
H	4.980654	-1.263303	-1.823254
H	5.706325	-2.349766	-0.608219

1•OS_3

E = –4473.196144 a.u.

N_{imag} = 0

Cu	1.324161	0.478438	0.136067
P	0.044707	0.761764	-1.688411
P	0.116906	0.235354	2.002744
O	-2.004160	-0.233157	0.095794
C	-1.328485	-0.367274	-2.166448
C	-1.515712	-0.919754	-3.441955
H	-0.875361	-0.601012	-4.263121
C	-2.495432	-1.890801	-3.656695
H	-2.622771	-2.316832	-4.652402
C	-3.312735	-2.323856	-2.608836
H	-4.064653	-3.089634	-2.796130
C	-3.180140	-1.773429	-1.329114
C	-2.192026	-0.800601	-1.151788
C	-4.058071	-2.117121	-0.124136
C	-4.765995	-3.464461	-0.291239
H	-4.047692	-4.287391	-0.407553
H	-5.423887	-3.448482	-1.169940
H	-5.401880	-3.676284	0.578226
C	-5.123845	-0.999139	0.032338
H	-4.649690	-0.015636	0.144421
H	-5.743500	-1.189235	0.920128
H	-5.772665	-0.969742	-0.854556
C	-3.152020	-2.091649	1.108271
C	-2.172813	-1.096663	1.167413
C	-1.284940	-0.929201	2.239507
C	-1.414883	-1.818678	3.317740
H	-0.738218	-1.733815	4.167208
C	-2.382179	-2.824361	3.292629
H	-2.467090	-3.510602	4.135935
C	-3.240619	-2.965288	2.197999
H	-3.983851	-3.761745	2.197998
C	0.973530	0.860613	-3.259795
C	1.658469	-0.289664	-3.689078
H	1.609223	-1.197964	-3.086948
C	2.413993	-0.266304	-4.859632
H	2.936272	-1.167172	-5.185186
C	2.521399	0.914620	-5.601949
H	3.123312	0.936982	-6.511102
C	1.860403	2.065887	-5.169056
H	1.943748	2.991286	-5.740757
C	1.082775	2.039105	-4.008345
H	0.559375	2.937280	-3.679689
C	-0.807008	2.374028	-1.551216
C	-2.086272	2.602781	-2.076013
H	-2.607956	1.801895	-2.600988
C	-2.695446	3.848910	-1.919453
H	-3.695576	4.017023	-2.321876
C	-2.025612	4.877350	-1.252055
H	-2.503844	5.849956	-1.127692
C	-0.748043	4.654048	-0.733083
H	-0.227807	5.448048	-0.197624
C	-0.142255	3.405770	-0.870935
H	0.839548	3.202652	-0.436416
C	1.058560	-0.002452	3.552564
C	2.272778	-0.694539	3.475736
H	2.631675	-1.020690	2.499508
C	3.028277	-0.930606	4.625536
H	3.977039	-1.463795	4.552386
C	2.576061	-0.464745	5.862390
H	3.167883	-0.639548	6.761894
C	1.369854	0.239042	5.945323
H	1.020291	0.611816	6.909200
C	0.613296	0.470542	4.796000
H	-0.322840	1.027145	4.857550
C	-0.624398	1.890174	2.254284
C	0.259602	2.952297	2.514422
H	1.331076	2.755213	2.563638
C	-0.229186	4.245341	2.687418
H	0.464987	5.059806	2.899870
C	-1.598513	4.501983	2.570315
H	-1.978442	5.517336	2.690382
C	-2.476613	3.454609	2.288709
H	-3.544961	3.648418	2.185513
C	-1.994818	2.152054	2.141857
H	-2.692125	1.339943	1.942757
C	2.047222	-2.655330	-0.058192

1•OS_3 (cont)

E = –4473.196144 a.u.

N_{imag} = 0

O	2.516912	-1.390352	-0.561965
H	2.162366	-2.673732	1.039927
C	0.592248	-2.848947	-0.431796
H	2.665492	-3.468340	-0.473879
Si	4.180116	-1.072746	-0.720218
H	2.428168	1.546583	0.485195
C	4.946977	-0.883444	0.981696
C	4.394527	0.479787	-1.726876
C	4.958286	-2.541279	-1.610632
H	4.788645	-1.772380	1.610228
H	4.513647	-0.009334	1.487446
H	6.033335	-0.729246	0.896613
H	3.813567	1.292233	-1.269127
H	4.067341	0.353182	-2.766234
H	5.459251	0.761107	-1.730469
H	4.433788	-2.756453	-2.553388
H	4.969618	-3.459792	-1.006842
H	6.003095	-2.303310	-1.861919
H	0.456314	-2.859622	-1.521485
H	-0.007847	-2.029599	-0.019123
H	0.214756	-3.795169	-0.019419

1•O

E = –4450.351213 a.u.

N_{imag} = 0

Cu	1.089813	-0.122978	-0.336672
P	-0.219562	0.217441	-2.183116
P	-0.286621	-0.345520	1.476077
O	-1.820411	-1.548170	-0.697314
C	-0.968867	-1.298077	-2.875594
C	-0.810999	-1.795484	-4.174497
H	-0.271349	-1.206238	-4.915749
C	-1.315753	-3.056312	-4.503124
H	-1.182282	-3.443860	-5.513522
C	-1.980094	-3.833425	-3.548378
H	-2.348724	-4.820338	-3.826513
C	-2.178564	-3.355426	-2.247129
C	-1.673203	-2.087153	-1.960386
C	-2.937293	-4.080985	-1.133501
C	-3.005545	-5.591527	-1.376053
H	-2.003293	-6.039165	-1.416004
H	-3.522639	-5.807531	-2.320087
H	-3.577842	-6.085975	-0.580428
C	-4.378599	-3.506150	-1.081591
H	-4.363380	-2.420839	-0.917314
H	-4.942678	-3.973553	-0.261996
H	-4.898736	-3.703770	-2.029681
C	-2.243272	-3.741902	0.189831
C	-1.730856	-2.450258	0.345680
C	-1.096438	-1.994310	1.505936
C	-1.012302	-2.884513	2.584576
H	-0.521976	-2.567679	3.504357
C	-1.519908	-4.179467	2.469088
H	-1.439207	-4.867050	3.311403
C	-2.117968	-4.608875	1.280517
H	-2.492750	-5.629423	1.209109
C	0.770692	0.866091	-3.571495
C	1.934380	0.156274	-3.922171
H	2.163117	-0.780348	-3.408956
C	2.786778	0.645593	-4.910171
H	3.683425	0.084937	-5.177599
C	2.501368	1.858881	-5.545728
H	3.175269	2.247118	-6.310264
C	1.354827	2.573493	-5.193510
H	1.128528	3.520616	-5.685326
C	0.489278	2.079461	-4.213880
H	-0.408011	2.637230	-3.944531
C	-1.638684	1.362348	-2.076698
C	-2.793675	1.219790	-2.858563
H	-2.869122	0.392872	-3.565914
C	-3.845356	2.127975	-2.725648
H	-4.745572	2.006746	-3.330066
C	-3.744395	3.191662	-1.824313
H	-4.568173	3.899082	-1.720601
C	-2.594580	3.337746	-1.044906
H	-2.516152	4.151104	-0.324428
C	-1.553064	2.418253	-1.159238
H	-0.675014	2.503385	-0.519322
C	0.662880	-0.377141	3.038699
C	1.939178	-0.959015	2.972854
H	2.282685	-1.376552	2.025450
C	2.773668	-0.961584	4.088211
H	3.773734	-1.388839	4.007792
C	2.338036	-0.384334	5.285608
H	2.995283	-0.367984	6.156072
C	1.059847	0.174804	5.363675
H	0.714284	0.620674	6.297572
C	0.220960	0.176005	4.246439
H	-0.769602	0.625849	4.309087
C	-1.664111	0.814140	1.797825
C	-1.358204	2.123420	2.208452
H	-0.324777	2.407841	2.397577
C	-2.370859	3.062921	2.387516
H	-2.109684	4.068864	2.719303
C	-3.701721	2.722701	2.129234
H	-4.493490	3.461941	2.256799
C	-4.010361	1.431358	1.697511
H	-5.044722	1.157907	1.485238
C	-3.001495	0.479565	1.541118
H	-3.259784	-0.528961	1.221702
H	1.574764	-1.597345	-0.505031

1•O (cont)
E = –4450.351213 a.u.
N_{imag} = 0

C	1.936823	3.234284	3.165819
C	1.299783	4.455118	2.913724
C	0.956300	4.781726	1.599080
C	1.249503	3.901844	0.558686
C	1.890173	2.665408	0.792693
C	2.223805	2.352468	2.127845
H	2.200007	2.952893	4.185948
H	1.073075	5.139701	3.731537
H	0.458717	5.727902	1.380838
H	0.985789	4.170651	-0.466193
C	2.160412	1.806798	-0.365399
C	3.022065	0.710041	-0.505233
H	1.802657	2.233995	-1.305825
H	2.697484	1.403929	2.359282
C	4.077619	0.239810	0.426351
H	3.194155	0.401891	-1.540797
C	4.365018	-1.129348	0.564092
C	6.174994	-0.642558	2.098362
C	4.889451	1.158457	1.113910
C	5.917527	0.722161	1.950797
H	6.525456	1.455025	2.483491
H	4.708366	2.225769	0.988680
C	5.400746	-1.566014	1.388245
H	3.725123	-1.841229	0.039613
H	6.976984	-0.983814	2.754344
H	5.596897	-2.634937	1.486808

TS[**1**•O–**6**]
E = –4450.338797 a.u.
N_{imag} = 1

Cu	0.941610	-0.104965	-0.334442
P	-0.295522	0.183699	-2.181056
P	-0.284342	-0.367824	1.506936
O	-2.068763	-1.341394	-0.608487
C	-1.284666	-1.213898	-2.823721
C	-1.253614	-1.731706	-4.124858
H	-0.669623	-1.224376	-4.892626
C	-1.943425	-2.909666	-4.422679
H	-1.909173	-3.312959	-5.435158
C	-2.670677	-3.583814	-3.435998
H	-3.187988	-4.508013	-3.691721
C	-2.744830	-3.080234	-2.131119
C	-2.053858	-1.895725	-1.874440
C	-3.552035	-3.688080	-0.981936
C	-3.851130	-5.172156	-1.212633
H	-2.928177	-5.762347	-1.293992
H	-4.435293	-5.309132	-2.131956
H	-4.454174	-5.578091	-0.390073
C	-4.890321	-2.908878	-0.869409
H	-4.710046	-1.837576	-0.712087
H	-5.479456	-3.289780	-0.023194
H	-5.475237	-3.028020	-1.792448
C	-2.756781	-3.454870	0.306768
C	-2.060394	-2.248444	0.434266
C	-1.317128	-1.889242	1.564648
C	-1.312595	-2.787152	2.641325
H	-0.744441	-2.545228	3.538685
C	-2.002026	-3.997182	2.552593
H	-1.982539	-4.691010	3.393421
C	-2.707479	-4.334092	1.393658
H	-3.227536	-5.290079	1.342907
C	0.716225	0.671505	-3.619133
C	1.770224	-0.182282	-3.991432
H	1.910385	-1.123762	-3.455755
C	2.629763	0.170863	-5.030374
H	3.441610	-0.500282	-5.313556
C	2.460598	1.389394	-5.696447
H	3.140697	1.671205	-6.501062
C	1.423327	2.246249	-5.323166
H	1.289600	3.199005	-5.837354
C	0.550847	1.889364	-4.291561
H	-0.259330	2.559211	-4.002957
C	-1.527990	1.521823	-2.019514
C	-2.738058	1.528067	-2.726574
H	-2.975690	0.704357	-3.401096
C	-3.639440	2.581117	-2.560190
H	-4.584713	2.576523	-3.104963
C	-3.330410	3.639323	-1.701101
H	-4.036443	4.460718	-1.571691
C	-2.123702	3.636848	-0.997342
H	-1.883633	4.447704	-0.310647
C	-1.231654	2.575930	-1.143818
H	-0.307285	2.548595	-0.565855
C	0.660344	-0.513472	3.065086
C	1.884580	-1.193842	2.998902
H	2.220437	-1.591724	2.040630
C	2.687348	-1.319137	4.130759
H	3.649455	-1.826897	4.054212
C	2.271424	-0.761140	5.343447
H	2.905357	-0.839882	6.227628
C	1.045912	-0.093639	5.418976
H	0.719126	0.343635	6.363431
C	0.238418	0.027069	4.286051
H	-0.710307	0.559966	4.345883
C	-1.467741	0.988127	1.829615
C	-0.951163	2.231205	2.233535
H	0.117447	2.351827	2.404556
C	-1.803835	3.315180	2.429085
H	-1.382417	4.267890	2.752230
C	-3.176044	3.183496	2.198952
H	-3.841409	4.036124	2.340847
C	-3.690961	1.956061	1.777328
H	-4.759367	1.846470	1.587445
C	-2.844238	0.860661	1.600068
H	-3.260577	-0.094964	1.285357
H	2.111324	-1.144826	-0.603698

TS[**1**•O–**6**] (cont)
E = –4450.338797 a.u.
N_{imag} = 1

C	2.520707	2.779840	3.118634
C	2.033481	4.086239	2.984891
C	1.671580	4.537823	1.710556
C	1.783233	3.700258	0.603937
C	2.256534	2.368658	0.718128
C	2.631767	1.939965	2.014945
H	2.807054	2.399681	4.100531
H	1.942620	4.738320	3.853731
H	1.297791	5.554853	1.577052
H	1.502225	4.072282	-0.383934
C	2.321435	1.546748	-0.480974
C	2.994654	0.286896	-0.672413
H	2.069677	2.076408	-1.402247
H	2.996416	0.928590	2.163127
C	4.139635	-0.175703	0.183087
H	3.191516	0.096319	-1.732972
C	4.318375	-1.512447	0.560435
C	6.392086	-0.969744	1.684016
C	5.123040	0.756011	0.554247
C	6.234168	0.364763	1.300885
H	6.977868	1.108782	1.589942
H	4.995497	1.798995	0.264080
C	5.432800	-1.910106	1.301127
H	3.555081	-2.239299	0.272851
H	7.256966	-1.274118	2.275062
H	5.547405	-2.957440	1.585367

6
E = –4450.376331 a.u.
N_{imag} = 0

Cu	0.720526	-0.186918	-0.195193
P	-0.515711	0.152156	-2.035937
P	-0.524425	-0.367790	1.657122
O	-2.293441	-1.379591	-0.411192
C	-1.404667	-1.346098	-2.595747
C	-1.290824	-1.941203	-3.858677
H	-0.681431	-1.462118	-4.624670
C	-1.936921	-3.151360	-4.121148
H	-1.836964	-3.613087	-5.103756
C	-2.709978	-3.779649	-3.138795
H	-3.199927	-4.725270	-3.368523
C	-2.864976	-3.202664	-1.872733
C	-2.201164	-1.995524	-1.645092
C	-3.743650	-3.748160	-0.743030
C	-4.029923	-5.243306	-0.907838
H	-3.104891	-5.835618	-0.897691
H	-4.555912	-5.433380	-1.852418
H	-4.683734	-5.603031	-0.102754
C	-5.084294	-2.964936	-0.760928
H	-4.912069	-1.886574	-0.648464
H	-5.728918	-3.301072	0.063445
H	-5.607353	-3.133969	-1.712839
C	-3.031357	-3.441982	0.577889
C	-2.334920	-2.233444	0.672987
C	-1.623117	-1.827232	1.808749
C	-1.686511	-2.656632	2.937941
H	-1.151206	-2.371898	3.843102
C	-2.397813	-3.855996	2.888875
H	-2.435698	-4.496306	3.770492
C	-3.046769	-4.255625	1.715704
H	-3.573184	-5.209301	1.694624
C	0.429811	0.641911	-3.519124
C	1.637297	-0.033008	-3.764491
H	1.956927	-0.818715	-3.077527
C	2.424616	0.309890	-4.862743
H	3.360112	-0.220102	-5.045805
C	2.024503	1.344125	-5.714709
H	2.647988	1.623379	-6.564868
C	0.829803	2.025170	-5.469166
H	0.516550	2.834794	-6.129757
C	0.030499	1.673698	-4.378986
H	-0.901966	2.205428	-4.188964
C	-1.838074	1.406338	-1.927400
C	-3.137025	1.191157	-2.408055
H	-3.391364	0.241690	-2.880201
C	-4.104314	2.190032	-2.285300
H	-5.115114	2.012312	-2.655388
C	-3.777131	3.415060	-1.699414
H	-4.533396	4.195211	-1.604457
C	-2.481803	3.633626	-1.223724
H	-2.223407	4.581319	-0.751734
C	-1.519320	2.630800	-1.321364
H	-0.516682	2.791750	-0.919947
C	0.445751	-0.442260	3.206014
C	1.430186	-1.441468	3.282387
H	1.531851	-2.156556	2.463833
C	2.284698	-1.511524	4.380495
H	3.047411	-2.289876	4.424608
C	2.178019	-0.570107	5.409814
H	2.860908	-0.609012	6.259182
C	1.196182	0.420047	5.343666
H	1.105411	1.154232	6.145251
C	0.326414	0.480287	4.251848
H	-0.434814	1.258137	4.205597
C	-1.616184	1.077469	1.853295
C	-1.005138	2.339675	1.944054
H	0.081810	2.425998	1.906239
C	-1.783639	3.485595	2.085084
H	-1.292112	4.455545	2.170618
C	-3.178413	3.392438	2.083404
H	-3.788741	4.291849	2.173302
C	-3.789119	2.144214	1.946130
H	-4.876870	2.066858	1.926170
C	-3.013801	0.987280	1.845436
H	-3.497487	0.013782	1.764782
H	3.232435	-1.033251	1.529628

6 (cont)
E = –4450.376331 a.u.
N_{imag} = 0

C	2.409190	3.088876	2.139703
C	2.010443	4.114118	1.272640
C	1.865720	3.821575	-0.089636
C	2.118167	2.540046	-0.572246
C	2.517636	1.479417	0.288172
C	2.647602	1.801136	1.665721
H	2.533001	3.293829	3.204919
H	1.829682	5.121616	1.648006
H	1.569697	4.607770	-0.787400
H	2.033957	2.336728	-1.642649
C	2.722315	0.123465	-0.245446
C	3.644796	-0.811188	0.535712
H	3.013550	0.173757	-1.303809
H	2.948721	1.025787	2.368825
C	5.051108	-0.276853	0.724315
H	3.687765	-1.778161	0.008178
C	5.594104	-0.118500	2.005828
C	7.644259	0.776473	1.084588
C	5.832428	0.105809	-0.375678
C	7.115897	0.623086	-0.201310
H	7.707613	0.912826	-1.071487
H	5.419509	0.006424	-1.381760
C	6.876909	0.404135	2.189600
H	4.993491	-0.404093	2.873205
H	8.645221	1.187594	1.222413
H	7.275169	0.526690	3.198377

6•R

E = –4605.679172 a.u.

N_{imag} = 0

Cu	0.689386	0.576331	-0.272016
P	-0.537595	0.786976	-2.142607
P	-0.569007	0.233915	1.577520
O	-1.842712	-1.203392	-0.516403
C	-1.121049	-0.850732	-2.732784
C	-0.911305	-1.356041	-4.023258
H	-0.468464	-0.719227	-4.787381
C	-1.229915	-2.681653	-4.318724
H	-1.056677	-3.064973	-5.324658
C	-1.756037	-3.527299	-3.337676
H	-1.984432	-4.561695	-3.591352
C	-1.983000	-3.060842	-2.039130
C	-1.661078	-1.725878	-1.781332
C	-2.609503	-3.882041	-0.909280
C	-2.423345	-5.386796	-1.127014
H	-1.359470	-5.656623	-1.162776
H	-2.897507	-5.705039	-2.064185
H	-2.902066	-5.957070	-0.320678
C	-4.127049	-3.554418	-0.868160
H	-4.291975	-2.481242	-0.704389
H	-4.611198	-4.105760	-0.049614
H	-4.601894	-3.836891	-1.818477
C	-1.996505	-3.418330	0.414051
C	-1.667448	-2.068615	0.544412
C	-1.141124	-1.499047	1.711026
C	-0.991297	-2.333068	2.825999
H	-0.603116	-1.924722	3.758045
C	-1.316018	-3.687522	2.733412
H	-1.187092	-4.332512	3.603001
C	-1.800968	-4.227810	1.538616
H	-2.034526	-5.290613	1.489979
C	0.280955	1.497830	-3.618165
C	1.656034	1.751488	-3.566017
H	2.195240	1.534786	-2.643419
C	2.315688	2.296880	-4.670302
H	3.386851	2.495022	-4.617909
C	1.601623	2.596334	-5.831489
H	2.114378	3.025389	-6.693358
C	0.223459	2.358749	-5.885548
H	-0.338787	2.603636	-6.787680
C	-0.435866	1.816502	-4.783715
H	-1.513352	1.650158	-4.818728
C	-2.049878	1.816170	-2.078367
C	-3.333377	1.278948	-1.921506
H	-3.469019	0.201435	-1.844986
C	-4.445255	2.121257	-1.874387
H	-5.439752	1.691974	-1.747875
C	-4.288748	3.503243	-1.994213
H	-5.160805	4.157554	-1.967028
C	-3.008168	4.045051	-2.135193
H	-2.874709	5.124668	-2.216893
C	-1.893151	3.209993	-2.163103
H	-0.894914	3.639214	-2.258014
C	0.084479	0.610135	3.251370
C	1.203616	-0.088857	3.736852
H	1.624569	-0.907239	3.155771
C	1.782307	0.252221	4.958253
H	2.647061	-0.307317	5.316662
C	1.263376	1.310037	5.710046
H	1.723218	1.585316	6.659797
C	0.151492	2.009551	5.237622
H	-0.264746	2.833853	5.818168
C	-0.439222	1.659697	4.021703
H	-1.306675	2.213384	3.665147
C	-2.115192	1.200709	1.544535
C	-2.040800	2.532100	1.109261
H	-1.093074	2.926646	0.733246
C	-3.174039	3.343051	1.158818
H	-3.108644	4.378345	0.825900
C	-4.392422	2.823045	1.601744
H	-5.280751	3.455616	1.621305
C	-4.475947	1.487477	2.002962
H	-5.428152	1.075141	2.339892
C	-3.337643	0.679802	1.987175
H	-3.395385	-0.356743	2.321518
H	2.754701	0.704079	1.899839

6•R (cont)

E = –4605.679172 a.u.

N_{imag} = 0

C	1.615243	-3.248710	0.432677
C	1.289072	-3.693195	-0.851553
C	1.532616	-2.848007	-1.940581
C	2.044763	-1.570843	-1.746201
C	2.338042	-1.069367	-0.446014
C	2.137328	-1.974216	0.630484
H	1.455640	-3.900219	1.292953
H	0.873837	-4.689099	-1.005346
H	1.301504	-3.182434	-2.952465
H	2.222578	-0.925433	-2.609140
C	2.729928	0.336813	-0.245563
C	3.444901	0.720086	1.042066
H	3.237181	0.737473	-1.132312
H	2.428054	-1.666535	1.632999
C	4.665148	-0.096414	1.433832
H	3.751047	1.776593	0.960094
C	5.395417	-0.843462	0.501892
C	6.922141	-1.588916	2.230303
C	5.082472	-0.115113	2.773195
C	6.199739	-0.850314	3.171876
H	6.506286	-0.851046	4.219528
H	4.515811	0.459648	3.510309
C	6.514442	-1.581538	0.894412
H	5.068065	-0.860874	-0.538161
H	7.792326	-2.170687	2.537340
H	7.066197	-2.162071	0.153133
C	1.582998	3.674623	1.627859
O	1.164910	3.500529	0.264294
H	2.663427	3.907584	1.666363
C	0.782735	4.805356	2.245571
H	1.427018	2.744198	2.198546
H	1.492533	2.612406	-0.032390
H	-0.287526	4.558188	2.272805
H	0.909199	5.731535	1.667281
H	1.118340	4.989762	3.276057

TS[**6**•R–**5**•Q]

E = –4605.657423 a.u.

N_{imag} = 1

Cu	0.793859	1.087938	-0.052328
P	-0.394016	1.302431	-1.904420
P	-0.338338	0.768663	1.838529
O	-1.948074	-0.480213	-0.262467
C	-1.161236	-0.253950	-2.480070
C	-0.997464	-0.796811	-3.761147
H	-0.484528	-0.217263	-4.528176
C	-1.449118	-2.088479	-4.037010
H	-1.300620	-2.511057	-5.030784
C	-2.076901	-2.851280	-3.048372
H	-2.403144	-3.863919	-3.281505
C	-2.285600	-2.329677	-1.766838
C	-1.818110	-1.036920	-1.522917
C	-3.007325	-3.044808	-0.623352
C	-3.066731	-4.559392	-0.839536
H	-2.062902	-4.998997	-0.913709
H	-3.617248	-4.794149	-1.759804
H	-3.603172	-5.046509	-0.015068
C	-4.453130	-2.485148	-0.541675
H	-4.447590	-1.397385	-0.394122
H	-4.989435	-2.946245	0.299581
H	-4.995268	-2.704117	-1.472433
C	-2.278417	-2.673154	0.671190
C	-1.807018	-1.361760	0.798889
C	-1.141136	-0.878412	1.934006
C	-0.963969	-1.771873	3.000498
H	-0.415285	-1.448691	3.882949
C	-1.423591	-3.084624	2.906908
H	-1.261208	-3.771758	3.737355
C	-2.067775	-3.535302	1.751494
H	-2.401423	-4.570491	1.693290
C	0.442354	1.918609	-3.405977
C	1.705280	1.383523	-3.710195
H	2.136616	0.620572	-3.059045
C	2.407100	1.831307	-4.828334
H	3.385600	1.408436	-5.058943
C	1.865256	2.832921	-5.639936
H	2.420684	3.193325	-6.506573
C	0.615435	3.376768	-5.334471
H	0.192140	4.161977	-5.962230
C	-0.098500	2.917976	-4.225399
H	-1.073816	3.343042	-3.987271
C	-1.800138	2.439392	-1.677317
C	-3.071457	2.191577	-2.209147
H	-3.250421	1.278264	-2.777936
C	-4.104613	3.109205	-2.007428
H	-5.095680	2.906906	-2.415717
C	-3.868769	4.283344	-1.289551
H	-4.676488	4.998728	-1.131492
C	-2.601933	4.532332	-0.754898
H	-2.419296	5.435161	-0.173144
C	-1.576110	3.607916	-0.933612
H	-0.596626	3.780642	-0.485714
C	0.563126	0.971317	3.407409
C	1.928909	1.273418	3.341220
H	2.420524	1.366395	2.372504
C	2.653866	1.467814	4.518990
H	3.718629	1.691712	4.459004
C	2.022507	1.364784	5.758316
H	2.593806	1.509641	6.676356
C	0.653220	1.082064	5.826782
H	0.155478	1.013813	6.795079
C	-0.078241	0.895763	4.655653
H	-1.150998	0.703152	4.706221
C	-1.700668	1.976716	2.050739
C	-1.361847	3.250177	2.539320
H	-0.327687	3.465296	2.812405
C	-2.341433	4.228056	2.699728
H	-2.066038	5.207284	3.094041
C	-3.668797	3.954832	2.358405
H	-4.435266	4.720858	2.480716
C	-4.006426	2.698993	1.851475
H	-5.037402	2.481792	1.570316
C	-3.030598	1.712836	1.699372
H	-3.310504	0.736266	1.309606
H	0.613523	-1.499204	-0.428307

TS[**6**•R–**5**•Q] (cont)

E = –4605.657423 a.u.

N_{imag} = 1

C	2.565200	-2.205992	3.256547
C	3.763779	-1.739830	3.798917
C	4.602150	-0.955231	2.997521
C	4.240111	-0.638279	1.691492
C	3.028342	-1.092558	1.125052
C	2.206620	-1.893964	1.945620
H	1.901489	-2.831807	3.856639
H	4.044433	-1.985361	4.823308
H	5.548076	-0.584855	3.398026
H	4.897980	-0.011455	1.084789
C	2.634585	-0.676213	-0.237869
C	1.595151	-1.573097	-0.922329
H	3.531662	-0.601515	-0.877831
H	1.275348	-2.293060	1.548418
C	1.948863	-3.048348	-0.986974
H	1.420627	-1.215575	-1.950052
C	0.978933	-4.022993	-0.722768
C	2.589625	-5.792895	-1.074462
C	3.248096	-3.472264	-1.291334
C	3.567438	-4.829495	-1.338456
H	4.588615	-5.137505	-1.569327
H	4.023737	-2.724864	-1.466726
C	1.290781	-5.383160	-0.763793
H	-0.030150	-3.701445	-0.457359
H	2.843034	-6.853607	-1.097960
H	0.523569	-6.126338	-0.537404
C	3.263355	2.792957	-0.789333
O	2.663075	1.989799	0.216607
H	4.260978	3.115907	-0.436423
C	2.414572	4.012648	-1.113908
H	3.423093	2.198733	-1.710733
H	2.682809	0.833933	-0.063766
H	1.451674	3.705825	-1.546290
H	2.217169	4.595745	-0.202791
H	2.916797	4.663817	-1.845024

5•Q

E = –4605.709552 a.u.

N_{imag} = 0

Cu	1.248900	1.230383	0.029088
P	0.021181	1.479139	-1.797179
P	0.026439	0.953036	1.904021
O	-1.775131	-0.025274	-0.125457
C	-0.941800	0.018031	-2.337549
C	-0.870790	-0.575529	-3.605788
H	-0.256458	-0.116713	-4.379989
C	-1.559607	-1.762189	-3.861964
H	-1.478565	-2.229346	-4.843514
C	-2.340024	-2.369244	-2.872871
H	-2.851119	-3.305698	-3.091043
C	-2.454814	-1.790573	-1.606173
C	-1.739262	-0.612985	-1.378699
C	-3.317221	-2.305501	-0.451176
C	-3.759507	-3.754724	-0.655400
H	-2.898650	-4.426353	-0.758755
H	-4.378931	-3.839234	-1.558319
H	-4.375418	-4.091360	0.189225
C	-4.578342	-1.404320	-0.351894
H	-4.306423	-0.349602	-0.217807
H	-5.196087	-1.715606	0.502286
H	-5.174638	-1.492584	-1.270961
C	-2.487140	-2.114436	0.821063
C	-1.761765	-0.924727	0.929775
C	-0.952288	-0.591055	2.023570
C	-0.843605	-1.545154	3.046543
H	-0.179528	-1.360278	3.887969
C	-1.550544	-2.746081	2.968281
H	-1.445741	-3.480277	3.767130
C	-2.376584	-3.025091	1.875001
H	-2.914150	-3.971597	1.833529
C	0.863199	1.957173	-3.349220
C	2.167451	1.483669	-3.551788
H	2.649370	0.894622	-2.769454
C	2.852504	1.785983	-4.728449
H	3.868094	1.416209	-4.874142
C	2.244365	2.578745	-5.705602
H	2.783318	2.826641	-6.620881
C	0.949702	3.065430	-5.503061
H	0.476727	3.691962	-6.260538
C	0.258268	2.754116	-4.330918
H	-0.751053	3.135617	-4.171914
C	-1.257097	2.766590	-1.587980
C	-2.571062	2.624593	-2.052154
H	-2.868316	1.708479	-2.563728
C	-3.498663	3.649596	-1.855428
H	-4.522265	3.528904	-2.212953
C	-3.117448	4.824426	-1.204654
H	-3.843751	5.622598	-1.047366
C	-1.807970	4.969697	-0.738856
H	-1.511313	5.876405	-0.212339
C	-0.884637	3.942469	-0.919285
H	0.130719	4.040723	-0.530561
C	0.922834	1.080812	3.491016
C	2.295529	1.365522	3.446270
H	2.792348	1.476768	2.473449
C	3.010029	1.509054	4.639659
H	4.078915	1.723212	4.602515
C	2.364685	1.374327	5.869942
H	2.928291	1.479011	6.798208
C	0.989258	1.117444	5.914552
H	0.478859	1.031369	6.874849
C	0.267701	0.982314	4.729983
H	-0.809264	0.811574	4.764189
C	-1.193501	2.308942	2.121885
C	-0.685521	3.553347	2.534285
H	0.380908	3.659583	2.739543
C	-1.536200	4.643399	2.703971
H	-1.128514	5.598843	3.037231
C	-2.903510	4.513606	2.445919
H	-3.569663	5.367678	2.572641
C	-3.410430	3.285554	2.019394
H	-4.474978	3.176562	1.808898
C	-2.563562	2.186821	1.861436
H	-2.979425	1.233030	1.543705
H	1.238994	-0.999115	-0.483629

5•Q (cont)

E = –4605.709552 a.u.

N_{imag} = 0

C	2.669864	-2.088082	3.248443
C	2.646950	-3.464783	3.491478
C	2.857580	-4.355840	2.436189
C	3.072093	-3.873006	1.142781
C	3.075871	-2.495760	0.885745
C	2.883960	-1.609952	1.955587
H	2.526691	-1.381323	4.067256
H	2.477643	-3.841595	4.501443
H	2.849444	-5.431670	2.618417
H	3.216461	-4.573231	0.317892
C	3.180105	-1.947922	-0.512623
C	1.781751	-1.708636	-1.137751
H	3.748453	-2.635094	-1.157770
H	2.921483	-0.539276	1.746104
C	0.982389	-2.966864	-1.309284
H	1.903936	-1.212599	-2.111619
C	0.304315	-3.542233	-0.225186
C	-0.355932	-5.428056	-1.594034
C	0.961731	-3.635509	-2.540431
C	0.295057	-4.852675	-2.688062
H	0.286077	-5.354502	-3.656873
H	1.473963	-3.188542	-3.395008
C	-0.347735	-4.766885	-0.363244
H	0.305069	-3.034456	0.739042
H	-0.867089	-6.386072	-1.700733
H	-0.848873	-5.206248	0.500049
C	4.029598	1.944631	-0.425058
O	3.083820	1.384420	0.439819
H	5.013122	1.982726	0.089481
C	3.662696	3.356653	-0.884418
H	4.192360	1.311068	-1.329515
H	3.698502	-0.978326	-0.485669
H	2.710121	3.338571	-1.436822
H	3.539295	4.015257	-0.011881
H	4.432916	3.786445	-1.545327

1$_2$•PEa

E = –4584.538949 a.u.

N_{imag} = 0

Cu	1.088362	0.753258	-0.200443
P	-0.297641	1.066808	-1.973717
P	-0.231261	0.482646	1.734333
O	-1.996875	-0.470559	-0.251489
C	-1.305976	-0.378332	-2.495589
C	-1.271251	-0.969001	-3.766842
H	-0.689774	-0.503800	-4.561523
C	-1.958786	-2.159952	-4.008582
H	-1.917286	-2.613289	-4.999400
C	-2.708403	-2.771548	-2.999155
H	-3.246683	-3.693157	-3.217391
C	-2.769467	-2.214896	-1.717919
C	-2.041519	-1.041350	-1.505157
C	-3.620496	-2.751779	-0.564439
C	-3.947368	-4.238878	-0.733821
H	-3.034454	-4.847978	-0.746500
H	-4.500470	-4.411560	-1.666167
H	-4.589595	-4.588941	0.084358
C	-4.943188	-1.938293	-0.537694
H	-4.741827	-0.867378	-0.401969
H	-5.577290	-2.276344	0.294238
H	-5.490551	-2.071634	-1.481687
C	-2.877475	-2.477793	0.744433
C	-2.093619	-1.326996	0.826023
C	-1.374732	-0.940800	1.964676
C	-1.553030	-1.712907	3.120038
H	-1.055199	-1.425264	4.044332
C	-2.350821	-2.857687	3.085572
H	-2.473139	-3.455759	3.989133
C	-2.983704	-3.252991	1.904280
H	-3.579826	-4.164751	1.895620
C	0.519786	1.537837	-3.545290
C	1.908159	1.405892	-3.664675
H	2.500843	1.046762	-2.814775
C	2.540743	1.747371	-4.864325
H	3.623626	1.645180	-4.949235
C	1.796692	2.226149	-5.943476
H	2.293970	2.496899	-6.876083
C	0.409622	2.368567	-5.823948
H	-0.176339	2.750135	-6.661336
C	-0.226061	2.028289	-4.631242
H	-1.305645	2.151322	-4.534872
C	-1.506368	2.435094	-1.815813
C	-2.867473	2.247869	-1.549647
H	-3.271730	1.242934	-1.444896
C	-3.717497	3.348563	-1.424283
H	-4.774500	3.189107	-1.208144
C	-3.222041	4.643735	-1.578604
H	-3.890512	5.500649	-1.488093
C	-1.862035	4.837126	-1.837166
H	-1.463447	5.846279	-1.951237
C	-1.006755	3.742708	-1.939644
H	0.056970	3.896532	-2.126480
C	0.617449	0.590232	3.360687
C	1.237977	-0.536061	3.927450
H	1.173453	-1.501103	3.430357
C	1.939671	-0.438807	5.126872
H	2.411705	-1.328225	5.545197
C	2.059120	0.792901	5.773830
H	2.614994	0.871463	6.708858
C	1.471027	1.924561	5.206047
H	1.561669	2.894265	5.697836
C	0.751886	1.824913	4.013400
H	0.284295	2.713847	3.591196
C	-1.401490	1.895290	1.826946
C	-0.975385	3.154299	1.379133
H	0.016654	3.244172	0.930965
C	-1.817795	4.260164	1.492367
H	-1.476862	5.233733	1.140622
C	-3.100350	4.117151	2.024614
H	-3.762794	4.980828	2.096279
C	-3.537835	2.860868	2.450324
H	-4.541775	2.739813	2.860197
C	-2.689927	1.756310	2.361065
H	-3.030599	0.780472	2.707715
H	1.657714	2.186617	0.084810

1$_2$•PEa (cont)

E = –4584.538949 a.u.

N_{imag} = 0

C	0.573161	-3.842596	1.393519
C	-0.130454	-4.606731	0.457203
C	-0.139005	-4.200853	-0.878506
C	0.540957	-3.049792	-1.268989
C	1.260543	-2.270376	-0.339636
C	1.256834	-2.696734	1.002721
H	0.580853	-4.139856	2.442804
H	-0.669724	-5.501290	0.769637
H	-0.682485	-4.778414	-1.627299
H	0.515457	-2.737055	-2.314050
C	1.967236	-1.094328	-0.848461
C	2.944065	-0.252164	-0.283803
H	1.924349	-1.021182	-1.938987
H	1.808680	-2.119610	1.738417
C	3.562154	-0.395031	1.073794
N	3.819312	0.365533	-1.265603
C	4.239437	-1.585445	1.393296
C	5.035948	-0.618235	3.464286
C	3.641506	0.680041	1.972123
C	4.376942	0.572163	3.151239
H	4.412578	1.416038	3.840203
H	3.095239	1.592532	1.731233
C	4.959467	-1.700303	2.583833
H	4.185755	-2.429886	0.705084
H	5.600481	-0.706110	4.393577
H	5.469140	-2.636176	2.818559
C	4.797525	-0.570946	-1.822196
C	4.435610	1.631718	-0.893515
H	5.194581	1.534973	-0.092630
H	3.645622	2.317170	-0.557695
H	4.927264	2.051854	-1.783198
H	4.289640	-1.486266	-2.150339
H	5.588279	-0.847047	-1.094584
H	5.283588	-0.107396	-2.693093

TS[**1**$_2$•PEa–**8a**]

E = –4584.516854 a.u.

N_{imag} = 1

Cu	0.781368	0.828792	-0.105168
P	-0.453723	1.099419	-1.959033
P	-0.459503	0.604877	1.766790
O	-1.889284	-0.754727	-0.260029
C	-1.101742	-0.547813	-2.470185
C	-0.880633	-1.134009	-3.724439
H	-0.375967	-0.565629	-4.504177
C	-1.281683	-2.448935	-3.968239
H	-1.096211	-2.894310	-4.946067
C	-1.918380	-3.199312	-2.975859
H	-2.223805	-4.222338	-3.191636
C	-2.155266	-2.653220	-1.711290
C	-1.723541	-1.341100	-1.498246
C	-2.905512	-3.354670	-0.575707
C	-2.885189	-4.878652	-0.724653
H	-1.858703	-5.264584	-0.693279
H	-3.351935	-5.182437	-1.670664
H	-3.463176	-5.351527	0.079884
C	-4.377099	-2.858366	-0.606033
H	-4.426852	-1.767533	-0.489423
H	-4.946919	-3.317935	0.214054
H	-4.848123	-3.126653	-1.562375
C	-2.288190	-2.902766	0.748940
C	-1.816981	-1.593948	0.833814
C	-1.252760	-1.033217	1.986309
C	-1.243031	-1.820852	3.144155
H	-0.833501	-1.416949	4.068859
C	-1.727806	-3.128875	3.103594
H	-1.704543	-3.739682	4.006473
C	-2.220396	-3.673630	1.914442
H	-2.564270	-4.706997	1.901825
C	0.340120	1.679408	-3.506175
C	-0.426765	2.006213	-4.638403
H	-1.514179	1.934796	-4.596088
C	0.196660	2.430040	-5.810805
H	-0.406168	2.684166	-6.683823
C	1.591262	2.531195	-5.865654
H	2.077869	2.866464	-6.782774
C	2.354661	2.207275	-4.743166
H	3.442090	2.288166	-4.779417
C	1.734384	1.784699	-3.563199
H	2.341589	1.530368	-2.685832
C	-1.938000	2.180680	-1.932567
C	-3.233003	1.707456	-1.690403
H	-3.405369	0.645304	-1.528807
C	-4.312405	2.592933	-1.660962
H	-5.314775	2.210848	-1.464402
C	-4.114498	3.955620	-1.885772
H	-4.961269	4.642717	-1.871202
C	-2.822754	4.436168	-2.119207
H	-2.656044	5.500571	-2.291193
C	-1.741330	3.558220	-2.127987
H	-0.734460	3.939121	-2.306739
C	0.218835	0.942676	3.439455
C	1.203896	0.095530	3.971762
H	1.556337	-0.758451	3.396571
C	1.743263	0.334320	5.234319
H	2.506482	-0.338606	5.625766
C	1.329419	1.442854	5.976346
H	1.757981	1.635798	6.960625
C	0.366635	2.304971	5.447071
H	0.037124	3.174975	6.017035
C	-0.191514	2.053840	4.191879
H	-0.955021	2.723824	3.798329
C	-1.890349	1.743537	1.706544
C	-1.677832	3.045011	1.231362
H	-0.690537	3.316379	0.851551
C	-2.718670	3.972368	1.236645
H	-2.545629	4.982015	0.865205
C	-3.987286	3.598075	1.685097
H	-4.805726	4.318759	1.668991
C	-4.210199	2.295277	2.136461
H	-5.202110	1.996664	2.478837
C	-3.163191	1.372723	2.157098
H	-3.331516	0.359622	2.523992
H	2.050679	1.752366	0.153490

TS[**1**$_2$•PEa–**8a**] (cont)
E = –4584.516854 a.u.
N_{imag} = 1

C	1.240633	-3.736647	1.489671
C	0.824265	-4.670078	0.534200
C	0.803736	-4.282963	-0.808720
C	1.200412	-3.003749	-1.184687
C	1.644962	-2.048298	-0.238130
C	1.633078	-2.456917	1.115766
H	1.245425	-4.005279	2.546929
H	0.514170	-5.671474	0.833638
H	0.476172	-4.984245	-1.578586
H	1.168924	-2.719336	-2.238177
C	2.059282	-0.749620	-0.745633
C	2.846754	0.318755	-0.171414
H	2.031014	-0.699081	-1.837187
H	1.946735	-1.754700	1.882296
C	3.542392	0.170589	1.159224
N	3.701256	0.961279	-1.178375
C	4.351859	-0.959287	1.374327
C	5.130735	-0.045377	3.477154
C	3.551424	1.187139	2.121314
C	4.340259	1.085259	3.267749
H	4.317951	1.884796	4.008959
H	2.908964	2.055120	1.965783
C	5.130556	-1.070326	2.525886
H	4.344556	-1.765070	0.640891
H	5.742241	-0.130968	4.376594
H	5.740668	-1.961570	2.680084
C	4.736310	0.076770	-1.711486
C	4.247894	2.262915	-0.827960
H	4.991933	2.226191	-0.007737
H	3.424830	2.923670	-0.519182
H	4.734987	2.689291	-1.716636
H	4.294082	-0.889783	-1.980104
H	5.562439	-0.100954	-0.993355
H	5.163769	0.534568	-2.615584

8a
E = –4584.56242 a.u.
N_{imag} = 0

Cu	0.661373	0.528183	-0.193090
P	-0.593641	0.760523	-2.041526
P	-0.563369	0.286844	1.695718
O	-1.771121	-1.280799	-0.344786
C	-1.022582	-0.950193	-2.556973
C	-0.736942	-1.483735	-3.821450
H	-0.335953	-0.836740	-4.600460
C	-0.932324	-2.842783	-4.070725
H	-0.693809	-3.249657	-5.053617
C	-1.423981	-3.689069	-3.072349
H	-1.563414	-4.747211	-3.290560
C	-1.741294	-3.191691	-1.803957
C	-1.520491	-1.828286	-1.585975
C	-2.365017	-4.007066	-0.667341
C	-2.107478	-5.507892	-0.832343
H	-1.032570	-5.733771	-0.833331
H	-2.543286	-5.875615	-1.770344
H	-2.581884	-6.072810	-0.019673
C	-3.895223	-3.745422	-0.678369
H	-4.111243	-2.676431	-0.550412
H	-4.377217	-4.294007	0.143265
H	-4.330812	-4.076143	-1.632015
C	-1.801117	-3.473621	0.651056
C	-1.539289	-2.107429	0.741315
C	-1.016415	-1.481090	1.877939
C	-0.802284	-2.275800	3.009053
H	-0.396771	-1.828370	3.914856
C	-1.074857	-3.643792	2.963830
H	-0.889202	-4.257262	3.845369
C	-1.555192	-4.240164	1.796014
H	-1.734042	-5.314667	1.779868
C	0.203447	1.404331	-3.561772
C	1.597513	1.298739	-3.668441
H	2.160554	0.868536	-2.841326
C	2.256309	1.730288	-4.820571
H	3.341760	1.644848	-4.885162
C	1.527104	2.272595	-5.880966
H	2.040135	2.614897	-6.780571
C	0.135536	2.373585	-5.786524
H	-0.439958	2.789585	-6.614682
C	-0.524551	1.939619	-4.636464
H	-1.609647	2.016944	-4.570618
C	-2.180481	1.667535	-2.036182
C	-3.421766	1.036677	-1.880071
H	-3.475963	-0.048945	-1.813989
C	-4.593475	1.792707	-1.819049
H	-5.551896	1.288358	-1.690769
C	-4.544292	3.183259	-1.929074
H	-5.462904	3.769503	-1.891201
C	-3.308211	3.820994	-2.073293
H	-3.257817	4.908041	-2.151939
C	-2.134910	3.071649	-2.107216
H	-1.172372	3.575836	-2.211564
C	0.047186	0.718055	3.364870
C	1.233858	0.115905	3.809953
H	1.744885	-0.607807	3.173962
C	1.771714	0.451496	5.050766
H	2.693066	-0.028484	5.383970
C	1.144721	1.409495	5.853502
H	1.572483	1.681875	6.819283
C	-0.031818	2.017912	5.410516
H	-0.528127	2.765950	6.030469
C	-0.584198	1.670401	4.175323
H	-1.504070	2.148235	3.840217
C	-2.192879	1.117281	1.654723
C	-2.236323	2.451597	1.227239
H	-1.318305	2.930581	0.884359
C	-3.438085	3.156882	1.246622
H	-3.460963	4.194941	0.916143
C	-4.614584	2.525732	1.657788
H	-5.557913	3.073063	1.653593
C	-4.582091	1.188431	2.058877
H	-5.499398	0.688885	2.373995
C	-3.374076	0.488726	2.068822
H	-3.346141	-0.549547	2.401119
H	3.511397	1.321147	-1.446609

8a (cont)
E = –4584.56242 a.u.
N_{imag} = 0

C	3.184837	-2.618081	2.451577
C	2.430343	-3.739036	2.105065
C	1.744967	-3.729332	0.881402
C	1.795298	-2.619515	0.051229
C	2.534541	-1.455058	0.392907
C	3.244716	-1.499002	1.614937
H	3.749879	-2.612257	3.386688
H	2.382748	-4.606786	2.763156
H	1.155621	-4.596962	0.580890
H	1.243091	-2.624845	-0.892527
C	2.511542	-0.320319	-0.541563
C	3.451859	0.887403	-0.417989
H	2.500849	-0.702570	-1.571247
H	3.883199	-0.657788	1.878195
C	2.767992	1.964674	0.424791
N	4.829563	0.624178	0.036719
C	2.937238	2.078684	1.812064
C	1.297838	3.863465	1.911057
C	1.877574	2.854640	-0.214263
C	1.145180	3.789942	0.523007
H	0.467683	4.472573	0.006937
H	1.797675	2.835205	-1.303211
C	2.206112	3.014501	2.546024
H	3.644573	1.418941	2.312424
H	0.719137	4.581564	2.492519
H	2.329908	3.063872	3.628180
C	5.650669	1.823638	-0.072769
C	5.436308	-0.467228	-0.713864
H	5.502630	-0.243991	-1.804008
H	4.857242	-1.387879	-0.583268
H	6.455352	-0.639345	-0.340353
H	5.223954	2.636605	0.528017
H	5.732380	2.182622	-1.125484
H	6.664491	1.613130	0.295717

8a•R

E = –4739.867844 a.u.

N_{imag} = 0

Cu	0.369572	0.115635	-0.199848
P	-0.874827	0.486220	-2.005344
P	-0.906705	-0.058836	1.645424
O	-2.680856	-1.069971	-0.451580
C	-1.776005	-0.977537	-2.630889
C	-1.630112	-1.556075	-3.899053
H	-1.025971	-1.051085	-4.652609
C	-2.225043	-2.788200	-4.178776
H	-2.095619	-3.238071	-5.163605
C	-2.984017	-3.453809	-3.210216
H	-3.431713	-4.417393	-3.450977
C	-3.179969	-2.889623	-1.943646
C	-2.565456	-1.661008	-1.696075
C	-4.045008	-3.480149	-0.827407
C	-4.283246	-4.980295	-1.019009
H	-3.339417	-5.542459	-1.014625
H	-4.799215	-5.170009	-1.969300
H	-4.927971	-5.374549	-0.222804
C	-5.409514	-2.739487	-0.835765
H	-5.271181	-1.658796	-0.703047
H	-6.044696	-3.111091	-0.019203
H	-5.924978	-2.907959	-1.791975
C	-3.341558	-3.176037	0.498068
C	-2.684169	-1.947169	0.617801
C	-1.968571	-1.551661	1.755246
C	-1.983835	-2.422283	2.854776
H	-1.440151	-2.150211	3.758750
C	-2.653704	-3.643484	2.780963
H	-2.650773	-4.313330	3.641314
C	-3.312499	-4.026079	1.608509
H	-3.803679	-4.997367	1.562895
C	0.068558	1.042408	-3.466250
C	1.235294	0.325231	-3.780740
H	1.501915	-0.544294	-3.176461
C	2.046276	0.722254	-4.841814
H	2.946562	0.154120	-5.080474
C	1.714550	1.859671	-5.586427
H	2.357024	2.182567	-6.406442
C	0.563678	2.584860	-5.270544
H	0.303988	3.474512	-5.846067
C	-0.261749	2.176235	-4.219300
H	-1.161121	2.742544	-3.976057
C	-2.192446	1.734616	-1.825914
C	-3.463734	1.581138	-2.396758
H	-3.693404	0.686693	-2.976868
C	-4.435090	2.567523	-2.220042
H	-5.425492	2.436520	-2.658684
C	-4.138399	3.720966	-1.489229
H	-4.898549	4.490940	-1.350944
C	-2.870256	3.879838	-0.925950
H	-2.635041	4.768718	-0.341523
C	-1.904971	2.886117	-1.079400
H	-0.925747	2.999474	-0.611060
C	0.132275	-0.219740	3.143047
C	1.114656	-1.226734	3.108972
H	1.152805	-1.901279	2.249628
C	2.030626	-1.350440	4.151672
H	2.785225	-2.138257	4.119539
C	1.994744	-0.458949	5.230187
H	2.725847	-0.542730	6.035126
C	1.017327	0.536631	5.270475
H	0.980162	1.232659	6.109593
C	0.083027	0.651444	4.237463
H	-0.674261	1.433658	4.275281
C	-2.031184	1.333496	1.981758
C	-1.462183	2.606934	2.152836
H	-0.380592	2.733655	2.102826
C	-2.274756	3.713070	2.388308
H	-1.810918	4.689410	2.534771
C	-3.665556	3.573578	2.403225
H	-4.303140	4.443352	2.567165
C	-4.237127	2.317277	2.190351
H	-5.322003	2.203450	2.185649
C	-3.425774	1.197714	1.995115
H	-3.878840	0.216178	1.855248
H	2.874369	-0.226154	1.582843

8a•R (cont)

E = –4739.867844 a.u.

N_{imag} = 0

C	1.845863	3.645123	2.597053
C	1.375286	4.729777	1.850000
C	1.209757	4.565842	0.467831
C	1.507585	3.351584	-0.143076
C	1.967930	2.231788	0.594365
C	2.133197	2.423685	1.988155
H	1.989343	3.746526	3.674685
H	1.155749	5.684228	2.329045
H	0.856641	5.400217	-0.141975
H	1.394109	3.247045	-1.225592
C	2.236647	0.956209	-0.090098
C	3.268421	0.072790	0.599296
H	2.522938	1.166913	-1.131933
H	2.490849	1.603567	2.609598
C	4.608866	0.749320	0.863088
N	3.434076	-1.237502	-0.123945
C	5.259084	0.559293	2.089628
C	7.088792	1.977459	1.393603
C	5.213118	1.583642	-0.087039
C	6.444706	2.186295	0.170527
H	6.898168	2.835513	-0.580196
H	4.693628	1.788178	-1.023950
C	6.487497	1.166475	2.358349
H	4.778000	-0.058221	2.852403
H	8.045736	2.458941	1.599384
H	6.971021	1.015013	3.324851
C	0.702899	-3.111766	-1.116795
O	0.951567	-2.103330	-0.130754
H	1.650652	-3.383568	-1.615966
C	0.074039	-4.335186	-0.470028
H	0.031665	-2.709650	-1.893012
H	1.944162	-1.799423	-0.182305
H	-0.842199	-4.050265	0.063712
H	0.761588	-4.793282	0.255000
H	-0.188736	-5.085835	-1.230704
C	4.001721	-1.111829	-1.469384
C	4.195524	-2.213089	0.662301
H	5.053698	-0.773838	-1.452244
H	3.412888	-0.397858	-2.054759
H	3.959818	-2.091507	-1.965859
H	3.726427	-2.327670	1.648293
H	5.249555	-1.915500	0.807088
H	4.172034	-3.184810	0.149233

TS[**8a**•R–**5**•PA]

E = –4739.835749 a.u.

N_{imag} = 1

Cu	0.035550	0.127297	-0.073651
P	-1.096985	0.392762	-1.934665
P	-1.025650	-0.186526	1.848528
O	-2.541536	-1.478221	-0.325174
C	-1.710633	-1.222590	-2.526767
C	-1.499155	-1.756398	-3.803902
H	-0.997419	-1.153683	-4.560809
C	-1.899730	-3.062650	-4.090368
H	-1.719247	-3.477382	-5.082327
C	-2.525963	-3.846494	-3.116481
H	-2.821448	-4.866128	-3.360818
C	-2.777817	-3.335843	-1.838139
C	-2.357011	-2.029574	-1.580867
C	-3.508310	-4.073314	-0.714254
C	-3.506883	-5.589360	-0.927319
H	-2.485131	-5.991667	-0.955046
H	-4.010195	-5.846434	-1.868464
H	-4.059977	-6.093191	-0.124064
C	-4.973761	-3.561657	-0.679761
H	-5.007842	-2.474219	-0.533383
H	-5.520908	-4.040214	0.144787
H	-5.478768	-3.798741	-1.626936
C	-2.833610	-3.676795	0.601822
C	-2.395202	-2.354988	0.736194
C	-1.767244	-1.860328	1.886867
C	-1.616303	-2.738631	2.968805
H	-1.123444	-2.387764	3.874849
C	-2.050829	-4.059977	2.870946
H	-1.915679	-4.737208	3.714491
C	-2.642147	-4.528307	1.694160
H	-2.955367	-5.569741	1.631176
C	-0.226493	1.097194	-3.371835
C	1.032425	0.560131	-3.694898
H	1.414206	-0.293660	-3.131174
C	1.795353	1.123259	-4.715864
H	2.769269	0.697788	-4.961126
C	1.322133	2.242978	-5.408744
H	1.927833	2.694863	-6.195045
C	0.076864	2.785493	-5.085135
H	-0.294119	3.660376	-5.620713
C	-0.699701	2.212966	-4.074396
H	-1.670470	2.639453	-3.820180
C	-2.621275	1.377959	-1.758941
C	-3.806088	1.055773	-2.435324
H	-3.828884	0.199251	-3.110368
C	-4.956590	1.819100	-2.231658
H	-5.879616	1.557196	-2.750813
C	-4.926168	2.913919	-1.363388
H	-5.827313	3.506677	-1.201054
C	-3.744422	3.241193	-0.694436
H	-3.718498	4.083788	-0.004462
C	-2.598023	2.470179	-0.880555
H	-1.681271	2.704615	-0.337981
C	0.097488	-0.193799	3.287085
C	1.289870	-0.925306	3.129737
H	1.453979	-1.490275	2.205301
C	2.259312	-0.893028	4.129878
H	3.183010	-1.459732	4.002659
C	2.062955	-0.120812	5.280601
H	2.834077	-0.080037	6.050966
C	0.877898	0.600171	5.438056
H	0.718307	1.201230	6.334337
C	-0.108715	0.558139	4.448765
H	-1.030555	1.125906	4.574656
C	-2.390524	0.943845	2.261965
C	-2.080566	2.300270	2.458585
H	-1.043955	2.635347	2.399377
C	-3.093276	3.222359	2.710451
H	-2.835815	4.270008	2.873528
C	-4.430155	2.811709	2.727345
H	-5.224599	3.537069	2.907152
C	-4.744825	1.470859	2.499318
H	-5.786077	1.146051	2.498640
C	-3.730369	0.536199	2.279711
H	-3.981165	-0.512620	2.119695
H	3.427164	-0.096893	1.349374

TS[**8a**•R–**5**•PA] (cont)

E = –4739.835749 a.u.

N_{imag} = 1

C	1.274472	3.447159	2.270535
C	0.627719	4.385509	1.456990
C	0.518820	4.126588	0.086445
C	1.054219	2.960381	-0.457895
C	1.706728	1.992327	0.346047
C	1.797178	2.274529	1.731656
H	1.378691	3.631775	3.341227
H	0.230839	5.308411	1.881187
H	0.034045	4.851034	-0.570687
H	1.004098	2.796795	-1.536449
C	2.319098	0.785713	-0.267023
C	3.650260	0.360043	0.370686
H	2.444059	0.964010	-1.343697
H	2.299391	1.569059	2.391843
C	4.619811	1.505140	0.632536
N	4.257847	-0.736351	-0.417758
C	5.382303	1.526070	1.807420
C	6.486291	3.561848	1.110937
C	4.800437	2.540678	-0.295191
C	5.725765	3.557601	-0.062212
H	5.845781	4.359186	-0.792874
H	4.186008	2.562912	-1.196094
C	6.308616	2.543200	2.049399
H	5.227753	0.741884	2.552532
H	7.203323	4.362420	1.297579
H	6.885443	2.546204	2.975793
C	1.467851	-2.569851	-1.011120
O	1.284430	-1.658387	0.052739
H	2.550860	-2.728257	-1.175443
C	0.781947	-3.893181	-0.697333
H	1.055122	-2.168907	-1.962057
H	1.802568	-0.542158	-0.163079
H	-0.294271	-3.740869	-0.541337
H	1.195685	-4.329442	0.223125
H	0.912481	-4.614274	-1.519488
C	5.207386	-1.527873	0.344472
C	4.799108	-0.353299	-1.711769
H	4.726785	-1.882966	1.267489
H	6.133066	-0.977544	0.621428
H	5.502836	-2.406770	-0.247726
H	5.717005	0.269049	-1.648762
H	4.050729	0.207323	-2.287057
H	5.043446	-1.265903	-2.275532

5•PA

E = –4739.880497 a.u.

N_{imag} = 0

Cu	0.956449	-0.470596	0.062161
P	-0.207320	-0.247951	-1.914717
P	-0.216030	-0.794626	1.941587
O	-1.548415	-2.191300	-0.168566
C	-0.794174	-1.920015	-2.395135
C	-0.627852	-2.455646	-3.679302
H	-0.167113	-1.847244	-4.456466
C	-1.018220	-3.765627	-3.953845
H	-0.871456	-4.174978	-4.953660
C	-1.591806	-4.559783	-2.956446
H	-1.884441	-5.582576	-3.190726
C	-1.795778	-4.054952	-1.669191
C	-1.384859	-2.738948	-1.426138
C	-2.486378	-4.812204	-0.533035
C	-2.425959	-6.329541	-0.735916
H	-1.388556	-6.688764	-0.771035
H	-2.927947	-6.615830	-1.669086
H	-2.949583	-6.849674	0.076507
C	-3.971356	-4.359524	-0.492463
H	-4.046855	-3.274511	-0.342556
H	-4.496378	-4.859001	0.334186
H	-4.469913	-4.615465	-1.438325
C	-1.831353	-4.385323	0.781637
C	-1.406912	-3.059210	0.898330
C	-0.831438	-2.526143	2.059547
C	-0.756587	-3.359120	3.183222
H	-0.350287	-2.972255	4.116665
C	-1.173155	-4.687972	3.102077
H	-1.094158	-5.332712	3.977870
C	-1.687425	-5.201938	1.908024
H	-1.994894	-6.246118	1.864877
C	0.845659	0.188205	-3.349832
C	2.088290	-0.465431	-3.441768
H	2.338304	-1.209167	-2.672641
C	2.962992	-0.147814	-4.480172
H	3.925441	-0.658047	-4.546312
C	2.619778	0.824341	-5.426342
H	3.312409	1.076957	-6.230386
C	1.382258	1.465348	-5.341488
H	1.101738	2.215917	-6.081993
C	0.492277	1.142724	-4.313435
H	-0.479755	1.633643	-4.264955
C	-1.707453	0.776532	-2.193833
C	-2.953413	0.225053	-2.527908
H	-3.037055	-0.845987	-2.712321
C	-4.086382	1.037149	-2.621649
H	-5.048287	0.590375	-2.877565
C	-3.992019	2.412266	-2.394750
H	-4.878331	3.043449	-2.469893
C	-2.753706	2.973358	-2.065522
H	-2.660033	4.044512	-1.882653
C	-1.627804	2.160689	-1.956988
H	-0.677132	2.616105	-1.682658
C	0.701990	-0.529468	3.499552
C	1.681361	-1.445246	3.923552
H	1.805482	-2.392661	3.400619
C	2.528821	-1.138333	4.987228
H	3.281429	-1.863477	5.299876
C	2.427639	0.094536	5.638109
H	3.097480	0.336367	6.464096
C	1.467754	1.016244	5.214768
H	1.386086	1.989913	5.699211
C	0.612095	0.708274	4.157488
H	-0.110606	1.450280	3.823242
C	-1.822666	0.054477	2.236764
C	-2.498507	0.589662	1.137023
H	-2.037879	0.537618	0.154911
C	-3.748522	1.191266	1.290552
H	-4.254045	1.603228	0.416793
C	-4.329523	1.271702	2.557639
H	-5.300354	1.752190	2.686969
C	-3.664965	0.727685	3.662574
H	-4.119907	0.777825	4.653014
C	-2.424709	0.109938	3.502738
H	-1.919764	-0.323834	4.366266
H	1.416965	2.057894	-1.501152

5•PA (cont)

E = –4739.880497 a.u.

N_{imag} = 0

C	-0.690344	3.249365	2.017118
C	-0.231460	3.988107	3.110331
C	1.142360	4.133021	3.317578
C	2.044618	3.564555	2.417157
C	1.596947	2.847127	1.301689
C	0.219210	2.677222	1.126713
H	-1.759122	3.109477	1.858135
H	-0.943130	4.440559	3.802001
H	1.512494	4.695763	4.175611
H	3.118732	3.691610	2.570270
C	2.589867	2.342964	0.272152
C	2.205567	2.746709	-1.161368
H	3.572761	2.800608	0.516374
H	-0.145648	2.092287	0.283470
C	1.759779	4.170294	-1.397036
H	3.058778	2.548951	-1.826142
C	2.127908	5.244319	-0.575992
C	0.894354	6.795328	-1.971365
C	0.971760	4.442545	-2.526357
C	0.538541	5.737409	-2.811612
H	-0.078703	5.919676	-3.692706
H	0.706298	3.622252	-3.195334
C	1.697376	6.542722	-0.857658
H	2.745866	5.071151	0.303425
H	0.552818	7.808707	-2.185729
H	1.988783	7.360745	-0.197293
C	2.324446	-3.766179	0.841135
C	1.865844	-3.294347	-0.540104
O	2.041760	-1.930742	-0.746089
N	2.765906	0.858009	0.351923
C	3.278493	0.498275	1.687397
C	3.775992	0.398985	-0.624265
H	2.598749	0.848908	2.467105
H	3.360717	-0.592612	1.745048
H	4.278781	0.945173	1.857508
H	3.862744	-0.689175	-0.524602
H	3.451029	0.600812	-1.646984
H	4.748339	0.897879	-0.440023
H	1.712771	-3.284303	1.615902
H	2.215501	-4.856988	0.956224
H	3.377210	-3.494689	1.011438
H	0.806769	-3.616761	-0.671446
H	2.435251	-3.862430	-1.310444
